# Exploring Notch Pathway to Elucidate Phenotypic Plasticity and Intra-tumor Heterogeneity in Gliomas

**DOI:** 10.1038/s41598-019-45892-8

**Published:** 2019-07-01

**Authors:** Saikat Chowdhury, Ram Rup Sarkar

**Affiliations:** 1 Chemical Engineering and Process Development Division, CSIR-National Chemical Laboratory, Pune, Maharashtra 411008 India; 2 Academy of Scientific & Innovative Research (AcSIR), CSIR-NCL Campus, Pune, India

**Keywords:** Logic gates, Cellular signalling networks

## Abstract

The phenotypic plasticity and self-renewal of adult neural (aNSCs) and glioblastoma stem cells (GSCs) are both known to be governed by active Notch pathway. During development, GSCs can establish differential hierarchy to produce heterogeneous groups of tumor cells belong to different grades, which makes the tumor ecosystem more complex. However, the molecular events regulating these entire processes are unknown hitherto. In this work, based on the mechanistic regulations of Notch pathway activities, a novel computational framework is introduced to inspect the intra-cellular reactions behind the development of normal and tumorigenic cells from aNSCs and GSCs, respectively. The developmental dynamics of aNSCs/GSCs are successfully simulated and molecular activities regulating the phenotypic plasticity and self-renewal processes in normal and tumor cells are identified. A novel scoring parameter “Activity Ratio” score is introduced to find out driver molecules responsible for the phenotypic plasticity and development of different grades of tumor. A new quantitative method is also developed to predict the future risk of Glioblastoma tumor of an individual with appropriate grade by using the transcriptomics profile of that individual as input. Also, a novel technique is introduced to screen and rank the potential drug-targets for suppressing the growth and differentiation of tumor cells.

## Introduction

The sub-ventricular zone (SVZ) of human brain, recognized as the primary location for nurturing neural stem cells (NSCs), also provides a sustainable ground for the development of matured Glioblastoma (GBM) tumor cells^1^. The GBM tumor ecosystem is highly heterogeneous and complex, which also accompanies with multiple non-tumorigenic cells, e.g., quiescence, apoptotic (or necrotic), normal neurons, astrocytes, oligodendrocytes^2^. The glioblastoma stem cells (GSCs), a distinct sub-population within the GBM tumor ecosystem, show phenotypic plasticity and develop intra-tumor heterogeneity within GBM tumor cells. GSCs are also considered to be the major cause of tumor recurrence, and establishment of the lineages of drug-resistant tumor cells^3^. It is reported in a recent finding that the mosaic expression pattern of *NOTCH* receptor gene (a non-RTK surface gene/protein) and its downstream reaction cascades are also interesting to realize the phenotypic plasticity and the development of intra-tumor heterogeneity of GBM tumor and tumor initiating stem cells^2^.

Notch signaling pathway, which is commonly known for the maintenance and proliferation of the adult neural stem cells (aNSCs) in the neurogenic niche of SVZ, is also implicated in the proliferation of GSCs^1^. It suppresses the neurogenesis and gliogenesis by targeting the expression of bHLH transcription repressor proteins HES1-7 and HEY1, 2, L, which in turn help to maintain the self-renewal of aNSCs/GSCs by suppressing the transcriptions of the differentiation genes^4^. On the contrary, the other component proteins of Notch pathway (e.g., NOTCH1, RBPJ/CSL, NICD1, γ-Secretase complex, etc.) are observed to be over-expressed in the differentiated astrocytes and GBM tumor cells^5,6^. Moreover, how Notch pathway reprograms its functional mechanisms to trigger the differentiation processes in aNSCs/GSCs, despite being the gate-keeper of the maintenance of stem-like cells, is unknown hitherto. Previous reports suggest that several cross-talk pathways can manipulate the Notch signaling to suppress its effects from the self-renewing processes of aNSCs/GSCs and trigger the differentiation of normal cells or astrocytomas^7,8^. Also, the mechanistic regulations through which the cross-talk molecules influence the dynamics of Notch pathway and help to sustain its dichotomous nature between stem cells renewal and differentiation (i.e., neurogenesis and gliogenesis) processes are also not clearly understood yet.

Various experimental analyses have corroborated the existence of the driver genes/proteins (e.g., P53) causing de novo tumorigenesis, but a follow-up study is essential for studying their functional relationships with core Notch signaling network to understand the regulation of self-renewal and phenotypic plasticity of GSCs, and the development of primary tumor cells^9^. Simultaneously, the molecular processes which trigger the formation of neurosphere from normal aNSCs or the GSCs present in the GBM tumor population are also required to be studied^4^. Moreover, the underlying mechanisms of Notch pathway regulating the differential hierarchy to develop different grades (low and high grades) of primary GBM tumor cells are also not explored yet. More importantly, previous reports also suggest that a mixed population of GBM tumor cells belong to high or low grades along with other cell lineages (e.g., neurons, astrocytes, quiescence cells, etc.) in the tumor niche and impose significant hurdles in GBM therapeutics^10–13^. Hence, there is a genuine need to understand the molecular events responsible for the development of phenotypically distinct tumor cells with different grades, phenotypic plasticity and self-renewal of tumor stem cells, and the emergence of heterogeneous population of tumor cells in the same grade of tumor cells.

The molecular mechanisms through which the phenotypic plasticity and stem cell renewal processes of GSCs are regulated could be explored with higher resolutions by analyzing the proliferations and differentiation dynamics of aNSCs and GSCs in an in-silico environment. Moreover, the differential hierarchy established by GSCs to develop the heterogeneous groups of matured GBM tumor cells within the similar grade of tumor could also be dissected by utilizing “cancer stem cell model” proposed to describe the emergence of intra-tumor heterogeneity^10,14^. In this work, it is hypothesized that understanding the molecular mechanisms of Notch and its cross-talk signaling cascades may help to shed light on these unexplored phenomena in more details and could solve various unanswered questions related to neurogenesis and GBM tumorigenesis^15^.

Hence, by considering the dynamic activities of Notch and its cross-talks pathways, in this present work, predictive mechanistic models are developed for the analyses of the underlying mechanisms of neurogenesis and GBM tumorigenesis. The underlying molecular processes of Notch and its cross-talks pathways are attempted to be explored by using computational modeling approach, which in turn helps to assess the governing principles working behind the self-renewal, differentiation, apoptosis, and cell growth arrest (i.e., quiescent state) of aNSCs/GSCs or GBM tumor cells. Also, by assessing the variances in the expressions/activities of the genes/proteins considered in the individual tumor cells, this work is competent to dissect the underlying mechanisms working behind the emergence of different sub-types and heterogeneous populations of GBM tumor cells. Based on these basic understandings, these models are further implicated to perform futuristic predictions of the transformation of low-grade astrocytoma to high-grade GBM, and the chances of occurrences of heterogeneous populations of GSCs and matured GBM cells in the GBM ecosystem. Apart from these analyses, the developed models are also used to explore other fundamental aspects of neurogenesis and tumorigenesis, for example, what are the molecular mechanisms driving the excess rate of apoptosis of aNSCs observed during neurogenesis?^16^. Is there any role played by Notch pathway during apoptosis and cell growth arrest (or quiescent state) of aNSCs/GSCs? Does P53 mutation hinder the neurogenesis or other cellular differentiation of GSCs? What are the main component proteins of Notch pathway, which form the regulatory switch to regulate the differentiation of different cell lineages from aNSCs/GSCs?

The models are developed to predict the probability of the emergence of GSCs within the normal neurogenic niche and identify the driver proteins responsible for the development of different tumor sub-types. The chances of developing different grades of primary GBM tumor cells from GSCs or mutated astrocytes cells are also quantified and further applied as a personalized approach to detect the risk of early onsets of GBM tumor with appropriate grades (or sub-types). A novel scoring technique is introduced here for quantifying the risk of the tumor development by considering the genetic variances of the individual patients as inputs. Moreover, a novel approach is developed for in silico perturbations analyses to perform the assessments of targeting multiple combinations of proteins on the developed models. The implication of this approach would help to screen and identify potential molecules for target-based GBM therapeutics.

## Results

### Mechanistic model of aNSC developmental dynamics correctly captured the probable fates of the adult neural stem cells

The mechanistic model for simulating the aNSC renewal and differentiation dynamics was constructed by translating the biochemical reaction cascades of the reconstructed Notch and its cross-talk pathways into a system of coupled logical equations (Supplementary Table 1). The input of this model is the binary expression states (1 or 0) of all the input molecules (i.e., proteins and metabolites) (Supplementary Table 2), which could be obtained from the transcriptomics, proteomics, metabolomics data describing the expression or activity (e.g., ON/Up-regulated and OFF/Down-regulated states) levels of the input molecules in aNSCs. In such case, the modeling strategy will be more closed to the context (e.g., cell lines) specific simulation of the individual biological cells (see Supplementary Information).

On the other hand, the binary states of each input molecules can be considered completely random during simulation. In this work, one of the objectives of developing the logical models was to explore the activities of all possible combinations of the input molecules on the development of normal and tumor cells. Hence, the initial values (binary states) of all the input molecules were considered randomly to simulate the aNSC model and thus the developmental dynamics of aNSCs under the exposure of a variety of input signals were analyzed. The expected outcomes of this model simulation were the probable appearances of common phenotypic plasticity and stem cell regeneration processes observed in the developing aNSCs present in the SVZ of human brain^17^.

The simulation outcomes of aNSC model with highest Shannon entropy score (1.456 Shannon) had produced multiple cellular states in the steady state or attractor space (Supplementary Fig. 1A). The observed cellular states were further considered as different cell lineages of normal brain cells, such as quiescent, neural stem cells, neuron, and astrocytes progenitor cells (Supplementary Fig. 1B). The temporal expressions of the marker proteins mapped with each cellular state are shown in Supplementary Figs S2 and S3. It was observed that a large number of attractors had active pro-apoptotic marker proteins expressed in the steady-state, which in turn caused a large numbers (more than 50%) of cellular states to undergo the apoptotic state in the attractor space (Supplementary Fig. 1B). This result justifies the previous experimental outcomes, where it is shown that most of the aNSCs undergo apoptotic state during the normal stem cell developmental process to maintain a sustainable population of aNSCs in human brain (see Supplementary Information)^16^. Followed this, further studies were performed to extract the activation signals (i.e., inputs), which trigger either the apoptotic cascades in the developing aNSCs or divert the stem cells towards the development of other cell lineages. A novel scoring parameter named “Activity Ratio (AR)” score was introduced for this purpose (see Supplementary Information).

**Figure 1:**
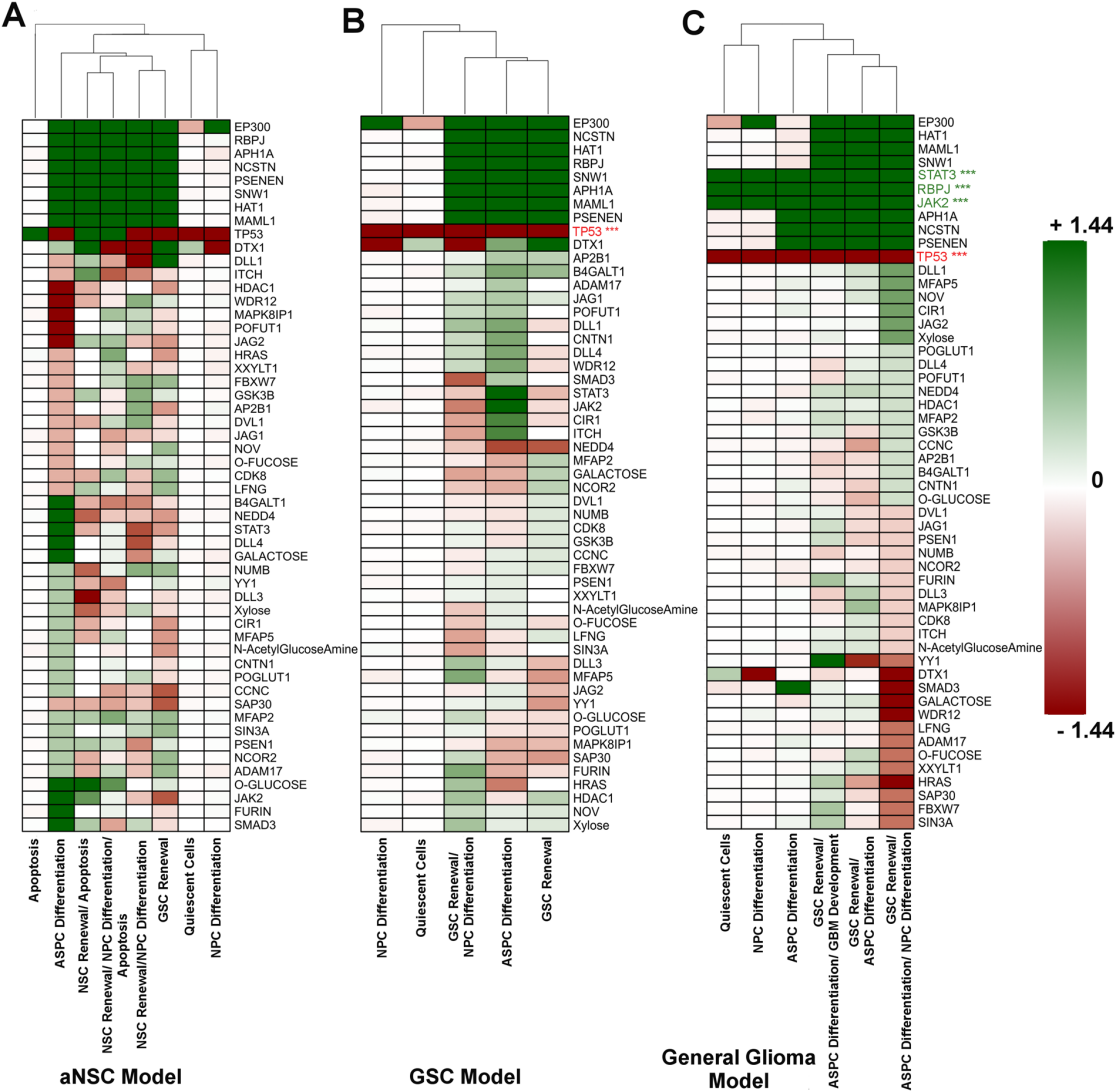
Activity Ratio (AR) scores of the input molecules of (**A**) aNSC (**B**) GSC and (**C**) GBM Model simulations. The AR-scores of each input molecules were calculated for every cellular state. TP53 protein was found as mutated in the “GSC Model” simulation. On the other hand, while simulating “GBM Model”, TP53 protein was found mutated along with increased activities of RBPJ+/+, JAK2+/+ and STAT3+/+ proteins.

The activity ratio (AR) scores were computed for each of the input proteins of aNSC model simulation (Fig. 1). The AR profile showed that during the normal neural cell development, the aNSCs which constitutively expressed the wild-type P53 protein (AR score = +1.44) but did not express Notch pathway inducer proteins, such as APH1A, NCSTN, PSENEN, JAG1, DLL1, etc. may undergo apoptotic process due to the active P53 pathway and inactive Notch signaling cascade (Fig. 1). In contrasts, it was observed that the aNSCs, which had expressed all Notch pathway inducer proteins and active wild-type P53 protein (*TP53*), were able to produce CCND1, CCND3, and CDK2 for maintaining the stem cell renewal process followed by the expressions of pro-apoptotic proteins (e.g., BAD, BAX, NOX, PUMA) mediated by P53 pathway (Fig. 1). It was also seen that aNSCs with P53 deficiency (or inactive P53 with AR score = −1.44) and inactive Notch pathway inducer proteins as well as with active EP300 and inactive DTX1 proteins had lost the stemness property and differentiated into neurons or NPCs (Fig. 1). On the other hand, the development of bi-potent cells (aNSC/NPC) with the markers of both aNSCs and NPCs were also observed in the simulation outcomes if the developing stem cells had P53 deficiency but had both the active Notch signaling and EP300 proteins (Fig. 1).

Further study was also performed to find out the reaction motifs within the Notch signaling and its cross-talk pathways which were getting influenced by these different combinations of inputs (or activation) signals and finally induce such phenotypic plasticity during aNSC development (Supplementary Fig. 5). Hence, it is proven from these analyses that the stem cell renewal induced by Notch pathway and its antagonistic effects exerted on P53 pathway is useful to maintain the regenerative property of the stem cells (Supplementary Fig. 5)^18,19^. On the other hand, the P53 mediated pathway is found to inhibit the Notch pathway activation in various cells, which in turn proves the interplay between these two pathways in the regulation of apoptosis and cellular development^20^. Simulation outcomes of aNSC model also agree with this experimental evidence, in which P53 activation is found to be associated with the increased apoptosis and suppression of aNSC regeneration induced by Notch pathway activation. The counteractive axes formed between P53 and Notch pathways are required to maintain the proper balance in the population growth of aNSCs within CNS^16^. Also, it was observed from the AR profile of aNSC model simulation that the activation of the Notch pathway inducer proteins with constitutive expressions of JAK2/STAT3 proteins were responsible for the development of astrocytes progenitor cells (ASPCs) originating from the aNSCs (Fig. 1). The molecular reaction mechanisms identified in the pathway model also revealed that the interaction between Notch target proteins HES1, HES5 with JAK/STAT pathway promoted the expression of GFAP protein, a known marker of glial cells, such as astrocytes (Supplementary Fig. 1A)^21,22^.

### P53 mutation induces the development of glioma stem cells (GSCs) from proliferating aNSCs

It was observed from the AR profile of aNSC model simulation that if the inducer proteins of Notch pathway (e.g., APH1, RBPJ, MAML1, etc.) were constitutively active (AR score = +1.44) and simultaneously the P53 protein was inactive (AR score = −1.44), then the self-renewing aNSCs will undergo towards the development of GSCs (Fig. 1). However, it was observed that the probability of this combination (i.e., protein activities) was infrequent in proliferating aNSCs, and thus the normalized frequency obtained for proliferating GSCs was lowest in the simulation outcomes (Supplementary Fig. 1B). The reaction motif responsible for the development of GSCs from aNSCs is shown in Supplementary Fig. 5D.

Furthermore, another new model was constructed by introducing the P53 mutation (i.e., keeping it at constitutively inactive state) and the identified reaction motif of GSC development (Supplementary Fig. 5D) in the original aNSCs model. This new model was named as GSC developmental model, in which the developing GSCs with P53 deficiency and with the reaction motif of GSC renewal process were exposed to variety of input signals during simulation (see Supplementary Information). It was observed that P53 mutation in GSCs did not influence the developmental dynamics of the stem cells and the cells still retained its self-renewal and differentiation properties like aNSCs. Simulation outputs of GSC model (highest Shannon entropy 0.852 Shannon) were able to exhibit the similar phenotypic plasticity and self-renewal properties observed in the neurogenic development (e.g., neurons, astrocytes, quiescent cells, etc.) of aNSC model, except the development of apoptotic cells (Supplementary Fig. 1). Apoptosis was missing in this simulation due the induction of P53 mutation in the GSCs.

### Cross-talks of active notch signaling with JAK/STAT pathway in developing GSCs promoted the development of glioma cells

Further analyses of AR-scores revealed that increased activities of JAK2 and STAT3 proteins including the core Notch pathway proteins were able to stimulate the GSCs differentiation towards the development of mutated astrocytes without any apoptotic break (Fig. 1). These cells were further identified as the astrocytomas or low-grade glioma (LGG) by assessing the expression pattern of the corresponding marker proteins. This observation clearly indicated the origin of primary tumor cells (LGG) developing from P53 mutated GSCs in the GSC model simulation. Hence, to capture the effect of phenotypic plasticity of GSCs in which JAK2/STAT3 proteins are constitutively active, a new model was further formulated by introducing P53 mutation and over-expressions of JAK2 and STAT3 proteins in the cells used for GSC model simulation (see Supplementary Information).

During the simulation of this new model, the mutated GSCs were exposed to a variety of activation signals just like the previous aNSC and GSC models. The simulation outcomes had shown that the GSCs did not lose its phenotypic plasticity despite of having over-expressed JAK2/STAT3 proteins and was still able to generate NPCs. It was also able to maintain its stemness property and simultaneously produced different types of mutated primary astrocytes or glioma cells in the steady state (Supplementary Fig. 1B). Therefore, this new model was named as general glioma model. It was further observed in general glioma model that the LGG phenotype or cellular state could be further characterized into two sub-types of primary glioma cells *viz*. LGG-I and LGG-II states. It was found that in LGG-I (i.e., ASPC differentiation) cellular state, none of the high-grade GBM cells markers, e.g., MYC or TENASCIN-C proteins were highly expressed (Supplementary Fig. 4). On the other hand, in LGG-II sub-type (i.e., ASPC differentiation with GSC renewal), the increased activity of the protein TENASCIN-C was only observed, which in turn made this tumor sub-type more proliferative (due to stem-like property), aggressive, and lethal than the LGG-I sub-type. Apart from these low-grade glioma sub-types, differentiating GSCs were also directed towards the development of primary (de novo) high-grade or “Grade-IV” tumorigenic cellular state in the general glioma model simulation (highest Shannon entropy score, 1.164 Shannon). Grade-IV GBM state had higher abundances of both the metastatic and aggressive high-grade GBM marker proteins MYC and TENASCIN-C (Supplementary Fig. 4). After analyzing the AR-scores of the input molecules, it was observed that YY1 transcription factor was one of the driver proteins associated with the development of Grade-IV tumorigenic state in the simulation and eventually formed a nexus of the transcriptions regulatory genes associated with tumorigenesis and metastasis. These overall simulation studies on aNSC, GSC, and general glioma models had revealed the underlying molecular reaction mechanisms in Notch signaling pathway through which the phenotypic plasticity and self-renewal processes of aNSCs or GSSs are regulated during the developmental stages (Supplementary Fig. 5).

### Increased activities of JAK2/STAT3, RBPJ, YY1, γ-Secretase complex and P53 mutation promoted the development of primary high-grade GBM

**Figure 2:**
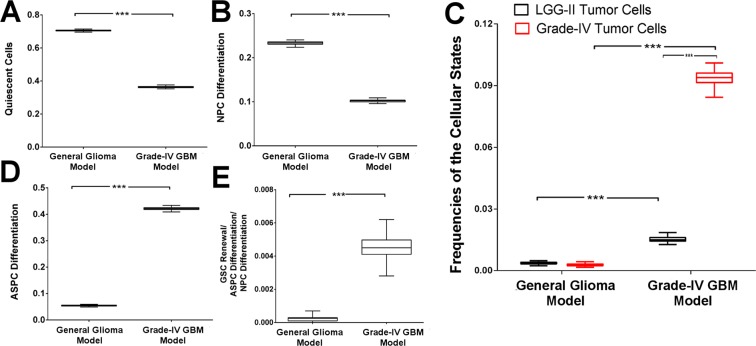
Comparative analyses of the normalized frequencies of cellular states observed in general glioma and Grade-IV models. Normalized frequencies of the cellular states representing (**A**) Quiescent cells; (**B**) NPC Differentiation; (**C**) LGG-II vs. Grade-IV tumor cells; (**D**) ASPC Differentiation or LGG-I; and (**E**) GSC Renewal/ASPC/NPC Differentiation states observed between general glioma and Grade-IV GBM models are compared here. The simulations of all the models were replicated 100 times (N = 100). Data represents the means ± S.D.; ***P-value < 0.001.

In the high-grade (Grade-IV) GBM tumor states of general glioma model, increased activities of JAK2/STAT3, RBPJ, MAML1, YY1, γ-Secretase complex (APH1, NCSTN, PSENEN, PEN2) and mutation of P53 were observed as a minimal number of deregulations (total mutations, μ = 10). Hence, a new model was built by constitutively expressing Notch pathway molecules (RBPJ, MAML1, γ-Secretase complex), JAK2/STAT3 and YY1 proteins and by mutating P53 protein in the GSCs to simulate the differentiation of primary GBM (Grade-IV) cells, and observed how much the model simulation having these higher number of mutations induce the development of GBM cells. As expected, in this new model the mean normalized frequency of the Grade-IV GBM tumor state was found comparatively high (Mean 0.094 ± 0.003 S.D.) as compared to the normalized frequency of (Mean 0.002 ± 0.0005 S.D.) primary Grade-IV GBM cells observed in general glioma model. Thus, this new model was named as high-grade (Grade-IV) GBM model. A comparative study, performed to analyze the changes of the mean normalized frequency differences of different cellular states observed in general glioma and Grade-IV GBM models, depicted significant changes in all scenarios (Fig. 2).

In the Grade-IV GBM model, the normalized frequencies of quiescent cells and the differentiating NPCs were found to be significantly reduced in numbers (Fig. 2A,B). In contrast, the normalized frequencies of differentiated ASPCs (LGG-I state) and the cellular state “GSC Renewal/ASPC/NPC Differentiation” were significantly increased in the Grade-IV GBM model as compared to general glioma model (Fig. 2D–E). A significant increase of LGG-I cells depicted the possibilities of the presence of spatiotemporally distributed, genetically distinct, heterogeneous populations of differentiated astrocytes cells within the high-grade (Grade-IV) GBM tumor niche. Most often these cells (LGG-I) progress to form high-grade (Grade-IV) glioblastoma tumor cells and cause poor prognosis of the patients^23^. It was observed that in the general glioma model, although a significant difference between LGG-I and Grade-IV cells (Supplementary Fig. 1B) exists, no difference was found between LGG-II and Grade-IV cells (Fig. 2C). In contrasts, the simulation outcome of high-grade (Grade-IV) GBM model showed a significant differences between the frequencies of the two states: LGG-II and Grade-IV. A higher number of Grade-IV tumorigenic cells were observed, which in turn justified the redirection of low-grade GBM cells towards the high-grade state in this high-grade GBM model (Fig. 2C).

### Origin of intra-tumor heterogeneity and distinct sub-types of GBM tumor

**Figure 3:**
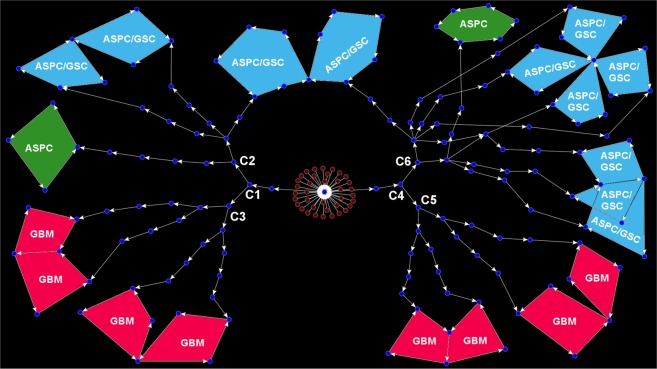
Stage transition graph (STG) of the development of distinct sub-types of GBM. The Red and blue nodes represent the initial and transient cellular states of the general glioma developmental model. It shows that starting from a group of common tumor-initiating cells (red colored nodes), the tumorigenic developmental process leads towards distinct grades (sub-types) and heterogeneity in the GBM tumor cells. ASPC and ASPC/GSC refer the cyclic attractor states “ASPC Differentiation or LGG-I” and “GSC Renewal/ASPC Differentiation or LGG-II” respectively, whereas GBM refers the cellular state corresponding to “GSC Renewal/ASPC Differentiation/GBM Development or Grade-IV” cellular state.

The state transition graph (STG) of the logical model developed for general glioblastoma development (GBM) was used to identify the initial and transient states (or cells), which were eventually redirected at either the singleton (i.e., fixed-point) or periodic (i.e., cyclic) attractor states. Here, STG was used for representing the differential hierarchy established by the tumor stem cells during its proliferation and differentiations. In the steady state level of general glioma model simulation, the constructed STG contained 26 distinct groups of clusters, each of which was associated with either fixed-point or cyclic attractor states or sometimes both (Supplementary Fig. 6). Analysis of one such cluster extracted from the STG showed that the developmental dynamics of the tumor-initiating mutated GSCs were able to develop any of the three different sub-types of GBM *viz*. LGG-I, LGG-II and Grade-IV^11,24^ (Fig. 3).

In this STG, the basin of attractors contained the critical nodes (e.g., C1, C2, C3, C4, C5, C6, etc.) at which the transient states were separated and proceed towards different cellular states (i.e., LGG-I, LGG-II, and Grade-IV). The critical nodes in STG, such as C1, C2, C4, and C6 are the nodes, at which the stimulated GSCs undergo asymmetric cell division and produce distinct sub-types of GBM cells. On the other hand, C3 and C5 represent the critical junctions during the development at which the transient tumorigenic stem cells perform symmetric cell divisions and produce heterogeneous group of tumor cells belong to different grades of GBM tumor (Fig. 3). For example, there were total 14 distinct heterogeneous groups of Grade-IV GBM tumor cells (periodic attractor states) obtained in the simulation of general glioma model, which had shown different protein activity patterns at the steady state (periodic) of the simulation (Supplementary Fig. 7). Comparative analyses of the activity patterns of the pathway molecules were performed successively to extract essential marker proteins whose variations of expressions were creating heterogeneities within the heterogeneous population of Grade-IV tumor cells.

This STG helped to track the small but effective fluctuations within the tumorigenic stem cells, which significantly contributed to the emergence of different cell types, grades and the intra-tumor heterogeneity previously observed in the GBM tumor cells niche^2^. For example, after the critical junction at C1 in STG, it is visible that the developmental paths lead to either ASPC (LGG-I) or GSC/ASPC (LGG-II) cellular states via the transient node C2. Otherwise, it can also be attracted towards high-grade GBM (Grade-IV) states via C3 junction (Fig. 3). Hence, by comparing the protein activity profiles of the transient nodes C2 and C3, it was revealed that transitions from low-grade GBMs (LGG-I or LGG-II) to high-grade GBM was possible if the cells had increased activities of the proteins YY1, C-MYC, CYCLIN-D1, CYCLIN-D3, etc. Comparing C5 and C6 nodes, the similar expression pattern of the proteins behind the origin of high-grade tumor cells was also observed. Moreover, to assess the molecular heterogeneities within the GBM cells, all the different 14 attractors states observed in Grade-IV tumor state were compared with each other, which in turn helped to understand the origin of the heterogeneous groups of GBM tumor cells (Supplementary Fig. 7).

### Understanding the bias of the outcomes of different cellular states under different conditions

The probabilities of occurrences of different cellular states were not homogenous in the attractor space. For example, in Grade-IV GBM model, the normalized frequency of Grade-IV tumor cells was comparatively higher than LGG-II, whereas, in the general glioma model, these two cellular states did not have much difference (Fig. 2C). Here, it is hypothesized that the intra-cellular network, through which the activation signal flows from the ligand and receptors to the marker proteins via the cytoplasmic and nuclear molecules, plays crucial roles to determine the bias towards a particular cellular state during development. During the flow of such signal, which is imposed on a cell at the time of cellular development, a set of signaling molecules (or proteins) alter their activity/abundance (ON/OFF states) patterns rapidly and relay the signal to the transcription factors in the nucleus. Hence, it could be considered that the activities of different ligand molecules, the complicated topology of the intra-cellular network, and the presence of several intrinsic and extrinsic fluctuations (e.g., mutations, over-activation, phosphorylation, etc.) were the critical factors behind the emergence of cellular heterogeneities in the attractor space. It is evident that if higher numbers of alteration of the molecular activities are required for reaching a particular cellular state or phenotype, then a cell has to perform multiple chemical reactions. It will eventually increase the overall costs for the cells at the developing stage and thus decrease the probability to reach that particular cellular state. Hence, to quantify the effects of diverse molecular events (e.g., chemical reaction, physical interaction, translocation, etc.) associated with the development of different cellular states, a novel phenotype cost function (ψ_*C*_) was defined in this present work. It was defined as the summation of the average rate of changes of the activity patterns of all the pathway molecules (i.e., the signaling cost) and the mutational cost associated with that cellular system (Supplementary Information).

**Figure 4:**
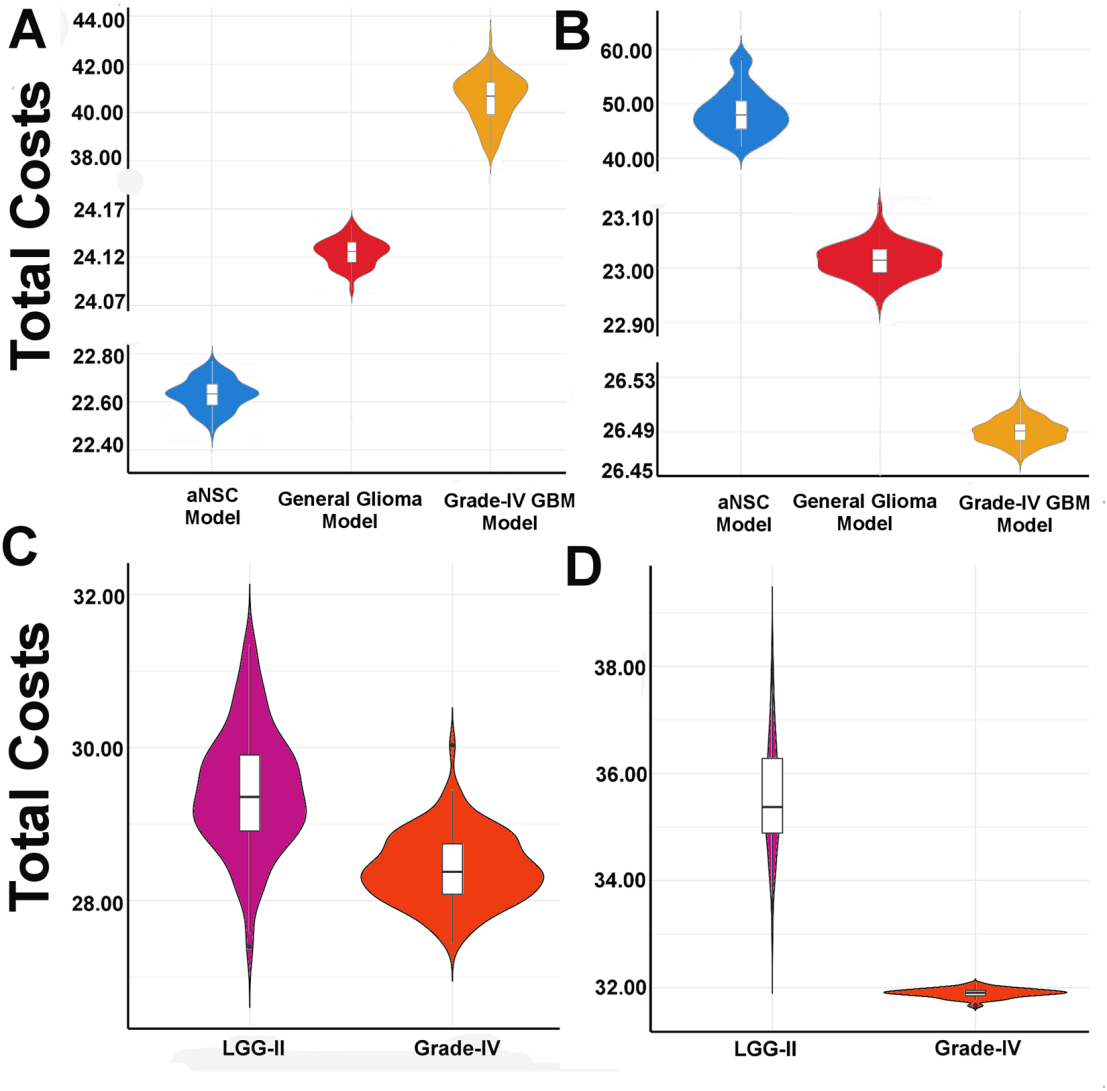
Violin plots of the total phenotype cost function or total costs calculated for different cellular states. Comparisons of the median and the probability density functions of the cellular states (**A**) NPC Differentiation and (**B**) ASPC Differentiation observed in aNSC, general glioma, and high-grade GBM models respectively. In high-grade (Grade-IV) GBM model, the median costs for reaching the differentiated NPC and ASPC cells are opposite to each other. In adult NSC model, the median costs for reaching the differentiated NPC cellular state is lowest compared to the others tumorigenic models. In contrasts, the required total cost is significantly lower in the tumorigenic GBM model as compared to the non-tumorigenic aNSC model. Comparative analyses are also performed between the total costs required for reaching the LGG-II and Grade-IV tumor cell states in (**C**) general glioma and (**D**) High-grade GBM models.

Analyses of the phenotype cost function were found to be very much useful to understand the bias towards the determination of particular cell fate during neural stem cell maintenance and differentiation processes. The violin plots depict the comparative analyses of the distributions of the average cost values associated with the cellular states “NPC Differentiation”, “ASPC Differentiation (LGG-I)”, “GSC Renewal/ASPC Differentiation (LGG-II)”, and “GBM Development (Grade-IV)” cells observed in aNSC (non-tumorigenic), general glioma and high-grade GBM (tumorigenic) models (Fig. 4). It was observed that in the non-tumorigenic, adult NSCs model, the overall median costs to produce the differentiated neurons (NPCs) was significantly lower than the costs required for producing differentiated astrocytes cells (ASPCs) (Fig. 4A,B). This result was validated with the previous observation, which suggests the higher amount of neurogenesis happens in the adult neural stem cell niche in SVZ^25^. Whereas in the general and high-grade GBM models, the total costs for reaching the ASPC differentiation state at the steady state level found to be reduced significantly, which in turn proved the previous experimental finding, which has shown the higher number of matured astrocytes cells also exist in GBM tumor niche^26^. Hence, it is proven that the induced mutations, e.g. P53 knock-out, and increased activities of JAK2/STAT3, RBPJ, YY1 proteins, etc. can divert the normal functioning of Notch pathway in the aNSCs from undergoing the stem cell renewal or neuronal differentiation processes to the development of mutated, differentiated astrocytes cells^26^.

On the other hand, while performing the intra-model comparisons of the total costs required for LGG-II and Grade-IV tumor cells, it was observed that in both the tumorigenic (i.e., general glioma and Grade-IV GBM) models, the median total costs required for developing Grade-IV tumor cells were comparably lower than the LGG-II cells. Although, the median difference was not very much high in general glioma model (Fig. 4C), but it was found to be significantly higher in high-grade (Grade-VI) GBM model. Hence, it proved that to reach the Grade-IV tumor state in comparison to the LGG-II cellular state at the steady state level, the high-grade (Grade-IV) GBM model requires less cost than LGG-II cellular state. This result also explained the previous observation that why the Grade-IV tumor cells were observed with higher normalized frequency in the high-grade (Grade-IV) GBM model (Fig. 2C).

### Applications of phenotype predictor scores to predict the appearances of cellular states

The normalized frequency (*P*_*C*_) and the total phenotype cost function or total cost (ψ_*C*_) required for reaching a particular cellular state (*C*) were the two main parameters, which showed to regulate the outcome of a specific cellular state during the cell fate determination process. It was observed that the probability of occurrences of a particular cellular state was directly proportional to its total frequency and inversely proportional to the phenotype cost function. A novel scoring function “Phenotype Predictor Score (Θ_*C*_)” was introduced here by defining this function as the ratio of the total frequency to the total phenotype cost function associated with the cellular states. The distributions of the frequencies (*P*_*C*_) and total phenotype cost function (ψ_*C*_) of all the cellular states in the attractor space followed normal distributions with different variances. Hence, the probability density function of this newly defined score (Θ_*C*_) is the joint probability distribution $({{\rm{\Theta }}}_{C}=\frac{{P}_{C}}{{{\rm{\Psi }}}_{C}})$ of the ratio of two multivariate normally distributed variables and the shape of the resultant distribution is a fat-tailed Cauchy-like distribution (Supplementary Fig. 8)^27^. A detailed description of this distribution including its mathematical expression is provided in the Supplementary Information (Supplementary Fig. 8).

A higher value of phenotype predictor score of an arbitrary cellular state signifies the greater chance of that cellular state to appear within the attractor space. The mean values with 95% CI (calculated using Fieller’s theorem^28^) of all the cellular states (total 12) observed in all the four aNSC, GSC, general glioma and Grade-IV models are provided in Supplementary Table 4. It was observed that the mean phenotype predictor score (mean 147.688, 95% CI [146.630 148.747]) of the “Quiescent” cellular state was lowest in the highly mutated Grade-IV GBM as compared to the other models. This result was also in accord with the previous observation (Fig. 2A), in which lower normalized frequency of quiescent state was detected in Grade-IV GBM model as compared to general glioma model (Fig. 2A). Therefore, it can be stated that the percentage of quiescent cells will be less in the heavily mutated tumorigenic niche (i.e., Grade-IV GBM cells) as compared to normal neurogenic niche (adult NSCs). A similar result was also observed for “NPC differentiation” state, which showed that in the same tumorigenic niche, the heavily mutated tumor cells were least influenced (mean 40.372, 95% CI [39.804 40.940]) to differentiate into matured neurons as compared to normal aNSCs in the neurogenic niche. The score observed for GSC renewal (mean 0.193, 95% CI [0.163 0.224]) state was also higher in the GSC model as compared to the aNSC model (mean 0.100, 95% CI [0.078 0.124]). A comparative analysis of LGG-I, LGG-II, and Grade-IV cellular states observed in general glioma, and Grade-IV GBM models revealed that the phenotype predictor scores for all of these cellular states were comparably higher in Grade-IV GBM model as compared to other model simulations. For example, the score observed for Grade-IV cellular state in general glioma model was less (mean 0.970, 95% CI [0.873 1.066]) as compared to Grade-IV GBM model (mean 29.414, 95% CI [28.875 29.955]). Thus, the induced alterations of the protein expressions in Grade-IV GBM models accelerated the growth of LGG-I, LGG-II and high grade (Grade-IV) glioblastoma cells, which in turn helped to predict the chances of occurrences of the oncogenic cells in the tumor ecosystem.

Based on these observations, it was ascertained that the phenotype predictor score could be used to highlight the possible outcomes of the specific grade of tumor cells in the tumorigenic niche and could be a good estimator to quantify and assess the heterogeneity of glioblastoma tumor cells. Hence, after its successful executions on the master tumorigenic models (i.e. general glioma and Grade-IV GBM), it was interesting to analyze the performance of this novel scoring function to detect and quantify different grades and molecular heterogeneity of tumor cells present in the tissue samples, collected from the individual glioblastoma patients. Here, the general hypothesis was that given the molecular expressions (e.g., transcriptomics, proteomics, etc.) data from the individual glioblastoma tumor patient or cohort, the phenotype predictor scores calculated for the tumorigenic (e.g., LGG-I, LGG-II, Grade-IV, etc.) and non-tumorigenic (e.g., Quiescent, NPC differentiation, apoptosis, etc.) cellular states from the tumorigenic model would be able to predict the chances of occurrences of tumor cells and the corresponding grades. The RNA-Seq transcriptomics data, available in The Cancer Genome Atlas (TCGA) data portal, extracted from the P53 mutated low (TCGA-LGG) and high grade (TCGA-GBM) tumor samples cohorts of glioblastoma patients (Supplementary Table 5) were used here for the case study to assess the potential of the developed models for identifying tumor grades by using the newly introduced estimator, “phenotype predictor score”. A detailed description of the entire methodology used in this case study is provided in Supplementary Information.

### A case study with the low and high- grade glioblastoma patient’s cohort

**Figure 5:**
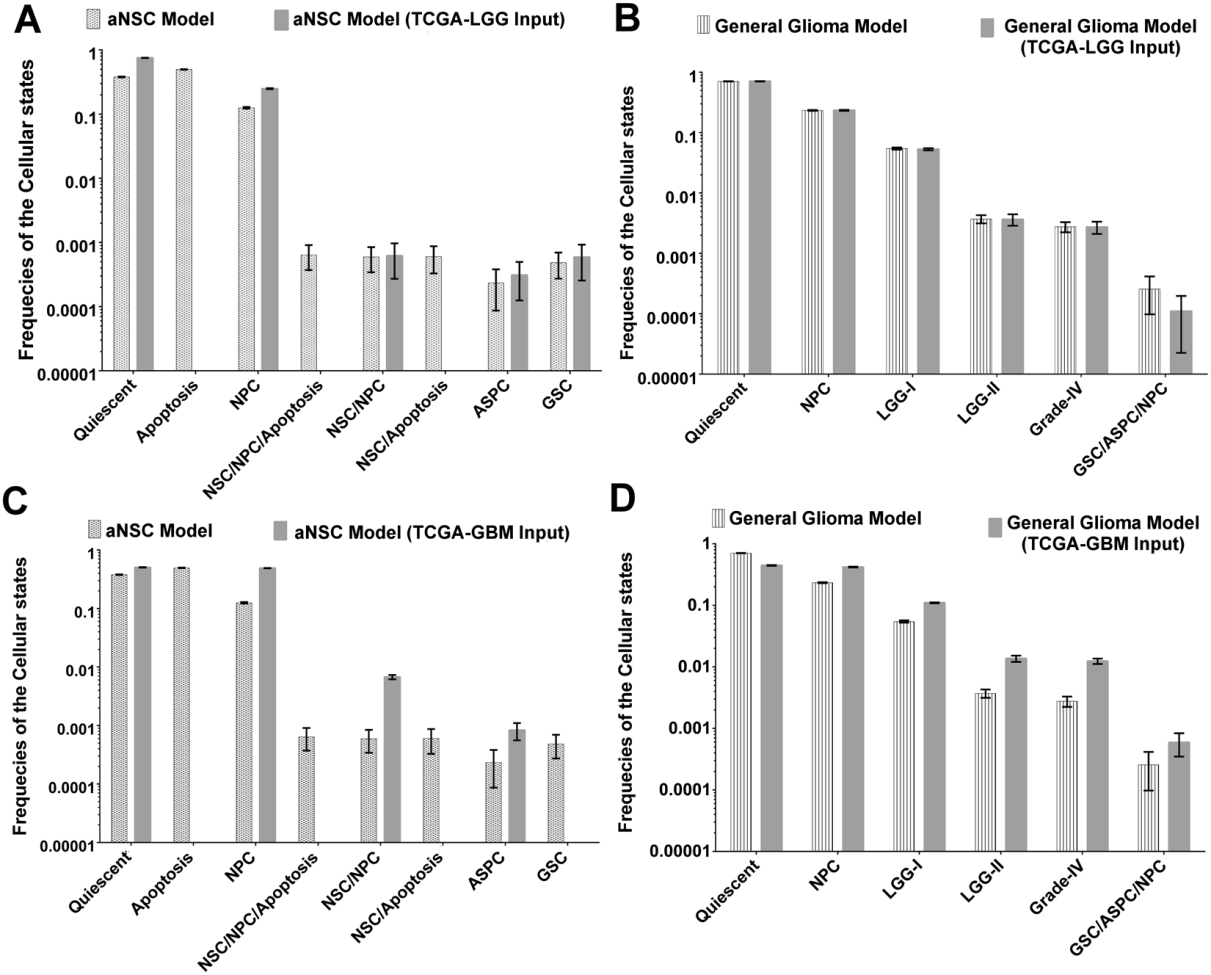
Comparative statistics of the normalized frequency distributions observed for different cellular states in master aNSC and general glioma models with and without the inputs of TCGA-LGG and TCGA-GBM RNA-Seq expression data. (**A**) A significant difference (Chi-Square goodness-of-fit test, P-Value < 0.001) is observed in the normalized frequency distributions while comparing the effect of TCGA-LGG expression profile in aNSC model simulation. Cellular states such as Apoptosis and NSC Renewal/Apoptosis are found to be absent in the aNSC model with TCGA-LGG expression profile. Normalized frequencies of ASPC Differentiation and GSC Renewal are also slightly increased in this scenario as compared to the master aNSC model simulation. (**B**) The normalized frequency distribution of the cellular states of general glioma model is well fitted with the simulation outcomes of general glioma model with transcriptomics profile of TCGA-LGG patient cohort. Similar to these analyses, mRNA expressions profile of the TCGA-GBM patient cohort is also given as inputs in (**C**) aNSC and (**D**) general glioma models. In both the scenarios, significant differences are observed between the expected and observed (outcomes from TCGA-GBM) normalized frequency distributions of the cellular states. These results prove that the developed aNSC and general glioma models are capable of differentiating the mRNA expression profiles of LGG and GBM patient cohorts correctly.

The transcriptomic profile of the transcripts of 9 out of 53 input molecules was found (Supplementary Table 6) in the differential expression analyses performed on the RNA-Seq data extracted from the cohort of P53 mutated, low-grade glioma (TCGA-LGG) tumor samples available in TCGA data portal. Using this TCGA-LGG transcriptomics data as inputs in the master aNSC model, a new simulation is performed to calculate the normalized frequencies of the observed cellular states (see Supplementary Information). At first, the new values were compared with the normalized frequencies obtained for each cellular state in the master aNSC model. After that, Chi-square goodness-of-fit test was performed between the normalized frequencies observed for each cellular state in the new simulation (observed data) versus normalized frequencies observed for each cellular state in the master aNSC model (expected data). This statistical test showed significant differences between the expected and observed data, which in turn proved that the transcriptomics profile extracted from the TCGA-LGG cohort did not indicate the development of normal neurogenesis of adult NSCs (Fig. 5A). For example, due to the imposed induction of the proteins (such as P53 mutation, and increased activities of DLL1, DLL3, etc.) in the new model, cellular states “Apoptosis” and “NSC/NPC/Apoptosis” were not found in the attractor space. On the other hand, the normalized frequencies of the cellular states “ASPC differentiation” and “GSC renewal” were found in higher numbers in the TCGA-LGG transcriptomics data model of aNSC. Hence, it proved that the neural stem cells having transcriptomics profile of TCGA-LGG cohort were more inclined to the development of glioblastoma stem-like (GSC) and mutated astrocytes or tumor cells (ASPC).

Further, the present study was aimed to assess the same transcriptomics profile (TCGA-LGG) on the development of GBM tumor cells (Supplementary Table 6). It was hypothesized here that if the same profile was taken as input in the master general glioma model, then the outputs of the model simulation will be able to capture the developmental dynamics of the tumorigenesis of low-grade glioma starting from the mutated GSCs. It was observed that all the cellular states, which were observed in the master general glioma model, were also appeared in the attractor space of this new model simulation. Furthermore, Chi-square goodness-of-fit test found that the values of the observed normalized frequencies of each cellular state fitted well with the expected normalized frequencies of each cellular state of the master general glioma model (Fig. 5B). In this new simulation, the mean values of the phenotype predictor scores of the tumorigenic cellular states *viz*. LGG-I (mean 19.00, 95% CI [18.60 19.41]), LGG-II (mean 1.10, 95% CI [0.98 1.22]), and Grade-IV (mean 0.83, 95% CI [0.73 0.93]) were similar to the values observed in master general glioma model (Supplementary Table 4). Hence, from this retrospective analysis, it was concluded that the transcriptomics profile of TCGA-LGG tumor samples was perfectly matching with the developed master general glioma model and therefore, this model could be used for further testing of other unknown transcriptomics profile to predict the existence of low-grade glioma. This new simulation was also able to indicate the early risk of the development of low-grade GBM in an individual by analyzing the transcriptomics profile of that individual.

Another retrospective analysis was also performed by using the transcriptomic profile of the input molecules, which were differentially expressed in the P53 mutated TCGA-GBM tumor samples collected from the high-grade Glioblastoma patients’ cohort (Supplementary Table 5). Total 19 transcripts out of the total 53 transcripts of the input molecules were found significantly expressed in the high-grade GBM tumor samples with respect to the normal (control) solid tumor samples (Supplementary Table 6). This transcriptomics profile was then provided as inputs in the master aNSC model to assess whether the differentially expressed genes/proteins of the transcripts of the input molecules were able to trigger normal neuronal cell development or not. It was observed that the transcriptomics profile while taken as inputs in the aNSC model were able to shift the NSCs developmental dynamics from the stem cell renewal process to the cell differentiation processes (i.e., NPC and ASPC). The normalized frequencies of cellular states observed in this new simulation were not found consistent with the cellular states observed in the master aNSC model (Chi-square goodness-of-fit test) (Fig. 5C). It was observed that the cellular state ASPC differentiation had a higher normalized frequency in the attractor distribution in the new simulation, which signified that the transcriptomics profile observed in TCGA-GBM sample cohort were biased towards the development of astrocytes cells in the neurogenic niche. These astrocytes cells had a higher number of mutations, abundant expressions of Notch and JAK/STAT pathway molecules with rapid proliferation rate, which in turn helped these cells to differentiate and transform into tumorigenic states. Differentiation of NPC was also observed in higher numbers, which indicated that the tumorigenic niche also contained matured (mutated) neuron-like cells. Cellular state Apoptosis was also absent in the new simulation, which was an indicator of the development of tumor cells. Overall, this study depicted that the transcriptomics profile of TCGA-GBM cohort was not helpful to the normal development of NSCs and could trigger tumorigenesis in future time-course.

To check the probabilities for developing high-grade GBM cells from the mutated GSCs a new simulation was again performed on this transcriptomics profile (extracted from TCGA-GBM cohort) by considering it as an input in the master general glioma model (see Supplementary Information). The simulation outcomes showed that although there was no other cellular state appeared in the simulation as compared to the master general glioma model, but the normalized frequencies of the cellular states were not consistent (Chi-square goodness-of-fit test, rejecting the null hypothesis at 99.99% significance level). It was found that the tumorigenic cells *viz*. LGG-I, LGG-II, Grade-IV and GSC/ASPC/NPC were significantly increased in the new simulation (Fig. 5D). Quiescent cells were significantly reduced, and simultaneously the differentiated NPC cells were increased. These results proved that the transcriptomics profile of TCGA-GBM tumor samples cohort could trigger stem cell differentiation as well as tumorigenesis. The inconsistency appeared between the normalized frequency values were due to the higher number of differentiation of neuronal and tumorigenic cells in the new model simulation.

The reasons behind the inconsistency of the normalized frequencies of cellular states were further analyzed by assessing the phenotype predictor scores. It was observed that the phenotype predictor scores of the tumorigenic states *viz*. LGG-I, LGG-II, GSC/ASPC/NPC and most importantly the Grade-IV tumor cells were significantly increased in the new simulation as compared to the scores observed in the master general glioma model (Supplementary Table 4). For example, the mean phenotype predictor score of Grade-IV tumor cells was calculated in the new simulation was 3.22 (95% CI [3.05 3.39]), which was approximately 3 times greater than the mean score (mean 0.970, 95% CI [0.873 1.066]) of Grade-IV cells observed in master general glioma model. The mean scores of other two tumorigenic cellular states LGG-I and LGG-II were 32.72 (95% CI [32.46 32.97]) and 3.51 (95% CI [3.30 3.71]) respectively, which were also slightly increased in the new simulation as compared to the master general glioma model simulation. On the other hand, the mean phenotype predictor score 133.04 (95% CI [132.21 133.87]) of “Quiescent” state in the new simulation was drastically reduced as compared to the mean score 301.827 (95% CI [300.945 302.709]) observed in the master general glioma model. Hence, this result indicated that the transcriptomics profile of TCGA-GBM tumor sample cohort was not only triggering the differentiation of mutated GSCs but also lead to the development of Grade-IV (high-grade) GBM cells. Therefore, it was concluded that the master general glioma model could predict the risk of the development of high-grade GBM cells, and simultaneously, the prospective study of the determination of the probable tumor grades could be performed by using its transcriptomics profile.

### A case study of screening and ranking of potential drug targets in high-grade GBM cells

Potential drug targets screening and its ranking by assessing their ability to suppress the Grade-IV tumorigenic cellular state were performed on the developed Grade-IV (high-grade) GBM model. The objective of this study was to check the efficacy of an individual or a combination of protein(s) to suppress the Grade-IV GBM tumor cells in the tumorigenic niche. A detailed description of the methodologies used for the drug target screening is discussed in Supplementary Information. The significant correlation and delay between the temporal dynamics of the Grade-IV cellular state observed in the Grade-IV GBM model (i.e., target signal) with the intermediate signaling molecules (i.e., query signal) was calculated in their corresponding normalized frequency domains (Supplementary Table 7). The critical proteins identified from this analysis (Supplementary Table 7) were targeted individually or in combinations in the high-grade GBM model, and the normalized frequencies of the LGG-I, LGG-II, and Grade-IV cellular states were compared with the master Grade-IV GBM model simulation results (Supplementary Fig. 9). It was observed that inhibition of STAT3 protein strongly affected the development of all grades of Glioblastoma tumor cells. Besides, increased activity of MASH1 (or NGN1) protein (TC4) or its higher expression with the inhibition of PI3K protein as a combination (TC5) were able to suppress the LGG-I glioblastoma cells completely but were unable to suppress LGG-II and Grade-IV cells significantly. On the other hand, inhibitions of PI3K/AKT and HIF1A simultaneously (TC6) was necessary to suppress the LGG-II tumor cells, but not the LGG-I and Grade-IV cells in the tumorigenic niche (Supplementary Fig. 9). Significant partial suppression of Grade-IV tumor cells was also observed while activating NGN1 (or MASH1) protein in the Grade-IV tumor cells. Therefore, this data suggests that the treatments that activate MASH1 may be useful in anti-GBM therapy as the simulation outcomes depicted the possible suppression mechanisms of both LGG-I and Grade-IV tumor cells in the simulated therapeutic conditions. However, it should be noted that LGG-II tumor cells, which were mostly unaffected by this new in-silico treatment strategy, have the chance to recur as Grade-IV GBM tumor cells in the future.

Hence, from this analysis, it was possible to rank the target proteins, which had a significant effect on perturbing the LGG-I, LGG-II, and Grade-IV tumor cells in the high-grade GBM tumor niche. The ranking for each protein to target the tumorigenic cells is provided in Supplementary Table 8. In the previous experiments, inhibition of STAT3 protein is found highly effective to suppress Glioblastoma tumor cells^29,30^. However, the inhibition of STAT3 protein in cancer therapy also triggers various toxic side effects as this protein is involved in many other signaling pathways responsible for the development of normal neural cell lineages^31^. It has been observed that targeting MASH1 by over-expressing this protein in the tumor cells is sufficient for the treatment of high-grade GBM^32^. MASH1 is a neurogenic gene and the over-expression of this protein will trigger the differentiation process of neurons from the GSCs present in the tumor niche and would be helpful to redirect the mutated stem cells from further astrocytes differentiation process^33^. Applications of such targeted, cell-based differentiation therapy would be very much practical as it has a less chance of developing tumorigenic (or differentiated and mutated astrocytoma) cells after therapy and thus may reduce the probability of tumor relapse.

## Discussion

Population based study on Glioblastoma tumor by TCGA consortium has demonstrated the existence of four different tumor subtypes within the high-grade GBM patient cohort^34^. Later, Patel *et al*. have explored the possible cell states with diverse transcriptional programs within the same tumor cells using single-cell RNA-Seq analysis and provided a substantial evidence of the existence of intra-tumor heterogeneity in GBM^35^. These studies have triggered several intriguing questions related to the mechanisms of the development of tumor sub-types and the heterogeneous groups of tumor cells within same grade of GBM tumor. Also, the molecular signatures between adult NSCs and tumorigenic GSCs are observed to be similar in various aspects, and it is understood that both of these cells share a common origin of evolution during development^3^. However, the dynamic regulations behind the emergence of tumorigenic lineages in the SVZ of the human brain are unknown hitherto.

In this work, a novel approach is proposed to understand the molecular processes involved in the regulations of phenotypic plasticity and renewal of aNSCs in the neurogenic niche. This analysis also helped to understand the molecular mechanisms and differential hierarchy established by aNSC to establish the lineage of GSCs during its development under the exposure of a variety of input signals. The role of juxtacrine Notch signaling and its cross-talk reaction mechanisms are considered for this purpose as the involvement of Notch target genes *HES1*, *HES5*, etc. are proven to be strongly correlated with the regulation of neurogenesis as well as gliogenesis in brain tissue^36^. While studying the normal neurogenic and tumorigenic niches, the simulation studies performed on the aNSC, GSC, general glioma and Grade-IV GBM models were also able to show the emergence of different cell lineages (Supplementary Fig. 1) which are similar to the observations found in the previous experiments^37,38^. The performance of these developed models was measured by calculating the maximum number of appropriate cell types appeared in a simulation with highest normalized frequency distributions (and Shannon entropy scores)^39^. Further, by introducing a novel scoring parameter “Activity Ratio (AR)” score, the active regulatory motifs which played the critical roles to maintain the dynamic balance of this entire developmental process of aNSCs were also identified (Supplementary Fig. 4).

It was observed that the three interconnected gene regulatory networks and the P53 dependent apoptotic network, which were responsible for the transcription of marker genes involved in stem cell maintenance, apoptosis, and the differentiation of neurons and astrocytes, were greatly influenced by the activities of Notch signaling network during GBM development (Supplementary Fig. 5). On the other hand, Notch signaling network was stimulated by several extrinsic (such as ligands) and intrinsic (e.g., kinase, transcription co-activator, repressors, crosstalk reactions, etc.) factors, which profoundly differed based on the microenvironment of the cancer cells as well as its genomic/proteomic profiles. However, it was observed that Notch pathway alone could not regulate or initiate all the cellular states. For example, to trigger the gliogenesis process, Notch target proteins HES1/5 should be highly abundant in the cells to stimulate the kinase activity of the JAK2 protein and the successive phosphorylation of the transcription factor STAT3 protein. STAT3 is the transcription factor of the gene responsible for the expression of GFAP protein, which is a known marker of astrocytes cells^40^. Hence, from this motif identification analyses, it was clearly understood that how the intracellular Notch signaling network performed multiple developmental processes in adult NSCs and governed the neurogenesis, gliogenesis as well as tumorigenesis. This mechanistic understanding further helped to assess the effects of the perturbations within Notch signaling network through which normal aNSCs develop proliferating GSCs and further trigger the development of different grades of Glioblastoma tumor in the human brain.

**Figure 6:**
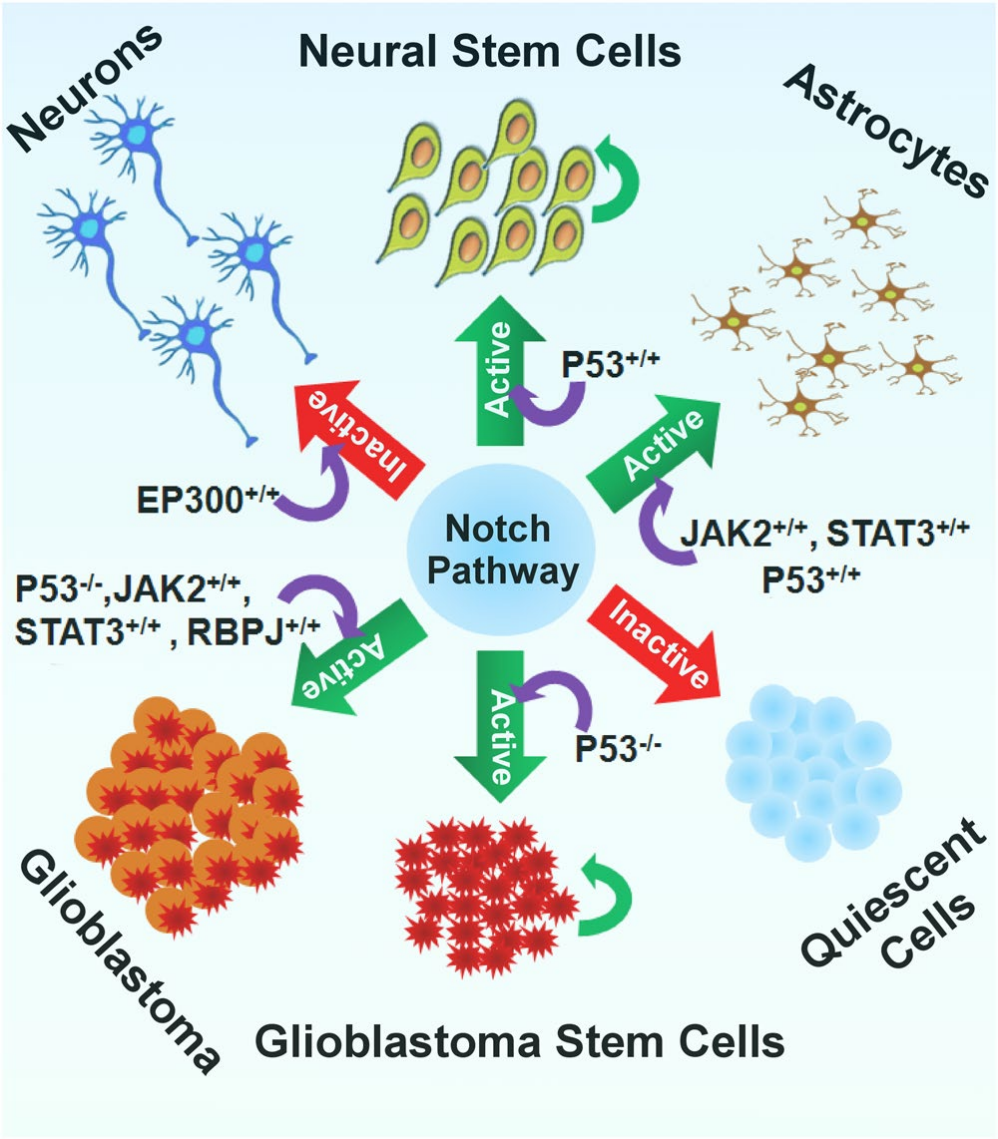
The rheostat model proposed for Notch signaling network. This model shows the mechanistic regulations of core Notch pathway during the evolutions of different sub-types of normal and tumorigenic cells from adult neural cells.

Finally, it was observed that the core Notch pathway is primarily used in the adult NSCs as a rheostat, which can be tuned by regulating (i.e., mutation, activation, inhibition, etc.) various proteins to achieve desired phenotypic outputs (Fig. 6). Simulation outcomes showed that if the activities of the core component molecules of Notch pathway (e.g., Notch receptors and ligands, γ-secretase enzyme complex, MAML, RBPJ or CSL, HES/HEY, etc.) were very less, then the stem cells would stay at its quiescent state (qNSCs). On the other hand, the cells would lose its stemness or quiescent properties, if EP300 protein had increased activity in the aNSCs/aNSCs. In this case, the stem cells will switch to the differentiation process and develop into matured neurons. The neural stem cells would retain its self-renewing state if the core components of this pathway were abundant in the cells to produce HES/HEY, CYCLIN-D1, etc. proteins periodically and simultaneously possessed the wild-type active P53 protein. Furthermore, if the P53 protein was mutated but Notch pathway was active, then the stem cell dynamics would alter and redirect towards the development of self-renewing Glioblastoma stem cells (GSCs). It was also observed that in the normal, wild-type adult NSCs (with wild-type P53), if the targets proteins (i.e., HES1, HES5) of active notch pathway interact with the JAK2/STAT3 protein, then the developmental dynamics of adult NSCs would redirect towards the development of gliogenesis (i.e., astrocytes) process. It was also observed that the same gliogenesis process could be malfunctioned and lead to the development of Glioblastoma tumor formation if the differentiating cells had mutant P53 protein.

The master general glioma model, developed for simulating the tumorigenesis process, was also able to predict the developmental routes through which the intra-tumor heterogeneities of tumor cells of different grades emerged in the tumorigenic niche of Glioblastoma (Fig. 3, Supplementary Figs 6 and 7). The critical time points, at which the different grades (sub-types) of GBM tumors and the molecular event of intra-tumor heterogeneity occur, were also possible to extract for analyzing the differences in the molecular profiles of the tumor cells at these critical junctions. Moreover, using the newly introduced phenotype prediction score, it was possible to quantify the probabilities of occurrences of different cellular states as well as tumor grades during the time of tumorigenesis. This scoring technique correctly predicted the bias towards the development of low-grade astrocytoma from mutated aNSCs/GSCs or the bias towards the development of high-grade GBM cells from low-grade GBM. To validate the existence of such bias, a case study was performed considering the TCGA RNA-Seq data from low and high-grade GBM tumor samples cohort as the inputs for master aNSC and general glioma models, respectively. The simulation results correctly predicted the higher probabilities of low grade and high-grade (Grade-IV) GBM tumor cells in the general glioma model (Fig. 5). After this prediction study, the proposed Grade-IV GBM model was used for screening and ranking of potential drug targets for the suppression of the growth of Grade-IV tumor cells. A novel methodology was developed for this purpose in which Fast Fourier Transformation (FFT) technique was used to find out the correlation and lag between the temporal dynamics of Grade-IV tumor state with the temporal expression pattern observed for each pathway molecule. It was observed that the protein molecules correlating higher than 0.6 and lag less than or equal to 3 time-steps with the Grade-IV tumor cell were the suitable drug targets to perturb the Grade-IV tumorigenic signal. In silico perturbations were performed on the Grade-IV GBM model to explore the effects of targeting these identified drug targets individually or in combinations. The drug target (such as MASH1), which showed huge reductions of the normalized frequencies of LGG-I, LGG-II, and most importantly Grade-IV tumor cells, but did not affect the growth of non-tumorigenic cells (NPCs), were considered as the highly preferable target(s) (Supplementary Table 8).

To deploy these entire proposed mechanistic models of neurogenesis, gliogenesis and tumorigenesis processes in the field of pathological and prognostic studies of GBM tumor cells, the entire decision-making protocol is provided in a flow chart for its better understanding in the Supplementary Information (Supplementary Fig. 10). This protocol can be used in personalized, target-based GBM tumor therapy, in which the patient’s omics data (e.g., differentially expressed transcripts, proteins, metabolites, etc.) will be considered as inputs in the proposed model and the chances or risk of developing GBM tumor will be assessed, followed by drug targets screening and identification process. Moreover, the novel scoring techniques (i.e., AR score, Phenotype predictor score) are found to be very much useful for the prediction of the emergence of different tumor grades, its heterogeneity and to find out the potential drug targets for personalized target-based cancer therapy. Based on these methodologies and the protocols, the computational framework proposed in this work can provide better solutions in onco-pathological research.

This work has provided new concepts for better understanding of the biological signal transduction mechanisms in normal and diseased scenarios. The overall methodologies provided here can be applicable to multiple biological processes (such as stem cell development, neurogenesis, tumorigenesis, etc.) and can be implicated for a diverse range of investigations in future. If integrated with robust experimental data, this work would be helpful to substantially advance the understanding of the mechanisms underlying cellular responses to external or internal cues, and refine the current views of the signaling processes. Moreover, the proposed generic models explain the development of de novo tumors and its grades, but these can also show the development of higher grades including Grade III and secondary Grade IV tumor from low grade gliomas (Grade II). Also, one of the limitations of the current modeling strategies proposed in this work is its inability to implement the “clonal evolution model” for simulating the development of tumor sub-clones and intra-tumor heterogeneity. In the future work, this theory could also be implemented by introducing the stochastic mutations on the genes/proteins of Notch and its cross-talk pathways during execution of the simulations of tumor cell models.

## Materials and Methods

### Adult neural stem cell model development and simulation

The chemical reactions of Notch and its cross talks proteins (JAK2/STAT3, HIF1A, P53, etc.) were compiled from the previously published model of Notch pathway, which was further modified to simulate the developmental dynamics of adult neural stem cells (aNSCs) (Supplementary Tables 1 and 2)^41^. The reactions were translated into a total of 69 logical equations with 122 logical nodes (Supplementary Tables 1 and 2). The logical nodes consist of 53 input molecules (i.e., no upstream regulators), 39 intermediate (connected with upstream and downstream) molecules, 25 target (with no downstream) proteins, and 5 phenotypes (Supplementary Table 2). To stimulate the Notch signaling, receptor proteins NOTCH 1/2/3/4 (intermediate proteins) were considered as ON (i.e., increased abundance/activity), and the rest of the molecules (except inputs) were kept as OFF (i.e., decreased abundance) at the initial state (at time, t = 0). At this stage, the constructed model had no mutation (i.e., no time invariable molecule, hence total mutation, μ = 0) and was simulated for the normal development of adult NSCs (denoted as aNSC model). The phenotypes were mapped with different marker proteins described in Supplementary Table 3.

The simulation outcomes of the model were represented by analyzing the state transition graph (STG), in which the expression vector of all the nodes (i.e., molecules) considered in the logical model simulation at a particular time (*t*) was considered as a “state (*S*_*t*_)”^42^. The state transitions path from *S*_0_–>*S*_1_–>*S*_2_–>…–>…–>*S*_*T*_ was considered as state transition graph (STG).The transitions of the states were continued until it reached the steady-state levels at time *T*. The state *S*_*T*_ was considered as stable focus or singleton or fixed-point attractor state if *S*_*T−*1_ = *S*_*T*_ = *S*_*T*+*1*_. On the other hand, if *S*_*T*_ = *S*_*T+P*_, then the state *S*_*T*_ was considered as cyclic state with periodicity *P* (see Supplementary Information). In logical simulation, if there are *N* numbers of input nodes present in the developed model, then there exist 2^*N*^ numbers $\{{S}_{0}^{1},{S}_{0}^{2},{S}_{0}^{3},\,\ldots ,\,{S}_{0}^{N}\}$ of maximum possible initial states, which is also called as initial state space. Starting from this initial state space, the logical model of any biochemical pathway (or any system of equations) can evolve dynamically until it reaches the steady-states or attractor states. There could be *N* numbers of attractor states possible to be observed in STG, but most often the STG of a system converges to a small number of attractor states $\{{S}_{T}^{1},{S}_{T}^{2},{S}_{T}^{3},\,\ldots ,\,{S}_{T}^{A}\}$, where *A* ≤ *T*. The set of all possible attractor states observed in the logical simulation of a system of equations is known as the attractor space of the system.

It should be noted that the attractors in the attractor space obtained in the model simulations are the expression profiles of the intermediate and output proteins considered in the developed models (Supplementary Table 2). In the attractor space, the expression profiles of the marker proteins were extracted from each attractor states (Supplementary Table 3). Followed this, the phenotypic properties of the attractors were assigned based on the expressions (binary 1 or 0) of the extracted marker proteins for each attractor states. It should be noted that depending on the expressions of the phenotypic marker proteins, multiple phenotypes can be assigned to a single attractor state. For example, the markers of aNSC and NPC can be expressed in a single attractor state, and therefore the attractor state can be further defined as a cellular state depicting the bi-potent state of aNSC renewal and NPC differentiation.

### Normalized frequency, Shannon entropy, and activity ratio score

The normalized frequency of a cellular state in a particular simulation batch is the ratio of its observed normalized frequency to a total number of random initial conditions. Shannon entropy measures the total information content of a simulation batch by measuring the summation of the negative logarithms of the probability mass functions of all the observed cellular states. The “Activity Ratio (AR)” score is calculated for any input protein/node concerning a particular cellular state of the logical model simulation. It is measured by calculating the total number times the node was active (i.e., increased abundance) or inactive (i.e., decreased abundance) within all the random input sequences, which triggered that particular cellular state in the attractor space (see Supplementary Information).

### Calculation of phenotype cost function

This function is calculated by measuring the ratio of the average Hamming distance traversed by all the molecules while reaching the particular phenotype/cellular state to the normalized frequency of the phenotypes (Supplementary Information).

### RNA-Seq data analyses and differential mRNA expression study

High-throughput, raw RNA-Seq counts data provided by The Cancer Genome Atlas (TCGA) were downloaded from high (TCGA-GBM) and low grade (TCGA-LGG) tumor sample cohorts with a P53 mutation on 27th April 2017 from GDC Data portal (https://portal.gdc.cancer.gov/). The overall statistics of the downloaded RNA-Seq data, which includes 511 and 617 patients from TCGA-LGG and TCGA-GBM cohorts, are provided in Supplementary Table 5. The raw HTSeq counts data were further processed and analyzed for differential gene expression (DEG) analysis to find out the transcripts, which were significantly expressed in the LGG and GBM tumor cells in comparison to the normal, solid tumor cells (see Supplementary Information).

### Drug targets screening and ranking

The molecules, which were significantly positive and negative inducer of high-grade (Grade-IV) cellular state, were extracted through FFT analyses performed on the time course activity sequences of all the intermediate proteins of Notch signaling network. The correlation and delay of the trajectories of the temporal expression dynamics of all the molecules were measured with respect to the reference trajectory of Grade-IV cellular state observed in the STG of “Grade-IV GBM model” simulation study (see Supplementary Information).

## Supplementary information

Supplementary Information

## Data Availability

The model files required to reproduce the simulation results are provided in the supplementary information.

## References

[1] Hu YY (2011). Notch signaling contributes to the maintenance of both normal neural stem cells and patient-derived glioma stem cells. BMC Cancer.

[2] Patel AP (2014). Single-cell RNA-seq highlights intratumoral heterogeneity in primary glioblastoma. Science.

[3] Kawamura Yoichiro, Takouda Jun, Yoshimoto Koji, Nakashima Kinichi (2018). New aspects of glioblastoma multiforme revealed by similarities between neural and glioblastoma stem cells. Cell Biology and Toxicology.

[4] Stockhausen MT, Kristoffersen K, Poulsen HS (2010). The functional role of Notch signaling in human gliomas. Neuro Oncol.

[5] Zhang X (2012). Notch1 promotes glioma cell migration and invasion by stimulating β‐catenin and NF‐κB signaling via AKT activation. Cancer Sci..

[6] Wang L (2008). Gamma-secretase represents a therapeutic target for the treatment of invasive glioma mediated by the p75 neurotrophin receptor. PLoS Biol..

[7] Lee J (2008). Epigenetic-mediated dysfunction of the bone morphogenetic protein pathway inhibits differentiation of glioblastoma-initiating cells. Cancer Cell.

[8] Sunayama J (2010). Crosstalk between the PI3K/mTOR and MEK/ERK pathways involved in the maintenance of self renewal and tumorigenicity of glioblastoma stem like cells. Stem Cells.

[9] Hede SM, Nazarenko I, Nister M, Lindstrom MS (2011). Novel Perspectives on p53 Function in Neural Stem Cells and Brain Tumors. J Oncol.

[10] Prasetyanti PR, Medema JP (2017). Intra-tumor heterogeneity from a cancer stem cell perspective. Molecular cancer.

[11] Sottoriva A (2013). Intratumor heterogeneity in human glioblastoma reflects cancer evolutionary dynamics. Proc Natl Acad Sci USA.

[12] Shih AH, Holland EC (2006). Notch signaling enhances nestin expression in gliomas. Neoplasia.

[13] Prestegarden L (2010). Glioma cell populations grouped by different cell type markers drive brain tumor growth. Cancer Res..

[14] Shibata M, Shen MM (2013). The roots of cancer: stem cells and the basis for tumor heterogeneity. BioEssays: news and reviews in molecular, cellular and developmental biology.

[15] Brennan C (2009). Glioblastoma subclasses can be defined by activity among signal transduction pathways and associated genomic alterations. PLoS One.

[16] Esdar C, Milasta S, Maelicke A, Herget T (2001). Differentiation-associated apoptosis of neural stem cells is effected by Bcl-2 overexpression: impact on cell lineage determination. Eur. J. Cell Biol..

[17] Sandquist EJ, Sakaguchi DS (2019). Adult neural stem cell plasticity. Neural Regen Res.

[18] Balaganapathy P (2018). Interplay between Notch and p53 promotes neuronal cell death in ischemic stroke. J Cereb Blood Flow Metab.

[19] Liu L (2018). Impaired Notch Signaling Leads to a Decrease in p53 Activity and Mitotic Catastrophe in Aged Muscle Stem Cells. Cell Stem Cell.

[20] Yun J (2015). p53 Modulates Notch Signaling in MCF-7 Breast Cancer Cells by Associating With the Notch Transcriptional Complex Via MAML1. J Cell Physiol.

[21] Kamakura S (2004). Hes binding to STAT3 mediates crosstalk between Notch and JAK-STAT signalling. Nat. Cell Biol..

[22] Hong S, Song MR (2014). STAT3 but not STAT1 is required for astrocyte differentiation. PLoS One.

[23] Claus EB (2015). Survival and low-grade glioma: the emergence of genetic information. Neurosurg Focus.

[24] Armesilla-Diaz A (2009). p53 regulates the self-renewal and differentiation of neural precursors. Neuroscience.

[25] Fu S (2016). Aberrant Adult Neurogenesis in the Subventricular Zone-Rostral Migratory Stream-Olfactory Bulb System Following Subchronic Manganese Exposure. Toxicol. Sci..

[26] O’Brien ER, Howarth C, Sibson NR (2013). The role of astrocytes in CNS tumors: pre-clinical models and novel imaging approaches. Front Cell Neurosci.

[27] Cedilnik A, Kosmelj K, Blejec A (2004). The distribution of the ratio of jointly normal variables. Metodoloski zvezki.

[28] Willan, A. R. & O’Brien, B. J. Confidence intervals for cost-effectiveness ratios: an application of Fieller’s theorem. *Health Econ* **5**, 297–305, 10.1002/(SICI)1099-1050(199607)5:4<297::AID-HEC216>3.0.CO;2-T (1996). 10.1002/(SICI)1099-1050(199607)5:4<297::AID-HEC216>3.0.CO;2-T 8880166

[29] Birner P, Toumangelova-Uzeir K, Natchev S, Guentchev M (2010). STAT3 tyrosine phosphorylation influences survival in glioblastoma. J Neurooncol.

[30] Kim JE, Patel M, Ruzevick J, Jackson CM, Lim M (2014). STAT3 Activation in Glioblastoma: Biochemical and Therapeutic Implications. Cancers (Basel).

[31] Chiba T, Mack L, Delis N, Brill B, Groner B (2012). Stat3 inhibition in neural lineage cells. Horm Mol Biol Clin Investig.

[32] Park NI (2017). ASCL1 Reorganizes Chromatin to Direct Neuronal Fate and Suppress Tumorigenicity of Glioblastoma Stem Cells. Cell Stem Cell.

[33] Ball DW (2004). Achaete-scute homolog-1 and Notch in lung neuroendocrine development and cancer. Cancer Lett..

[34] Verhaak RG (2010). Integrated genomic analysis identifies clinically relevant subtypes of glioblastoma characterized by abnormalities in PDGFRA, IDH1, EGFR, and NF1. Cancer Cell.

[35] Patel VN (2013). Network signatures of survival in glioblastoma multiforme. PLoS Comput Biol.

[36] Bansod S, Kageyama R, Ohtsuka T (2017). Hes5 regulates the transition timing of neurogenesis and gliogenesis in mammalian neocortical development. Development.

[37] Basak O, Giachino C, Fiorini E, Macdonald HR, Taylor V (2012). Neurogenic subventricular zone stem/progenitor cells are Notch1-dependent in their active but not quiescent state. J. Neurosci..

[38] de Almeida Sassi F, Lunardi Brunetto A, Schwartsmann G, Roesler R, Abujamra AL (2012). Glioma revisited: from neurogenesis and cancer stem cells to the epigenetic regulation of the niche. J Oncol.

[39] de Oliveira ME, Neto LM (2016). Directional entropy based model for diffusivity-driven tumor growth. Math Biosci Eng.

[40] Ge W (2002). Notch signaling promotes astrogliogenesis via direct CSL-mediated glial gene activation. J. Neurosci. Res..

[41] Chowdhury S, Sarkar RRD (2013). Targets and Biomarker Identification from Computational Study of Human Notch Signaling Pathway. Clin Exp Pharmacol.

[42] Gates AJ, Rocha LM (2016). Control of complex networks requires both structure and dynamics. Sci Rep.

