# Supplementary material for: Exploring Notch Pathway to Elucidate Phenotypic Plasticity and Intra-tumor Heterogeneity in Gliomas
Source: Sci Rep. 2019 Jul 1;9:9488. doi: 10.1038/s41598-019-45892-8 (PMC6602950; doi:10.1038/s41598-019-45892-8)
Supplement: Supplementary file 1 — Supplementary Information [file 41598_2019_45892_MOESM1_ESM.pdf]

# Supplementary Information

# Exploring Notch Pathway to Elucidate Phenotypic Plasticity and Intra-tumor Heterogeneity in Gliomas

Saikat Chowdhury

1. Chemical Engineering and Process Development Division,
CSIR-National Chemical Laboratory,
Pune, Maharashtra, 411008, India

Email: *sr.chowdhury@ncl.res.in*

&

2. Academy of Scientific & Innovative Research (AcSIR),
CSIR-NCL Campus, Pune, India

Ram Rup Sarkar*

1. Chemical Engineering and Process Development Division,
CSIR-National Chemical Laboratory,
Pune, Maharashtra, 411008, India

Email: *rr.sarkar@ncl.res.in*

&

2. Academy of Scientific & Innovative Research (AcSIR),
CSIR-NCL Campus, Pune, India

* Corresponding author: Tel: +91 2590 3040; Fax: +91-20-2590 2621;

# Table of Contents

**1. Materials and Methods** .......... **1**

1.1 Construction of Logical Model. .......... 1

1.2 Marker proteins of aNSC, GSC and General glioma models .......... 11

1.3 Determining the phenotypes and cellular states .......... 12

1.4 Calculation of normalized frequencies of cellular states, Shannon entropy and Activity Ratio (AR) scores .......... 13

1.5 Calculation of phenotype cost function .......... 17

**2 Model constructions and validation** .......... **20**

2.1 Exploration of maximum number of attractor states using Shannon entropy in aNSC model. .......... 20

2.2 Developmental model captures the emergence of cellular heterogeneity in the aNSCs niche. .......... 20

2.3 aNSCs are highly biased towards the development of quiescent, neural progenitor and apoptotic states .......... 21

2.4 Application of Activity Ratio scores for the extraction of driver proteins at different cellular states .......... 22

2.5 P53 mutation does not affect the differentiation potential of Glioblastoma stem cells .......... 23

2.6 The nexus of YY1 transcription network and core Notch signaling induces tumorigenesis in GSCs .......... 23

**3 Data Analyses** .......... **25**

3.1 Temporal variations of marker proteins in aNSC, GSC and General glioma models .......... 25

3.2 Network motifs of Notch pathway in the regulation of different cellular states .......... 30

3.3 State transition graph of General glioma model simulation .......... 32

3.4 Protein activity pattern observed in different attractor states corresponding to Grade-IV cells .......... 33

3.5 Distributions of the phenotype predictor scores observed for all type of cellular states..............33

**4 Case Studies.........................................................................................................................35**

4.1 Case study using TCGA-LGG & TCGA-GBM RNASeq samples data......................................35

4.1.1. Selection of patient cohorts and preparation of RNASeq sample data sets ..........................35

4.1.2. Differential expression analyses of mRNA molecules ........................................................36

4.2 Logical simulations using TCGA-LGG and TCGA-GBM transcriptomics data:.........................37

4.2.1 Analyses of TCGA-LGG patient cohort ..............................................................................37

4.2.2 Analyses of TCGA-GBM patient cohort .............................................................................38

**5 Methodologies used for drug target screening ..................................................................39**

5.1 Flow-chart of the decision making protocols used in cancer risk prediction, tumor grade detection and drug target screening ..........................................................................................44

**6 References:.........................................................................................................................45**

# 1. Materials and Methods

### *1.1 Construction of Logical Model.*

To understand the neurogenesis of adult neural stem cells (aNSCs) in sub-ventricular zone (SVZ) of human brain, Notch signaling pathway and its cross-talk reactions with other pathway molecules (i.e. JAK2/STAT3, HIF1A, P53, RAS, PI3K/AKT, WDR12, and JIP1) are considered to construct a comprehensive logical dynamic model (Supplementary Table 1). The preliminary data for constructing the core pathway model is taken from the previously published work of the logical dynamic model of Notch pathway [1]. Based on this pathway data, the entire model is then modified and restructured to simulate the developmental dynamics of adult neural stem cells (aNSCs) and the mutated Glioblastoma stem cells (GSCs). The overall reaction mechanisms of this pathway are then translated into logical equations, using universal logic gates *AND*, *OR* and *NOT*. The methodologies especially used for developing the logical equations of biochemical pathways are mainly adopted from the previously published works on the theoretical and experimental studies of the logical modeling and dynamic analyses of gene regulatory and cell signaling networks [1-5]. The logical rules defining the dependencies of the pathway molecules are derived from related literatures and the chemical regulations of the constructed signaling network.

The intracellular signaling cascade of Notch and its cross talk reactions can drive the phenotypic behaviors of aNSCs/qNSCs at various directions and thus create a distribution of multiple, distinct cell types (e.g. neurons, astrocyte, oligodendrocyte etc.) in the neurogenic niche of SVZ [6,7]. It is observed that to reach a particular cellular phenotype, a specific set of molecules (markers) in the signaling cascade get activated, which forms a functional module or a sub-network and helps to express a class of marker proteins responsible for a specific cell type [7]. The dynamics of the pathway molecules are updated synchronously and the following rules (***Eq.1-2***) were considered while constructing the dynamic Boolean model of Notch and its cross talks reactions considered in this work. The initial states (1/ON or 0/OFF) of the input nodes are chosen randomly by generating random number for each input node from uniform distribution $\left(Unif(0,1)\right)$ in the range of 0 to 1 and setting the cutoff at 0.5 (***Eq. 2***).

$$X_i(t+1) = X_i(0) \;\; iff \; \forall x_i \in Input\,Nodes \tag{1}$$

$$= f_i(X_j^{\,1}(t), X_j^{\,2}(t), X_j^{\,3}(t), \ldots, X_j^{\,k_i}(t)) \;\; iff \;\; \forall x_i \in Intermediate\,and\,Output\,Nodes$$

$$With, initial\,conditions: X_i(0) = 1 \;\; iff\; Unif(0,1) \geq 0.5 \tag{2}$$

$$= 0 \;\; otherwise$$

$$Where, k_i = Total\,number\,of\;Input\,Nodes$$

The pathway consists of 117 molecules out of which 53 are input molecules (Supplementary Table 2). Here, input molecules refer the molecules that do not possess any predecessor molecules (i.e. no upstream regulators) and during the signaling event they can be at either up-regulated (ON) or down-regulated (OFF) state (***Eq.1***). Hence, the possible highest numbers of expression patterns of these input molecules are $2^{53}$ (~$9.0071993 \times 10^{15}$). In presence of such enormous number of possibilities, mainly occurred due to the variations in the expressions of several extrinsic and intrinsic molecules, it is indeed obvious that adult and inactive NSCs (aNSCs) in the neurogenic niche of SVZ have to make right decision to opt any of the cellular states/phenotypes

either by proliferation and differentiation during the developmental process. The aNSCs can also choose to stop its cell division process (i.e. undergo cell cycle arrest or quiescent state) or undergo natural cell death process (i.e. apoptosis). Hence, it would be indeed an interesting work to trace the developmental dynamics and decision-making processes of aNSCs under the influence of such enormous input conditions through *in-silico* model simulations.

Hence, to capture such huge possibilities at equilibrium, a robust simulation technique to stimulate the developmental dynamics of aNSCs with all input states (~ 9.0071993 X 1015) will be required. However, in practical scenario, logical dynamic simulations by considering all the input conditions and finding all possible attractors at equilibrium state are not feasible in terms of required computational cost. Hence, to reduce the computational cost, a fraction (total 106) random, non-redundant initial input sequences are generated by using uniform random number distribution and subsequently assigned binary states ON (1) or OFF (0) to each input node of the reconstructed model (Eq. 2). The entire random input sequences (106) are then further divided into 100 separate simulation batches (10,000 random sequences) and simulations are performed for all the 100 batches. Boolean functions shown in Eq. 2 for each node/molecule are provided in Supplementary Table 1.

To capture different biological and realistic scenarios, the simulation of the model can be performed in the following three different ways depending on the availability of information of the input nodes.

a) The logical states (binary) of the input molecules (mentioned in supplementary table 2) could be completely randomized and used as initial values to start the simulation. In this case, we can explore the activities of all possible combinations of the input molecules on the development of aNSC and GSCs. The simulation strategy is more close to the situation of real biological scenarios in which the stem cells (normal or tumorigenic) are exposed into a variety of the combinations of input signals (intrinsic and extrinsic) in the tissue.

b) The expressions/activities (Up/Active or Down/Inactive) of the input molecules obtained from transcriptomics, proteomics, and metabolomics experiments could be used as initial values to start the simulation. In this case, the modeling strategy is more close to the context specific simulation of the individual biological cells.

c) In case, the expression/activity levels of the input molecules are partially known, our developed models could also be used for simulation. Here, the input molecules which have known expression/activity level will be kept fixed (or time invariant) during simulation, and rest of the inputs will be randomized. Using this strategy, one can develop a predictive model for simulating and predicting the joint outcomes of the activities of both sets of input molecules having fixed/significant as well as random/uncertain/insignificant levels of expressions/activities in the normal and tumor cells. We used this modeling strategy to observe the effects of the transcriptomics information obtained from the TCGA-LGG and TCGA-GBM cohorts on our developed models (See Data Analysis section).

## Supplementary Table 1. Master model of Notch signaling network

| |
|---|
| *AKT (t+1) = (PI3K(t) )* |
| *BAX (t+1) = (P53_P(t) )* |
| *BCL2 (t+1) = (NUC_NICD1(t) and COA(t) and CSL(t) and not COR(t) )* |
| *CCND1 (t+1) = (NICD_ACTIVE(t) and CSL(t) and not COR(t) and COA(t) )* |
| *CCND3 (t+1) = (NICD_ACTIVE(t) and CSL(t) and not COR(t) and COA(t) )* |
| *CD44 (t+1) = (NICD_ACTIVE(t) and CSL(t) and not COR(t) and COA(t) )* |
| *CDK2 (t+1) = (NICD_ACTIVE(t) )* |
| *MAML+P53_P (t+1) = (MAML(t) and P53_P(t) )* |
| *COA (t+1) = (EP300(t) and MAML(t) and HAT (t) and SKIP(t) )* |
| *COR (t+1) = (HDAC(t) and SAP30(t) and CIR(t) and SIN3A(t) and SMRT(t) )* |
| *FLIP (t+1) = (NUC_NICD1(t) and COA(t) and CSL(t) and not COR(t) )* |
| *GAMMA_SECRETASE (t+1) = (PRESENILIN1(t) and NICASTRIN(t) and APH1(t) and PEN2(t) ) or (NICASTRIN(t) and APH1(t) and PEN2(t) )* |
| *HES1_MRNA (t+1) = (NUC_NICD1(t) and SMAD3(t) and CSL(t) and not HES1(t) ) or (NICD_ACTIVE(t) and CSL(t) and not COR(t) and COA(t) and not MAML+P53_P(t) and not HES1(t) )* |
| *HES1 (t+1) = (HES1_MRNA(t) )* |
| *HES5_MRNA (t+1) = (NICD_ACTIVE(t) and CSL(t) and not COR(t) and COA(t) and not HES5(t) )* |
| *HES5 (t+1) = (HES5_MRNA(t) )* |
| *HES7 (t+1) = (NICD_ACTIVE(t) and CSL(t) and not COR(t) and COA(t) )* |
| *HEY1 (t+1) = (NICD_ACTIVE(t) and CSL(t) and not COR(t) and COA(t) )* |
| *HEY2 (t+1) = (NICD_ACTIVE(t) and CSL(t) and not COR(t) and COA(t) )* |
| *HEYL (t+1) = (NICD_ACTIVE(t) and CSL(t) and not COR(t) and COA(t) and not MAML+P53_P(t) )* |
| *HIF1A (t+1) = (NUC_STAT3(t) )* |
| *IAP (t+1) = (NUC_NICD1(t) and COA(t) and CSL(t) and not COR(t) )* |
| *MYC (t+1) = (NUC_NICD1(t) and YY1(t) )* |
| *NECD1 (t+1) = (JAG1(t) and NOTCH1(t) and TACE(t) ) or (JAG2(t) and NOTCH1(t) and TACE(t) ) or (DLL1(t) and NOTCH1(t) and TACE(t) ) or (DLL3(t) and NOTCH1(t) and TACE(t) ) or (DLL4(t) and NOTCH1(t) and TACE(t) ) or (MAGP1(t) and NOTCH1(t) ) or (MAGP2(t) and NOTCH1(t) ) or (NOV(t) and NOTCH1(t) ) or (CNTN1(t) and NOTCH1(t) )* |
| *NECD2 (t+1) = (JAG1(t) and NOTCH2(t) and TACE(t) ) or (JAG2(t) and NOTCH2(t) and TACE(t) ) or (DLL1(t) and NOTCH2(t) and TACE(t) ) or (DLL3(t) and NOTCH2(t) and TACE(t) ) or (DLL4(t) and NOTCH2(t) and TACE(t) ) or (CNTN1(t) and NOTCH2(t) )* |
| *NECD3 (t+1) = (JAG1(t) and NOTCH3(t) and TACE(t) ) or (JAG2(t) and NOTCH3(t) and TACE(t) ) or (DLL1(t) and NOTCH3(t) and TACE(t) ) or (DLL3(t) and NOTCH3(t) and TACE(t) ) or (DLL4(t) and NOTCH3(t) and TACE(t) )* |
| *NECD4 (t+1) = (JAG1(t) and NOTCH4(t) and TACE(t) ) or (JAG2(t) and NOTCH4(t) and TACE(t) ) or (DLL1(t) and NOTCH4(t) and TACE(t) ) or (DLL3(t) and NOTCH4(t) and TACE(t) ) or (DLL4(t) and NOTCH4(t) and TACE(t) )* |

| |
|---|
| *NEXT1 (t+1) = (JAG1(t) and NOTCH1(t) and TACE(t) ) or (JAG2(t) and NOTCH1(t) and TACE(t) ) or (DLL1(t) and NOTCH1(t) and TACE(t) ) or (DLL3(t) and NOTCH1(t) and TACE(t) ) or (DLL4(t) and NOTCH1(t) and TACE(t) ) or (MAGP1(t) and NOTCH1(t) ) or (MAGP2(t) and NOTCH1(t) ) or (NOV(t) and NOTCH1(t) ) or (CNTN1(t) and NOTCH1(t) )* |
| *NEXT2 (t+1) = (JAG1(t) and NOTCH2(t) and TACE(t) ) or (JAG2(t) and NOTCH2(t) and TACE(t) ) or (DLL1(t) and NOTCH2(t) and TACE(t) ) or (DLL3(t) and NOTCH2(t) and TACE(t) ) or (DLL4(t) and NOTCH2(t) and TACE(t) ) or (CNTN1(t) and NOTCH2(t) )* |
| *NEXT3 (t+1) = (JAG1(t) and NOTCH3(t) and TACE(t) ) or (JAG2(t) and NOTCH3(t) and TACE(t) ) or (DLL1(t) and NOTCH3(t) and TACE(t) ) or (DLL3(t) and NOTCH3(t) and TACE(t) ) or (DLL4(t) and NOTCH3(t) and TACE(t) )* |
| *NEXT4 (t+1) = (JAG1(t) and NOTCH4(t) and TACE(t) ) or (JAG2(t) and NOTCH4(t) and TACE(t) ) or (DLL1(t) and NOTCH4(t) and TACE(t) ) or (DLL3(t) and NOTCH4(t) and TACE(t) ) or (DLL4(t) and NOTCH4(t) and TACE(t) )* |
| *NICD_ACTIVE (t+1) = (NUC_NICD1(t) ) or (NUC_NICD2(t) ) or (NUC_NICD3(t) ) or (NUC_NICD4(t) )* |
| *NICD1 (t+1) = (NEXT1(t) and GAMMA_SECRETASE(t) )* |
| *NICD2 (t+1) = (NEXT2(t) and GAMMA_SECRETASE(t) )* |
| *NICD3 (t+1) = (NEXT3(t) and GAMMA_SECRETASE(t) )* |
| *NICD4 (t+1) = (NEXT4(t) and GAMMA_SECRETASE(t) )* |
| *NOTCH1 (t+1) = (FURIN(t) and not NUMB(t) and not ITCH(t) and ALPHA_ADAPTIN(t) and NOTCH1_PRE(t) ) or (not NEDD4(t) and NOTCH1_PRE(t) ) or (POGLUT_1(t) and O_GLUCOSE(t) and NOTCH1_PRE(t) ) or (XYL(t) and O_GLUCOSE(t) and not XYLE(t) and NOTCH1_PRE(t) ) or (O_FUCOSE(t) and NGA(t) and FRINGE(t) and POFUT_1(t) and NOTCH1_PRE(t) ) or (GALACTOSE(t) and GASE(t) and O_FUCOSE(t) and NOTCH1_PRE(t) )* |
| *NOTCH1_PRE (t+1) = (NUC_NICD1(t) and COA(t) and CSL(t) and not COR(t) )* |
| *NOTCH2 (t+1) = (FURIN(t) and not NUMB(t) and not ITCH(t) and ALPHA_ADAPTIN(t) and NOTCH2_PRE(t) ) or (POGLUT_1(t) and O_GLUCOSE(t) and NOTCH2_PRE(t) ) or (XYL(t) and O_GLUCOSE(t) and not XYLE(t) and NOTCH2_PRE(t) ) or (O_FUCOSE(t) and NGA(t) and FRINGE(t) and POFUT_1(t) and NOTCH2_PRE(t) ) or (GALACTOSE(t) and GASE(t) and O_FUCOSE(t) and NOTCH2_PRE(t) )* |
| *NOTCH2_PRE (t+1) = (NUC_NICD2(t) and COA(t) and CSL(t) and not COR(t) )* |
| *NOTCH3 (t+1) = (POGLUT_1(t) and O_GLUCOSE(t) and NOTCH3_PRE(t) ) or (XYL(t) and O_GLUCOSE(t) and not XYLE(t) and NOTCH3_PRE(t) ) or (O_FUCOSE(t) and NGA(t) and FRINGE(t) and POFUT_1(t) and NOTCH3_PRE(t) ) or (GALACTOSE(t) and GASE(t) and O_FUCOSE(t) and NOTCH3_PRE(t) )* |
| *NOTCH3_PRE (t+1) = (NUC_NICD3(t) and COA(t) and CSL(t) and not COR(t) )* |
| *NOTCH4 (t+1) = (POGLUT_1(t) and O_GLUCOSE(t) and NOTCH4_PRE(t) ) or (XYL(t) and O_GLUCOSE(t) and not XYLE(t) and NOTCH4_PRE(t) ) or (O_FUCOSE(t) and NGA(t) and FRINGE(t) and POFUT_1(t) and NOTCH4_PRE(t) ) or (GALACTOSE(t) and GASE(t) and O_FUCOSE(t) and NOTCH4_PRE(t) )* |
| *NOTCH4_PRE (t+1) = (NUC_NICD4(t) and COA(t) and CSL(t) and not COR(t) )* |
| *NOX (t+1) = (P53_P(t) )* |
| *NRARP (t+1) = (NICD_ACTIVE(t) and CSL(t) and not COR(t) and COA(t) )* |
| *NUC_NICD1 (t+1) = (GSK_3BETA(t) and not DVL(t) and not JIP1 (t) and NICD1(t) ) or (RAS(t) and NICD1(t) ) or (WDR12(t) and NICD1(t) ) or (MAML(t) and not CDK8(t) and not CYCC(t) and not FBW7(t) and NICD1(t) ) or (not NRARP(t) and NICD1(t) ) or (HIF1A(t) and NICD1(t) )* |
| *NUC_NICD2 (t+1) = (GSK_3BETA(t) and not DVL(t) and not JIP1 (t) and NICD2(t) ) or (MAML(t) and not CDK8(t) and not CYCC(t) and not FBW7(t) and NICD2(t) ) or (not NRARP(t) and NICD2(t) ) or (HIF1A(t) and NICD2(t) )* |

| |
|---|
| *NUC_NICD3 (t+1) = (GSK_3BETA(t) and not DVL(t) and not JIP1 (t) and NICD3(t) ) or (MAML(t) and not CDK8(t) and not CYCC(t) and not FBW7(t) and NICD3(t) ) or (not NRARP(t) and NICD3(t) ) or (HIF1A(t) and NICD3(t) )* |
| *NUC_NICD4 (t+1) = (GSK_3BETA(t) and not DVL(t) and not JIP1 (t) and NICD4(t) ) or (not FBW7(t) and NICD4(t) ) or (MAML(t) and not CDK8(t) and not CYCC(t) and not FBW7(t) and NICD4(t) ) or (not NRARP(t) and NICD4(t) ) or (HIF1A(t) and NICD4(t) )* |
| *NUC_STAT3 (t+1) = (STAT3_P(t) )* |
| *P53_P (t+1) = (P53(t) and not AKT(t) and not NICD1(t) )* |
| *PI3K (t+1) = (not PTEN(t) )* |
| *PTEN (t+1) = (not HES1(t) )* |
| *PUMA (t+1) = (P53_P(t) )* |
| *STAT3_P (t+1) = (HES1(t) and JAK2(t) and STAT3(t) ) or (HES5(t) and JAK2(t) and STAT3(t) )* |
| *BAD (t+1) = (not AKT(t) )* |
| *TENASCIN_C (t+1) = (NUC_NICD1(t) and COA(t) and CSL(t) and not COR(t) ) or (NUC_NICD2(t) and COA(t) and CSL(t) and not COR(t) )* |
| *GFAP (t+1) = (NUC_STAT3(t) )* |
| *NGN1 (t+1) = (not DTX1(t) and EP300(t) and MASH1(t) )* |
| *BETA_TUBULIN_III (t+1) = (NGN1(t) )* |
| *NESTIN (t+1) = (not HES1(t) and not HES5(t) )* |
| *MASH1 (t+1) = (not HES1(t) and not HES5(t) )* |
| *NEUROD (t+1) = (not HES1(t) and not HES5(t) )* |
| **APOPTOSIS (t+1) = (not FLIP(t) and not IAP(t) and not BCL2(t) and BAD(t) and PUMA(t) and NOX(t) and BAX(t) )* |
| **NPC DIFFERENTIATION (t+1) = (BETA_TUBULIN_III(t) and NEUROD(t) and NESTIN(t) and not APOPTOSIS(t) and not ASPC DIFFERENTIATION(t) and not GBM DEVELOPMENT(t) )* |
| **ASPC DIFFERENTIATION (t+1) = (GFAP(t) and not APOPTOSIS(t) and not NPC DIFFERENTIATION(t) and not GBM DEVELOPMENT(t) )* |
| **GBM DEVELOPMENT (t+1) = (CCND3(t) and CCND1(t) and CDK2(t) and MYC(t) and TENASCIN_C(t) and GFAP(t) and not APOPTOSIS(t) and not NPC DIFFERENTIATION(t) )* |
| **NSC RENEWAL (t+1) = (CCND3(t) and CCND1(t) and CDK2(t) and not APOPTOSIS(t) and not NPC DIFFERENTIATION(t) and not GBM DEVELOPMENT(t) and not ASPC_PROLIFERATION(t) )* |

*** Phenotypes of the model. R.H.S = Logical states of the nodes at $t^{th}$ time point. L.H.S = Logical state of the node at $(t+1)^{th}$ time point.**

All the nodes considered in the logical model are further classified into four sub-classes: ***(i) Inputs, (ii) Intermediates, (iii) Targets,*** and ***(iv) Phenotypes*****.** A detailed description of all the nodes included in the model is given below (Supplementary Table 2).

## Supplementary Table 2: Detail descriptions of the nodes used in the logical model

| **Input Molecules** | | | | | | |
|---|---|---|---|---|---|---|
| **Sr. No.** | **Names used in the Model** | **Type of the Molecules** | **Full Name/Gene Name of the Molecules** | **Short Name** | **Uniprot ID** | **Ensemble ID** |
| 1 | ALPHA_ADAP TIN | Protein | AP-2 complex subunit beta | AP2B1 | P63010 | ENSG00000006125 |
| 2 | APH1 | Protein | Gamma-secretase subunit APH-1A | APH1A | Q96BI3 | ENSG00000117362 |
| 3 | CDK8 | Protein | Cyclin dependent kinase 8 | CDK8 | P49336 | ENSG00000132964 |
| 4 | CIR | Protein | Corepressor interacting with RBPJ 1 | CIR1 | Q86X95 | ENSG00000138433 |
| 5 | CNTN1 | Protein | Contactin-1 | CNTN1 | Q12860 | ENSG00000018236 |
| 6 | CSL | Protein | Recombining binding protein suppressor of hairless | RBPJ | Q06330 | ENSG00000168214 |
| 7 | CYC | Protein | Cyclin-C | CCNC | P24863 | ENSG00000112237 |
| 8 | DLL1 | Protein | Delta-like protein 1 | DLL1 | O00548 | ENSG00000198719 |
| 9 | DLL3 | Protein | Delta-like protein 3 | DLL3 | Q9NYJ7 | ENSG00000090932 |
| 10 | DLL4 | Protein | Delta-like protein 4 | DLL4 | Q9NR61 | ENSG00000128917 |
| 11 | DTX1 | Protein | Deltex E3 ubiquitin ligase 1 | DTX1 | Q86Y01 | ENSG00000135144 |
| 12 | DVL1 | Protein | Segment polarity protein dishevelled homolog DVL-1 | DVL1 | O14640 | ENSG00000107404 |
| 13 | EP300 | Protein | E1A binding protein p300 | EP300 | Q09472 | ENSG00000100393 |
| 14 | FBW7 | Protein | F-box/WD repeat-containing protein 7 | FBXW7 | Q969H0 | ENSG00000109670 |
| 15 | FRINGE | Protein | Beta-1,3-N-acetylglucosaminyltrans ferase lunatic fringe | LFNG | Q8NES3 | ENSG00000106003 |
| 16 | FURIN | Protein | Furin | FURIN | P09958 | ENSG00000140564 |
| 17 | GALACTOSE | Metabolite | Galactose | NA | NA | NA |
| 18 | GASE | Protein | β1,4-galactosyl-transferase | B4GALT1 | P15291 | ENSG00000086062 |
| 19 | GSK_3BETA | Protein | Glycogen synthase kinase-3 beta | GSK3B | P49841 | ENSG00000082701 |
| 20 | HAT | Protein | Histone acetyltransferase 1 | HAT1 | Q09472 | ENSG00000128708 |

| | | | | | | |
|---|---|---|---|---|---|---|
| 21 | HDAC | Protein | Histone deacetylase 1 | HDAC1 | Q13547 | ENSG00000116478 |
| 22 | ITCH | Protein | E3 ubiquitin-protein ligase Itchy homolog | ITCH | Q96J02 | ENSG00000078747 |
| 23 | JAG1 | Protein | jagged-1 | JAG1 | P78504 | ENSG00000101384 |
| 24 | JAG2 | Protein | jagged-2 | JAG2 | Q9Y219 | ENSG00000184916 |
| 25 | JAK2 | Protein | Tyrosine-protein kinase JAK2 | JAK2 | O60674 | ENSG00000096968 |
| 26 | JIP1 | Protein | C-Jun-amino-terminal kinase-interacting protein 1 | MAPK8IP1 | Q9UQF2 | ENSG00000121653 |
| 27 | MAGP1 | Protein | Microfibril-associated glycoprotein 1 | MFAP2 | P55001 | ENSG00000117122 |
| 28 | MAGP2 | Protein | Microfibril-associated glycoprotein 2 | MFAP5 | Q13361 | ENSG00000197614 |
| 29 | MAML | Protein | Mastermind-like protein 1 | MAML1 | Q92585 | ENSG00000161021 |
| 30 | NEDD4 | Protein | E3 ubiquitin-protein ligase NEDD4 | NEDD4 | P46934 | ENSG00000069869 |
| 31 | NGA | Metabolite | N-acetylglucosamine | NA | NA | NA |
| 32 | NICASTRIN | Protein | Nicastrin | NCSTN | Q92542 | ENSG00000162736 |
| 33 | NOV | Protein | Nephroblastoma overexpressed | NOV | P48745 | ENSG00000136999 |
| 34 | NUMB | Protein | Protein numb homolog | NUMB | P49757 | ENSG00000133961 |
| 35 | O_FUCOSE | Metabolite | O-Fucose | NA | NA | NA |
| 36 | O_GLUCOSE | Metabolite | O-GLUCOSE | NA | NA | NA |
| 37 | P53 | Protein | Cellular tumor antigen p53 | TP53 | P04637 | ENSG00000141510 |
| 38 | PEN2 | Protein | Presenilin enhancer gamma-secretase subunit | PSENEN | Q9NZ42 | ENSG00000205155 |
| 39 | POFUT_1 | Protein | GDP-fucose protein O-fucosyltransferase 1 | POFUT1 | Q9H488 | ENSG00000101346 |
| 40 | POGLUT_1 | Protein | Protein O-glucosyltransferase 1 | POGLUT1 | Q8NBL1 | ENSG00000163389 |
| 41 | PRESENILIN1 | Protein | Presenilin 1 | PSEN1 | P49768 | ENSG00000080815 |
| 42 | RAS | Protein | GTPase HRas | HRAS | P01112 | ENSG00000174775 |
| 43 | SAP30 | Protein | Histone deacetylase complex subunit SAP30 | SAP30 | O75446 | ENSG00000164105 |
| 44 | SIN3A | Protein | Paired amphipathic helix protein Sin3a | SIN3A | Q96ST3 | ENSG00000169375 |

| | | | | | | |
|---|---|---|---|---|---|---|
| 45 | SKIP | Protein | SKI Interacting Protein | SNW1 | Q13573 | ENSG00000100603 |
| 46 | SMAD3 | Protein | Mothers against decapentaplegic homolog 3 | SMAD3 | P84022 | ENSG00000166949 |
| 47 | SMRT | Protein | Nuclear receptor corepressor 2 | NCOR2 | Q9Y618 | ENSG00000196498 |
| 48 | STAT3 | Protein | Signal transducer and activator of transcription 3 | STAT3 | P40763 | ENSG00000168610 |
| 49 | TACE | Protein | Disintegrin and metalloproteinase domain-containing protein 17 | ADAM17 | P78536 | ENSG00000151694 |
| 50 | WDR12 | Protein | Ribosome biogenesis protein WDR12 | WDR12 | Q9GZL7 | ENSG00000138442 |
| 51 | XYL | Metabolite | Xylose | NA | NA | NA |
| 52 | XYLE | Protein | α1,3-xylosyltransferase | XXYLT1 | Q8NBI6 | ENSG00000173950 |
| 53 | YY1 | Protein | YY1 transcription factor | YY1 | P25490 | ENSG00000100811 |
| **Intermediate Molecules** | | | | | | |
| 1 | AKT | Protein | AKT serine/threonine kinase 1 | AKT1 | P31749 | ENSG00000142208 |
| 2 | CD44 | Protein | CD44 antigen | CD44 | P16070 | ENSG00000026508 |
| 3 | CDK2 | Protein | Cyclin-dependent kinase 2 | CDK2 | P24941 | ENSG00000123374 |
| 4 | NOTCH1 | Protein | NOTCH1 | NOTCH1 | P46531 | ENSG00000148400 |
| 5 | NOTCH2 | Protein | NOTCH2 | NOTCH2 | Q04721 | ENSG00000134250 |
| 6 | NOTCH3 | Protein | NOTCH3 | NOTCH3 | Q9UM47 | ENSG00000074181 |
| 7 | NOTCH4 | Protein | NOTCH4 | NOTCH4 | Q99466 | ENSG00000204301 |
| 8 | PI3K | Protein | Phosphatidylinositol 3-kinase regulatory subunit alpha | PIK3R1 | P27986 | ENSG00000145675 |
| 9 | PTEN | Protein | Phosphatidylinositol 3,4,5-trisphosphate 3-phosphatase | PTEN | P60484 | ENSG00000171862 |
| 10 | MAML+P53_P | Protein Complex | MAML and P53 proteins complex | NA | NA | NA |
| 11 | COA | Protein Complex | Transcription Co-activator Complex | NA | NA | NA |
| 12 | COR | Protein Complex | Transcription Co-repressor Complex | NA | NA | NA |
| 13 | GAMMA_SECRETASE | Protein Complex | Gamm Secreatase enzyme complex | NA | NA | NA |

| | | | | | | |
|---|---|---|---|---|---|---|
| 14 | HES1_MRNA | mRNA Molecule | mRNA of HES1 Protein | NA | NA | NA |
| 15 | HES5_MRNA | mRNA Molecule | mRNA of HES5 Protein | NA | NA | NA |
| 16 | NECD1 | Truncated Protein | Notch extra-cellular domain 1 | NA | NA | NA |
| 17 | NECD2 | Truncated Protein | Notch extra-cellular domain 2 | NA | NA | NA |
| 18 | NECD3 | Truncated Protein | Notch extra-cellular domain 3 | NA | NA | NA |
| 19 | NECD4 | Truncated Protein | Notch extra-cellular domain 4 | NA | NA | NA |
| 20 | NEXT1 | Truncated Protein | Notch extra-cellular truncated domain 1 | NA | NA | NA |
| 21 | NEXT2 | Truncated Protein | Notch extra-cellular truncated domain 2 | NA | NA | NA |
| 22 | NEXT3 | Truncated Protein | Notch extra-cellular truncated domain 3 | NA | NA | NA |
| 23 | NEXT4 | Truncated Protein | Notch extra-cellular truncated domain 4 | NA | NA | NA |
| 24 | NICD1 | Truncated Protein | Notch intra-cellular domain 1 | NA | NA | NA |
| 25 | NICD2 | Truncated Protein | Notch intra-cellular domain 2 | NA | NA | NA |
| 26 | NICD3 | Truncated Protein | Notch intra-cellular domain 3 | NA | NA | NA |
| 27 | NICD4 | Truncated Protein | Notch intra-cellular domain 4 | NA | NA | NA |
| 28 | NICD_ACTIVE* | Hypothetical Node* | Transcriptionally active Notch Intra-cellular domain | NA | NA | NA |
| 29 | NOTCH1_PRE | Precursor Protein | Precussor of Notch1 receptor protein | NA | NA | NA |
| 30 | NOTCH2_PRE | Precursor Protein | Precussor of Notch2 receptor protein | NA | NA | NA |
| 31 | NOTCH3_PRE | Precursor Protein | Precussor of Notch3 receptor protein | NA | NA | NA |
| 32 | NOTCH4_PRE | Precursor Protein | Precussor of Notch4 receptor protein | NA | NA | NA |
| 33 | NUC_NICD1 | Nuclear Counterpart | Nuclear counterpart of NICD1 | NA | NA | NA |
| 34 | NUC_NICD2 | Nuclear Counterpart | Nuclear counterpart of NICD2 | NA | NA | NA |

| | | | | | | |
|---|---|---|---|---|---|---|
| 35 | NUC_NICD3 | Nuclear Counterpart | Nuclear counterpart of NICD3 | NA | NA | NA |
| 36 | NUC_NICD4 | Nuclear Counterpart | Nuclear counterpart of NICD4 | NA | NA | NA |
| 37 | NUC_STAT3 | Nuclear Counterpart | Nuclear counter of STAT3 protein | NA | NA | NA |
| 38 | P53_P | Phosphorylated Protein | Phosphorylated form of P53 protein | NA | NA | NA |
| 39 | STAT3_P | Phosphorylated Protein | Phosphorylated form of STAT3 protein | NA | NA | NA |
| **Target Proteins** | | | | | | |
| 1 | BAX | Protein | BCL2 associated X | BAX | Q07812 | ENSG00000087088 |
| 2 | BAD | Protein | Bcl2-associated agonist of cell death | BAD | Q92934 | ENSG00000002330 |
| 3 | BCL2 | Protein | Apoptosis regulator Bcl-2 | BCL2 | P10415 | ENSG00000171791 |
| 4 | CCND1 | Protein | G1/S-specific cyclin-D1 | CCND1 | P24385 | ENSG00000110092 |
| 5 | CCND3 | Protein | G1/S-specific cyclin-D3 | CCND3 | P30281 | ENSG00000112576 |
| 6 | FLIP | Protein | CASP8 and FADD-like apoptosis regulator | CFLAR | O15519 | ENSG00000003402 |
| 7 | HES1 | Protein | HES1 | HES1 | Q14469 | ENSG00000114315 |
| 8 | HES5 | Protein | HES5 | HES5 | Q5TA89 | ENSG00000197921 |
| 9 | HES7 | Protein | HES7 | HES7 | Q9BYE0 | ENSG00000179111 |
| 10 | HEY1 | Protein | HEY1 | HEY1 | Q9Y5J3 | ENSG00000164683 |
| 11 | HEY2 | Protein | HEY2 | HEY2 | Q9UBP5 | ENSG00000135547 |
| 12 | HEYL | Protein | Hairy/enhancer-of-split related with YRPW motif-like protein | HEYL | Q9NQ87 | ENSG00000163909 |
| 13 | HIF1A | Protein | Hypoxia-inducible factor 1-alpha | HIF1A | Q16665 | ENSG00000100644 |
| 14 | IAP | Protein | E3 ubiquitin-protein ligase XIAP | XIAP | P98170 | ENSG00000101966 |
| 15 | MYC | Protein | Myc proto-oncogene protein | MYC | P01106 | ENSG00000136997 |
| 16 | NOX | Protein | NADPH oxidase 1 | NOX1 | Q9Y5S8 | ENSG00000007952 |
| 17 | NRARP | Protein | Notch-regulated ankyrin repeat-containing protein | NRARP | Q7Z6K4 | ENSG00000198435 |
| 18 | PUMA | Protein | Bcl-2-binding component 3 | BBC3 | Q9BXH1 | ENSG00000105327 |

| | | | | | | |
|---|---|---|---|---|---|---|
| 19 | GFAP | Protein | Glial fibrillary acidic protein | GFAP | P14136 | ENSG00000131095 |
| 20 | NGN1 | Protein | Neurogenin-1 | NEUROG1 | Q92886 | ENSG00000181965 |
| 21 | BETA_TUBULIN_III | Protein | Tubulin beta-3 chain | TUBB3 | Q13509 | ENSG00000258947 |
| 22 | NESTIN | Protein | Nestin | NES | P48681 | ENSG00000132688 |
| 23 | MASH1 | Protein | Achaete-scute homolog 1 | ASCL1 | P50553 | ENSG00000139352 |
| 24 | NEUROD | Protein | Neurogenic differentiation factor 1 | NEUROD1 | Q13562 | ENSG00000162992 |
| 25 | TENASCIN_C | Protein | Tenascin | TNC | P24821 | ENSG00000041982 |
| **Phenotypes** | | | | | | |
| 1 | Apoptosis | NA | Apotosis or Natural Cell death | NA | NA | NA |
| 2 | NPC Differentiation | NA | Differentiations of Neural progenitor cells | NA | NA | NA |
| 3 | NPC Differentiation | NA | Differentiations of Astrocyte progenitor cells | NA | NA | NA |
| 4 | GBM Development | NA | Differentiated and developed Glioblastoma cells | NA | NA | NA |
| 5 | NSC Renewal | NA | Neural stem cell renewal | NA | NA | NA |

*** Hypothetical node is introduced for the simplification of the model simulation**

## *1.2 Marker proteins of aNSC, GSC and GBM models*

The marker proteins whose expressions are analyzed to observe the dynamics of different cellular states or phenotypes in the aNSC, GSC and General glioma models are enlisted, with appropriate references, in Supplementary Table 3. The temporal dynamics of all the attractor states observed in these three models are also shown in "Data Analyses" section (sub-section 3.2). Oscillatory behaviors of the state transitions of marker proteins are observed in the corresponding cyclic attractor states (e.g. NSC Differentiation), whereas steady state saturation (high or low) is observed for the marker proteins in fixed-point attractor states (e.g. Quiescent state).

**Supplementary Table 3. Information of the marker proteins mapped with different phenotypes**

| Cellular Phenotypes | Marker Proteins | References |
|---|---|---|
| Apoptosis | Pro-apoptotic: PUMA, NOX, BAD, BAX | [8,9] |
| | Anti-apoptotic: FLIP, IAP, BCL2 | |
| Neural Stem Cells Renewal | CYCLIN-D3, CYCLIN-D1, CDK, HES1, HES5 | [10-12] |
| Neural Progenitor Cells | NESTIN, NEUROD, β-TUBULIN [13]-III | [14] |
| Astrocyte Progenitor Cells | GFAP | [15] |
| Glioblastoma Stem Cells | CYCLIN-D3, CYCLIN-D1, CDK, HES1, HES5, FLIP, IAP, BCL2 | [16] |
| Glioblastoma tumor Cells | CYCLIN-D3, CYCLIN-D1, CDK, C-MYC, TENASCIN-C, GFAP | [17,18] |

Expression patterns of each of these proteins in different attractor states (or cellular states) observed in aNSC, GSC and General glioma models are described in the later sections (Supplementary Figs 2,3, and 4). The expressions of these marker proteins are denoted in the model simulation by discrete binary states 0 or 1. The occurrences of a particular phenotypic/cellular state in the model are mapped with a Boolean function of the corresponding marker proteins of that phenotype and therefore its expression values (or activity patterns) will be varied in the discrete binary domain of *{0, 1}*.

$$f : M \to P$$
$$f := Boolean\,Function$$
$$P := Phenotypes \in \{p_1, p_2, p_3, ....\} \in \{0,1\}$$
$$M := Marker\ Proteins \in \{m_1, m_2, m_3 ....\} \in \{0,1\}$$

### *1.3 Determining the phenotypes and cellular states*

There are total five phenotypic functions considered in the developed dynamic model, which are dependent on the expression of specific marker proteins of normal and tumorigenic brain cells. These five phenotypes are *i) Apoptosis, ii) NSC Renewal, iii) NPC Differentiation, iv) ASPC Differentiation,* and *v) GBM Development*. Depending on the distribution of input signals different marker proteins are expressed with different distribution, which in turn will regulate the expressions of different phenotype in the binary domain $\{1,0\}$. Theoretically, it can be considered that in total $2^5$ or 32 single and combinations of phenotypes are possible to be occurred with equal probability in the attractor distribution space. The probability to reach any of the phenotype would be random and unbiased if the logical model is purely random. Also, in case of a random logical network model, it can also be assumed that the flow of signal transduction within the signaling network would be purely unbiased and can lead the reaction cascades towards the development of

any phenotype under a set of specific intra and extra-cellular stimuli. However, in reality, the topology of the constructed logical model is not completely random and unbiased in nature as the logical rules defined for the Notch signaling network is mainly taken from experimental evidences. The normal functioning of Notch signaling network is specifically oriented towards the neural stem cell renewal and its differentiation into neural progenitor cells [19]. Similar to the experimental findings, it is also observed that at the time of Notch pathway simulation, only a few phenotypic states occur in the attractor space and those states are denoted as "Cellular states". A cellular state can be an individual phenotype (such as Apoptosis) or a combination of multiple phenotypes (e.g. NSC Renewal/NPC Differentiation). The following attributes are also considered during the model simulation to define two typical cellular states: Quiescent cells and GSC Renewal.

$$Cellular\ States := Quiescent(t) = 1 \ \ iff\ \forall\ Phenotypes : P_i(t) \in \{0\}; where, i = 1, 2, ..., 5$$
$$GSC\ Renewal(t) = NSC\ Renewal(t)\ and\ not\ Apoptosis(t)$$

*Quiescent* state is a distinct cellular state, which is found at ON state only when all the phenotypes are at OFF state. In this state all other cellular activities such as proliferation, differentiation and apoptosis are found at dormant stage or at lower rate [20]. Similarly, *GSC renewal* will be at ON state if the phenotype *Apoptosis* is at OFF state and *NSC Renewal* is at ON state.

It is also observed that based on the steady state distributions and the temporal expressions patterns (1 or 0) of the intermediate and target molecules of Notch pathway, these cellular states can be found in either *"Fixed-point"* or *"Periodic"* attractors space. In the fixed-point state, the binary expressions of the cellular state and the pathway molecules will show homeostatic behavior, whereas in the periodic state it will be oscillatory in nature.

### *1.4 Calculation of normalized frequencies of cellular states, Shannon entropy and Activity Ratio (AR) scores*

Earlier it is mentioned that exploring the entire input space of the 53 input nodes (i.e. $2^{53}$ = ~ 9.0071993 X $10^{15}$) of this logical model to find out all possible attractor states is a computationally challenging task. Hence, to reduce the computational cost and time, a heuristic search algorithm (simulation based) is used here to find the maximum number of attractors of this logical model. The basic criterion of this algorithm is to run the simulation by taking randomly non-redundant, finite set of initial states (say $N = 10^6$) from the overall population (9.0071993 X $10^{15}$) of input states. The random initial states are further divided into 100 separate batches (i.e. each batch will contain 10,000 random input sequences) and the frequencies of all the attractor states (or cellular states) are calculated for each batch of simulation. The frequencies of each cellular state are further normalized by dividing its total frequency observed in a simulation batch with respect to the total number of random initial conditions (i.e. 10,000) used for simulation. It should be noted that degeneracy of the observed attractor states with respect to a particular cellular state is possible, which means multiple sets of similar attractor states can be mapped with a particular cellular state depending on the expression state(s) of the marker protein(s) in those attractor distribution $\Pi(A)$.

Let us consider that such attractor distributions are calculated from the initial input states distribution $\Pi(I)$ in any of the simulation batch. Hence, it can be said that the distribution of the cellular states $\Pi(C)$ is also dependent on the distribution of the attractor states $\Pi(A)$ and can be mapped with Boolean function $f:\Pi(A)\rightarrow\Pi(C)$.

Let us also consider that in an arbitrary batch of simulation ($B_i$), there exists a set of cellular states $C_i=\left\{C_i^1,C_i^2,C_i^3,.....,C_i^m\right\}$. Here, '*i*' is the simulation batch number and '*m*' is the total number of observed cellular states or the size of maximum information content possible to be observed in the attractor distribution $\Pi(A)$ generated in the simulation draw $B_i$.

If the probability mass functions (i.e. information) of all the elements of the sets of cellular states $(C_i)$ drawn from the simulation batches (B = 100) are taken into the consideration, then according to the "principle of maximum entropy" the probability mass function with highest information entropy will be chosen as the "proper one" for further data analyses [21]. The information entropy of a given batch of simulation can be calculated by calculating the Shannon entropy score of the observed cellular states.

***Definition 1: The Shannon entropy H(C$_i$) of the i$^{th}$ instance of a simulation containing the distribution of cellular states*** $C_i=\left\{C_i^1,C_i^2,C_i^3,.....,C_i^m\right\}$ ***is the negative logarithm of the probability mass function of the observed cellular states in that instance of simulation.***

$$Hence,\ Shannon\,Entropy = H(C_i) = E[-\ln(\mathrm{P}(C_i))]$$

$$= -\sum_{m=1}^{m}\mathrm{P}(C_i^m).\log_2\mathrm{P}(C_i^m) \qquad (3)$$

***Theorem 1: If*** $\Pi(A_1),\Pi(A_2),\Pi(A_3),....,\Pi(A_n)$ ***are the attractor distributions generated from initial input states*** $\Pi(I_1),\Pi(I_2),\Pi(I_3),.....,\Pi(I_n)$ ***and their corresponding normalized frequency distributions of cellular states are*** $\Pi(C_1),\Pi(C_2),\Pi(C_3),.....,\Pi(C_n)$ ***, then the simulation instance containing highest Shannon entropy score H(C$_i$) will posses maximum numbers of different types of cellular states*** $C_i=\left\{C_i^1,C_i^2,C_i^3,.....,C_i^m\right\}$.

**Proof:** Let us assume that *i=1, 2, 3,...n* are the different instances of Boolean simulations performed under a set of equal number of initial expressions vectors (say *N*), in which the expressions of the elements (i.e. input molecules) are randomly distributed. Let us consider the distribution of the random initial expressions sequences is $\Pi(I_i)$. Let us also consider that each of the simulation instances is producing a distribution of attractor states $\Pi(A_i)$, which can be mapped with a distribution of cellular states $f:\Pi(A_i)\rightarrow\Pi(C_i)$. Here, $C_i=\left\{C_i^1,C_i^2,C_i^3,.....,C_i^m\right\}$ is the normalized frequency of the individual cellular states (total numbers *m*) observed in the attractor distribution $\Pi(A_i)$ of $i^{th}$ simulation instance. It could be possible that the total number of individual cellular states *(m)* observed in a particular simulation instance may not be equal for all the simulation instances.

Let us assume that the probabilities of each of the cellular states in a given simulation instances are $\mathrm{P}(C_i^1), \mathrm{P}(C_i^2), \mathrm{P}(C_i^3), ...., \mathrm{P}(C_i^m)$.

If there are $p^1, p^2, p^3, ...., p^z \in \{0,1\}$ numbers of fixed phenotypes considered in the model, which are mapped with the cellular states $C_i = \{C_i^1, C_i^2, C_i^3, ....., C_i^m\}$, then theoretically there exist maximum $M\ (=2^z)$ numbers of cellular states and the maximum Shannon entropy score by ***definition 1*** will be $Max\left(H(C)\right) = -\sum_{m=1}^{M} \mathrm{P}(C^m).\log_2 \mathrm{P}(C^m)$.

Hence, the maximum Shannon entropy of any arbitrary simulation instance *'i'* is,

$$
\begin{aligned}
&\mathrm{Max}\,(H(C_i)) = -\sum_{m=1}^{M} \mathrm{P}(C_i^m).\log_2 \mathrm{P}(C_i^m).\\
&\textit{Here}, \; -\mathrm{P}(C_i^m).\log_2 \mathrm{P}(C_i^m) \geq 0\\
&\textit{Since}, \quad \log_2 \mathrm{P}(C_i^m) \leq 0 \quad \forall \quad \mathrm{P}(C_i^m) > 0\\
&\textit{For any arbitrary number of cellular states 'm'},\\
&\quad -\sum_{m=1}^{m} \mathrm{P}(C_i^m).\log_2 \mathrm{P}(C_i^m) \geq 0\\
&\textit{Therefore, when } m \rightarrow \max(m),\\
&-\sum_{m=1}^{\max(m)} \mathrm{P}(C_i^m).\log_2\{\mathrm{P}(C_i^m)\} \leq \mathrm{Max}\,(H(C_i)).
\end{aligned}
$$

Hence, it is proven that at the level of maximum Shannon entropy score, there exists maximum number of distinct cellular states (i.e. *max(m)*).

***Activity Ratio Score:*** This novel scoring technique is a metric to determine the contribution of an arbitrary pathway molecule ($X_i$) in the cell-signaling network to drive the cellular dynamics towards a particular cellular state. This scoring technique is specifically useful for extracting the important proteins from the set of input proteins, which are helping the signaling cascade to reach the specific phenotype. In the pathway simulation, given a set of total random input sequences (N), if $S_k$ is the total number of random input sequences which direct the model simulation towards a particular cellular state $(C_i^m)$, then the activity ratio of any arbitrary input molecule ($X_i$) will be calculated by using the following equations (***Eq. 4** and **5***).

$$Activity\,Ratio = \left(AR\right)_{X_i \in X|C^j \in C} = Log_2\left(e^{\gamma_{X_i}}\right)_{X_i \in X|C^j \in C} \quad (4)$$

$$(\gamma)_{X_i \in X|C_j \in C} = \frac{1}{|S_k|}\left[\sum_{l=1}^{|S_k|}\left(Y_l : X_i = 1\right) - \sum_{l=1}^{|S_k|}\left(Y_l : X_i = 0\right)\right]_{X_i \in X|C_j \in C} \quad (5)$$

$Let\,us\,assume,\ \ C := Set\,of\,Cellular\,States = \left\{C^1, C^2, C^3, ...., C^m\right\}$

$m = Total\,Number\,Phenotypes\,observed$

$X_i \in X := Set\,of\,\,Input\,Molecules = \{X_1, X_2, X_3, ..., X_{N1}\}$

$N1 = Total\,Input\,proteins$

$Y := Set\,of\,\,Input\,sequences = \{Y_1, Y_2, Y_3, ..., Y_{|S_k|}\}\,reaching\,to\,cellular\,state\,C^j$

$Where, |S_k| = Total\,number\,of\,\,input\,sequences\,reaching\,to\,a\,\,particular\,celllar\,states\,out\,of\,\,N\,random\,sequences$

$i.e. |S_k| \subseteq N$

Here, the proportion $(\gamma)_{X_i \in X|C_j \in C}$ of an input protein $(X_i)$ in a given cellular state $(C_i)$ is defined as the ratio of the difference of the number of times the input protein is up-regulated and down-regulated to the total number random input sequences directed towards a specific cellular state in the simulation.

***Lemma 1: In an arbitrary simulation instance, started from a finite set of random initial conditions, the value of AR score of any input node is bounded in the range of -1.44 to +1.44.***

**Proof:** Let us assume that in an arbitrary simulation instance there are $|S_k|$ numbers of input sequences $(Y = \{Y_1, Y_2, Y_3, ..., Y_{|S_k|}\})$ out of N random sequences generating the cellular state $C_i$.

Hence, it can be written as,

$$\sum_{l=1}^{|S_k|}\left(Y_l : X_i = 1\right) + \sum_{l=1}^{|S_k|}\left(Y_l : X_i = 0\right) = |S_k|$$

*Now,*

$$\text{if}, \sum_{l=1}^{|S_k|}\left(Y_l : X_i = 0\right) \to 0,\ \sum_{l=1}^{|S_k|}\left(Y_l : X_i = 1\right) \le |S_k|$$

$$\Rightarrow \frac{1}{|S_k|}\left(\sum_{l=1}^{|S_k|}\left(Y_l : X_i = 1\right)\right) \le 1$$

$$else, \sum_{l=1}^{|S_k|}\left(Y_l : X_i = 1\right) \to 0,\ \sum_{l=1}^{|S_k|}\left(Y_l : X_i = 0\right) \le |S_k|$$

$$\Rightarrow \frac{1}{|S_k|}\left(\sum_{l=1}^{|S_k|}\left(Y_l : X_i = 0\right)\right) \le 1$$

From Eq. 5, it can be written as,

$$\therefore (\gamma)_{X_i \in X|C_j \in C} = \frac{1}{|S_k|}\left[\sum_{l=1}^{|S_k|}(Y_l : X_i = 1) - 0\right] \le 1$$

*Or,*

$$\therefore (\gamma)_{X_i \in X|C_j \in C} = \frac{1}{|S_k|}\left[0 - \sum_{l=1}^{|S_k|}(Y_l : X_i = 0)\right]$$

$$\Rightarrow -(\gamma)_{X_i \in X|C_j \in C} = \frac{1}{|S_k|}\left[\sum_{l=1}^{|S_k|}(Y_l : X_i = 0)\right] \le 1$$

$$\Rightarrow -1 \le (\gamma)_{X_i \in X|C_j \in C}$$

$$\therefore -1 \le (\gamma)_{X_i \in X|C_j \in C} \le +1$$

$$and \ \ -1.44 \le (AR)_{X_i \in X|C^j \in C} = Log_2\left(e^{\gamma_{X_i}}\right)_{X_i \in X|C^j \in C} \le +1.44$$

In the input list, if a particular protein is essential (activator) for a particular cellular state, then its expression would be constitutively 1 in the set of all random sequences $S_K$. In that case, the numerator of the proportion $(\gamma)_{X_i \in X|C_j \in C}$ will be equal to $|S_k|$ and maximum and thus the value of the proportion will be equal to +1. Similarly, for a protein, which is inhibitor (or negative inducer) of a particular cellular state, it can be shown that the proportion will be equal to -1. The Log2 of the exponential of this proportion value is simply taken as scaling factor to stretch the distributions of the scores in the region of -1.44 to +1.44. This scaling is specifically useful for gaining high resolutions among the activity patterns of a large set of input proteins in the development of a particular cellular state. If AR score of a protein is either +1.44 or -1.44 in a given cellular state, then it can be said that the specific protein is highly essential and positive or negative inducer for that particular cellular state.

### *1.5 Calculation of phenotype cost function*

The emergence route of intra-tumor heterogeneity in GBM tumor cells from a common origin of mutated cells can be best analyzed with the help of tumor cell evolution phylogeny [22]. If the overall expressions of all the pathway molecules at $t^{th}$ time are considered as a 'state' of the cell $Z_t = \{M_t^1, M_t^2, M_t^3, ......M_t^m : m = Total\, molecules\}$, then the entire developmental routes through which different states $\{Z_t, Z_{t+1}, Z_{t+2}, ......Z_{t+T} : T = Total\, simulation\, time\}$ reach at the attractor states (singleton or periodic) are called as ‘State Transition Graph’ or STG [23]. The nodes of a STG are the states and edges are directed which defines the transition of one state to another state at every Boolean update. Hence, State Transition graph is able to depict the expression dynamics of all the pathway components and the transformation of the cells towards different cell types, starting from a common origin of tumor initiating cells (states). It is observed that for reaching at a particular attractor state, STG has to pass through few transition/intermediate states or nodes $\{S_t, S_{t+1}, S_{t+2}, ......\}$ and then land into either fixed-point node $\{S_{t+s}\}$ or cyclic attractor nodes $\{L_{t+1}, L_{t+2}, L_{t+3}, ......L_{t+l}\}$. It is considered that the transition from one state to another state of a cell is also associated with a signaling cost function (i.e. for chemical reactions/any physical processes) and it affects the overall state transition rate. Hence, it can be also assumed that the probability of a particular attractor/cellular state $(C_i)$ in equilibrium state starting from initial

states depends on the overall distance (i.e. transition steps) and the total number of molecular transitions to reach at that particular state. Hence, to quantify the average signaling cost functions $(\Phi_{C_i})$ in each simulation batch for each cellular state is considered to be the functions of total transitions steps and periodicity (in case of cyclic attractor), and the total number of molecular alterations performed starting from initial state to the attractor state. This function is defined in ***Eq. 6***.

$$\Phi_{C_i} = \frac{\phi(C_i)}{P_{C_i}} \tag{6}$$

$$\textit{Where, } \phi(C_i) = \frac{\sum_{t=0}^{s} d_{S_t \to S_{t+1}}}{s} + \frac{\sum_{t=0}^{l} d_{L_t \to L_{t+1}}}{l} \tag{7}$$

*Where,* $s = \textit{Total number of transition states require to reach at the steady state};$

$l = 1 \quad \textit{for Singleton attractor} \ \& \sum_{t=0}^{l} d_{L_t \to L_{t+1}} = 0$

$> 1$ *for Periodic attractor (= Periodicities of the cyclic attractor)*

$\mu = \textit{Total mutational costs of the entire model}$

$d_{S_t \to S_{t+1}}$ & $d_{L_t \to L_{t+1}}$ = *Hamming distance between two states in the transient and periodic paths*

$P_{C_i}$ =*Total number of occurrences of* $C_i$ *in a simulation batch*

Calculation of "Hamming distance" between two successive nodes (or cellular states) in STG is particularly interesting to know the temporal changes or evolution of the expression dynamics of the pathway molecules in the successive time points [24]. Let us assume that $S_t = \{X^1{}_t, X^2{}_t, X^3{}_t ......., X^N{}_t\}$ & $S_{t+1} = \{X^1{}_{t+1}, X^2{}_{t+1}, X^3{}_{t+1} ......., X^N{}_{t+1}\}$ are the expression vectors referring to the any arbitrary successive states in STG. The expression of the elements $(X^k{}_j)$ lies in binary domain i.e. $\{0, 1\}$. The Hamming distance ($\left| d_{S_j \to S_{j+1}} \right|$) can be calculated by using the following ***Eq. 8***.

$$\left| d_{S_j \to S_{j+1}} \right| = \sum_{k=1}^{N} (X^k{}_{j+1} - X^k{}_j) \tag{8}$$

Moreover it is also assumed that, apart from the signaling cost a mutated model in which few molecules are constitutively activated/suppressed will also induce mutational cost $(\Omega)$ in the cells at the time of its transitions. The mutational cost function for a specific model is dependent on the total number of induced mutations $(\mu)$ and the change in the total number of added and omitted cellular states in the mutated model with respect to the non-mutated model $(\mu = 0)$.

Let us consider, in the non-mutated model there are total maximum X number of Cellular states $(C^n : C_1^n, C_2^n, C_3^n, ......C_X^n)$ observed and in the mutated model there are total maximum Y number of Cellular states $(C^m : C_1^m, C_2^m, C_3^m, ......C_X^m)$ observed. Suppose in the mutated model there are total $\mu$ mutations induced. Hence, the mutational cost $(\Omega)$ is defined as follows (***Eq. 9***).

$$\Omega = \mu \times \frac{|C^n - (C^n \cap C^m)| + |C^m - (C^n \cap C^m)|}{\left(\left|C^n \cup C^m\right|\right)} \quad (9)$$

$\mu = Total\ induced\ mutations\ in\ the\ mutated\ model$

$\left|C^n \cup C^m\right| = Total\ number\ of\ cellular\ states$

$|C^n - (C^n \cap C^m)| = Total\ number\ of\ cellular\ states\ which\ are\ not\ appeared\ in\ the\ mutated\ model$

$|C^m - (C^n \cap C^m)| = Total\ number\ of\ cellular\ states\ which\ are\ newly\ appeared\ in\ the\ mutated\ model$

Hence, the phenotype cost function $(\Psi_{C_i})$ is defined as the sum of total signal cost and the mutational cost (if any) for a particular state $(C_i)$ in the STG of a Boolean model. It is defined as follows (***Eq. 10***).

$$\Psi_{C_i} = \Phi_{C_i} + \Omega \quad (10)$$

***Phenotype Predictor Score:*** The predictor score $[\Theta(C_i)]$ of a particular cellular state observed in the simulation defines as the ratio of the total number of observed occurrences $(P_{C_i})$ to the total cost $(\Psi_{C_i})$ requires for reaching at ***i^th^*** cellular state $(C_i)$ in the attractor space. Hence, this parameter can be defined as follows (***Eq. 11***).

$$\Theta(C_i) = \frac{E(P_{C_i})}{E(\Psi_{C_i})} \quad (11)$$

$Where,\ E(P_{C_i})\ \&\ E(\Psi_{C_i}) = Expected\ values\ of\ P_{C_i}\ and\ \Psi_{C_i}\ out\ of\ N\ number\ of\ simulation\ batches$

The probability density of the phenotype predictor score $(\Theta = \frac{P}{\Psi})$ is previously discussed in various literatures [25,26], where $P \sim \mathrm{N}(\mu_P, \sigma_P^2)$, $\Psi \sim \mathrm{N}(\mu_\Psi, \sigma_\Psi^2)$, $\rho = Corr(P, \Psi) \neq \pm 1$, and the probability density function $f(\Theta)$ is given as follows (***Eq. 12***).

$$f(\Theta) = \frac{\sigma_P \sigma_\Psi \sqrt{1-\rho^2}}{\pi\left(\sigma_\Psi{}^2\Theta^2 - 2\rho\sigma_P\sigma_\Psi\Theta\right)}\left[\exp\left(-\frac{1}{2}SupR^2\right) + \sqrt{2\pi}.R.\Phi(R).\exp\left(-\frac{1}{2}[\sup R^2 - R^2]\right)\right] \quad (12)$$

$$Where, R = R(\Theta) = \frac{\left(\frac{\mu_P}{\sigma_P} - \rho\frac{\mu_\Psi}{\sigma_\Psi}\right)\Theta - \left(\rho\frac{\mu_P}{\sigma_P} - \frac{\mu_\Psi}{\sigma_\Psi}\right)\frac{\sigma_P}{\sigma_\Psi}}{\sqrt{1-\rho^2}\sqrt{\Theta^2 - 2\rho\frac{\sigma_P}{\sigma_\Psi}\Theta + \left(\frac{\sigma_P}{\sigma_\Psi}\right)^2}}$$

$$SupR^2 = \frac{\left(\frac{\mu_P}{\sigma_P}\right)^2 - 2\rho\frac{\mu_P}{\sigma_P}\frac{\mu_\Psi}{\sigma_\Psi} + \left(\frac{\mu_\Psi}{\sigma_\Psi}\right)^2}{1-\rho^2}$$

$$SupR^2 - R^2 = \frac{\left(\frac{\mu_P}{\sigma_P}\frac{\sigma_P}{\sigma_\Psi} - \frac{\mu_\Psi}{\sigma_\Psi}\Theta\right)^2}{\left(\Theta^2 - 2\rho\frac{\sigma_P}{\sigma_\Psi}\Theta + \left(\frac{\sigma_P}{\sigma_\Psi}\right)^2\right)}$$

$$\Phi(R) = \frac{1}{2}erf\left(\frac{R}{\sqrt{2}}\right)$$

The function $f(\Theta)$ follows the general properties of the Cauchy-like distribution [25].

# 2 Model constructions and validation

### *2.1 Exploration of maximum number of attractor states using Shannon entropy in aNSC model.*

In the aNSC model simulation, the expected normalized frequencies and distributions of the cellular states under the steady state situation of each simulation batch are found to be highly uncertain. Hence, by considering all the distinct cellular states appeared in each simulation batch as "events", Shannon entropy scores are calculated for every batch (Supplementary Fig. 1A). The maximum information or Shannon entropy score, 1.456 Shannon, is achieved in the batch number 21, which is subsequently opted for further analyses. Selection of this simulation batch with highest information entropy strongly asserts that the probability of obtaining maximum number of attractor or cellular states with highest normalized frequency is maximum (using ***Theorem 1***), indicating that the 10,000 random initial states used in each batch are sufficient enough to explore maximum number possible attractor states of the model.

### *2.2 Developmental model captures the emergence of cellular heterogeneity in the aNSCs niche.*

Total 8 different cellular states (i.e. phenotypically distinct, heterogeneous cell types) obtained, out of which 3 cellular states (*viz.* Quiescent, Apoptosis and Neuron Progenitor Cells (NPC) Differentiation) belong to fixed-point (singleton) attractor states referring either the fully differentiated (matured), inactive (i.e. quiescent) or dead cells (i.e. apoptotic) (Supplementary Fig. 1B). The remaining 5 cellular states (*viz.* NSC Renewal/NPC Differentiation/Apoptosis, NSC Renewal/NPC Differentiation, NSC Renewal/Apoptosis, Astrocyte Progenitor Cell or ASPC Differentiation, and Glioblastoma Stem Cell or GSC Renewal) belong to cyclic (periodic) attractor space and are mainly corresponding to the periodic behavior of cell cycle divisions (i.e. proliferation, growth) followed by differentiation or apoptosis (Supplementary Fig. 1B). However, unlike the differentiation of neural progenitor cells (NPCs) which is appeared in both the singleton (i.e. NPC differentiation) and periodic (i.e. NSC/NPC, NSC/NPC/Apoptosis) attractor states, differentiation of astrocytes ASPCs are only found in the cyclic attractor states. Finally, appearances of these varieties types of cellular states in the steady states level (or attractor space) are able to improve the potential of the developed aNSC model to capture the coexistence

of heterogeneous cells in the microenvironment of aNSCs, which consists of quiescent, apoptotic, stem-like as well as differentiated neuronal and astrocytes cells.

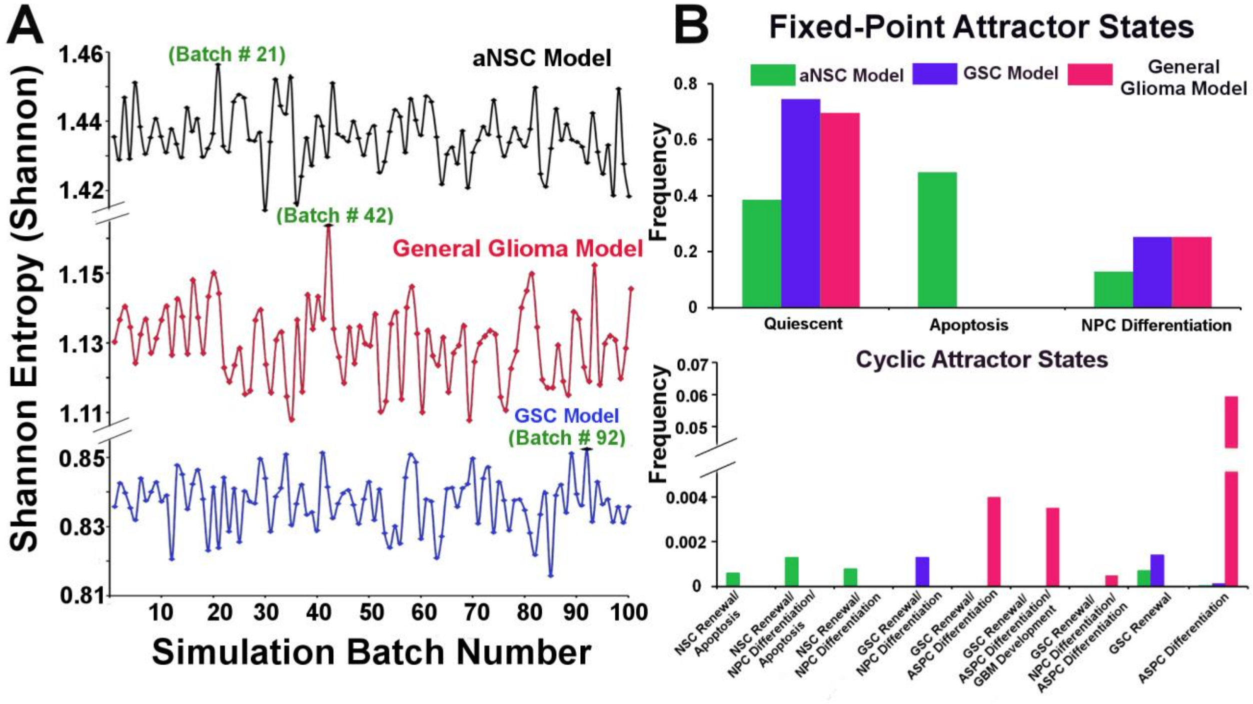


**Supplementary Fig. 1. Simulation results of the aNSC, GSC, and General glioma models.** (A) Shannon entropy scores observed, and (B) Normalized frequencies of cellular states observed in aNSC, GSC, and General glioma model simulations. Batch numbers 21, 92 and 42 in aNSC, GSC, and General glioma models with highest Shannon entropy scores in each model simulations, respectively, are chosen for calculating the normalized frequencies of each attractor states of different models.

### *2.3 aNSCs are highly biased towards the development of quiescent, neural progenitor and apoptotic states*

It is experimentally proven that during the neurogenesis, around half of the neurons undergo apoptosis to keep the neuron cell population at optimum level [27-30]. Previous experiment, performed on murine pluripotent PCC7-Mz1 neural stem cells, has shown that approximately 25% of NSCs die by apoptosis within 24 hours from the onset of neurogenesis [31]. During neurogenesis, aNSCs differentiate into more number of neurons and initially make maximum number of connections with each other to establish a dense neuronal connections in the brain [31]. However, after reaching the maximum connectivity, these excessive connections start reducing as most of the neurons (> 50%) undergo apoptotic process and eliminated from the neurogenic niche [31]. Hence, compiling these experimental observations, it can be stated that the higher rate of apoptosis exists during the course of neuronal development in the adult brain tissue. Further analyses of our simulation results have revealed that the normalized frequencies of singleton attractor or cellular states are comparably higher than the periodic attractor states (Supplementary Fig. 1B), and the singleton attractor state "Apoptosis" has highest normalized frequency (Mean

0.494 ± 0.005 S.D.) compare to all others cellular states, thus supporting the experimental observations.

The normalized frequency of the undifferentiated "Quiescent state" (Mean 0.378 ± 0.005 S.D.) is the next highest cellular state after Apoptosis (Supplementary Fig. 1B), justifying the previous experimental results, where a significant large quantity of quiescent neural stem cells (qNSCs) in the neurogenic niche [20,32] is observed. Complete differentiation of aNSCs into matured neurons (i.e. NPC Differentiation state) is also observed in this simulation within the singleton attractor state. The normalized frequency (Mean 0.125 ± 0.003 S.D.) of this cellular state observed in the simulation outcomes indicates the natural bias of aNSCs to the differentiation of matured neuron. In contrasts, normalized frequency of normal ASPC differentiation state, obtained within periodic attractor space, is observed to be very less (Mean 0.00023 ± 0.00014 S.D.) and can be designated as a non-biased event during neurogenesis (Supplementary Fig. 1B). Hence, it can be concluded that the inertia in the internal circuitry of aNSCs drives the stem cells to opt neuronal cell fate decision over the normal ASPC differentiation process. Similarly, it is also observed that the normalized frequencies of other cyclic cellular states associated with the periodic attractors are also very less as compare to the singleton attractor states (Supplementary Fig. 1B).

It is also observed that all the periodic cellular states mapped with the self-renewal process of aNSCs followed by apoptosis, NPC differentiation or both (*viz.* NSC Renewal/Apoptosis, NSC Renewal/NPC Differentiation, NSC Renewal/NPC Differentiation/Apoptosis) have similar normalized frequency values (Supplementary Fig. 1B). These comparative analyses of cyclic cellular states strongly establish the earlier observations of the general tendency of aNSCs to maintain either of its stemness property or neuronal cell fate specification over the differentiation of astrocytes [33]. Specification of these cell lineages at the time of aNSCs development are found to be associated with the expression profiles of the marker proteins (Supplementary Table 3). In depth analyses are further performed to distinguish the cell lineages by assessing expression dynamics of these marker proteins. The state transition dynamics of these observed cellular states and the temporal expression patterns of their corresponding marker proteins are shown in Supplementary Figs 2 and 3.

### *2.4 Application of Activity Ratio scores for the extraction of driver proteins of different cellular states*

In order to identify the driver genes/proteins of a particular cellular state, the input molecules having optimum "Activity Ratio" score (-1.44 ≤ AR Score ≤ +1.44) are extracted in each cellular state through comparative analysis (Fig. 2A). It has correctly identified the important proteins, driving the dynamics of apoptosis, self-renewal and differentiation of adult NSCs [34]. Also, the hierarchical clustering of the AR scores of all the cellular states shows separate clusters of fixed-point and cyclic attractor states. It is observed that EP300, RBPJ, APH1A, NCSTN, PSENEN, SNW1, HAT1, and MAML1 are highly expressed (i.e. maximum AR scores) in all the cyclic attractor states. These proteins are the core components of Notch pathway, which maintain the balance between the self-renewal and differentiations of aNSCs by helping transcriptions of the target genes (HES 1-7/HEY 1, 2, L) [6,19,35,36]. Higher AR scores of all of these proteins are found in the self-renewing or cell division cycles (i.e. aNSC renewal state), whereas lower scores of canonical (i.e. core components) and non-canonical (i.e., DTX1) components are observed in

neuronal (NPC) differentiation process [19]. On the other hand, higher AR scores of all the active Notch pathway components with JAK2/STAT3 proteins are detected in astrocytes (ASPC) differentiation process [19,37]. The novel scoring method, AR score is correctly predicted the proteins implicated in different sub-types of brain cells and thus justify its application for the identification of driver proteins behind the development of a particular phenotype or cellular state.

Identification of the driver proteins in Notch signaling, also help to establish the role of Notch pathway as the guardian behind the maintenance of stem-like properties of adult NSCs and the suppressor of neuronal differentiation process thorough this *in-silico* approach. Follow up analyses reveal the active regulatory motifs within the pathway, which can shift the stem-like properties of aNSCs towards either differentiation (neuron or astrocyte) or apoptotic process (Supplementary Fig. 5).

### *2.5 P53 mutation does not affect the differentiation potential of Glioblastoma stem cells*

Low AR score of P53 protein in GSC renewal state observed in aNSC model simulation (Fig. 1A) justify the role of this protein in the development of GBM tumor initiating stem cells [27,28,32]. Hence, to further verify the knock-out effect of this protein in the self-renewal of aNSC cells and the origin of evolution of Glioblastoma stem cells (GSCs), a new GSC developmental model is build by keeping P53 at down-regulated state (i.e. total mutations, $\mu = 1$). This model is particularly developed to analyze the dynamic interplay of P53 and Notch pathway to trigger the development of GSCs. Simulation batch number 92 has highest Shannon entropy score (0.852 Shannon), which is comparably much lesser than the observed entropy (1.456 Shannon) found in aNSC model (Supplementary Fig. 1A). In total five attractor states obtained in this simulation *viz.* i) Quiescent, ii) NPC Differentiation, iii) GSC Renewal iv) GSC Renewal/NPC Differentiation, and v) ASPC Differentiation (Supplementary Fig. 1B). As expected, the simulation outcomes show none of the GSC cells (i.e. GSC renewal state) are undergoing towards the apoptotic state and the normalized frequency (0.0014) is increased 2-folds as compare to the normalized frequency (0.0007) observed in aNSC model

The simulation results clearly show that the P53 knocked-out GSCs are still capable of producing differentiated neurons and astrocytes cells (Supplementary Fig. 1B). Like aNSC model simulation, the normalized frequency of "NPC Differentiation" is found to be much higher (0.2516) than the "ASPC differentiation" state in this model. This result establishes the previous findings that wild-type (i.e. aNSC) and P53 knocked-out GSCs, both are capable differentiation, but the neuronal differentiation is mostly preferred over the astrocytes differentiation [30,32,38].

### *2.6 The nexus of YY1 transcription network and core Notch signaling induces tumorigenesis in GSCs*

Highest values of the core components of Notch signaling, JAK2, STAT3, and CSL/RBPJ and lowest value of P53 proteins in the AR score distribution of mutated ASPC differentiation state is observed in GSC model (Fig. 1A-1B). Hence, to further verify the influences of these proteins in the development of astrocytoma, JAK2, STAT3, RBPJ proteins are kept activated P53 protein is kept down-regulated in a new simulation, and thus a new model of general Glioblastoma (GBM) development (total mutations, $\mu = 4$) is developed. The Shannon entropy information (1.164 Shannon) is found higher than the GSC model (Supplementary Fig. 1A), which signifies the number of attractor states expected in General glioma model is comparatively

higher. There are maximum six different (two fixed-point and four cyclic) attractor states possible to be emerged from this mutated system in presence of random fluctuations of input proteins (Supplementary Fig. 1B). Attractor states, i) Quiescent, and ii) NPC differentiation are obtained in the fixed-point steady states with higher normalized frequencies, whereas the cyclic attractors states: i) ASPC Differentiation, ii) GSC Renewal/ASPC Differentiation, iii) GSC Renewal/ASPC Differentiation/GBM Development, and iv) GSC Renewal/ASPC Differentiation/NPC Differentiation are all found in comparatively lower normalized frequency ranges. Out of all these cellular states/attractors, the cyclic attractors, *viz.* ii), iii) and iv), are the newer states appeared in this mutated system. This indicates that cross-talks of over-activated JAK2/STAT3 pathway and deregulated P53 pathway with actively functioning Notch pathway have the potential to shift the developmental dynamics of GSCs towards the tumorigenic states, which correspond to different sub-types of GBM.

The normalized frequency of ASPC differentiation state is comparably much higher than all other cellular states belonged to the cyclic attractor space (Supplementary Fig. 1B). This state denotes the uncontrolled cell division (without apoptosis) and differentiation of astrocytes in the neurogenic niche without the expressions of high-grade GBM markers (e.g. MYC, TENASCIN-C) and thus it is defined as "Low grade glioma (LGG-I)" state (Supplementary Fig. 4). Similarly, another cellular state "GSC Renewal/ASPC Differentiation" is also categorized in the similar LGG group of tumor cells, but unlike the LGG-I grade, it constitutively expresses the oncoprotein TENASCIN-C and simultaneously expresses the markers of proliferating GSCs (Supplementary Fig. 4). Hence, due to the presence of high cell proliferation rate and GSCs, this state would have higher lethality, poor prognosis and higher aggressive in nature as compared to the cells belong to LGG-I grade, and therefore it is further classified as LGG-II grade tumor. Another cellular state, which has very lower normalized frequency as compare to the other two grades (LGG-I/II), is "GSC Renewal/ASPC Differentiation/GBM Development" (Supplementary Fig. 1B). The phenotypic characteristics associated with this cellular state are the self-renewal of GSCs, differentiation of mutated ASPCs, and the development of high-grade GBM (HGG) markers (Supplementary Fig. 4). The distinct molecular profiles of the marker proteins observed in this state are *viz.* constitutive over-expression of C-MYC and TENASCIN-C proteins, longer time period of GFAP expression dynamics including the expression of Cyclin-D1 and D3. These phenotypic and molecular characteristics associated with this cellular state are highly correlated with the development of high grade (i.e. Grade-IV) Glioblastoma tumor [39-42] state.

Highest AR score of the transcription factor YY1 in high-grade GBM state points out the possible cross-talks of this protein with JAK2, STAT3, RBPJ, and P53 (Fig. 1C). Previous report suggests that YY1 interacts with the ankyrin domain of Notch intracellular domain (NICD) and modulates the expression of notch target genes [43]. Later, it is proven that non-canonical Notch pathway targets proto-oncogene *C-MYC* expression with the help of transcription factor YY1 [44]. It is also proven that *C-MYC* and its downstream target genes are significantly over-expressed in high-grade GBM cells [45]. Positive correlation between the over-expression of YY1 and the progression of high-grade gliomas and meningliomas is also found in the later experiment [46]. Therefore, compiling these experimental data, it can be stated that cross talks between the YY1 transcription regulatory network and core Notch signaling network are positive inducers of high-grade GBM tumor growth.

# 3 Data Analyses

## *3.1 Temporal variations of marker proteins in aNSC, GSC and General Glioma Models*

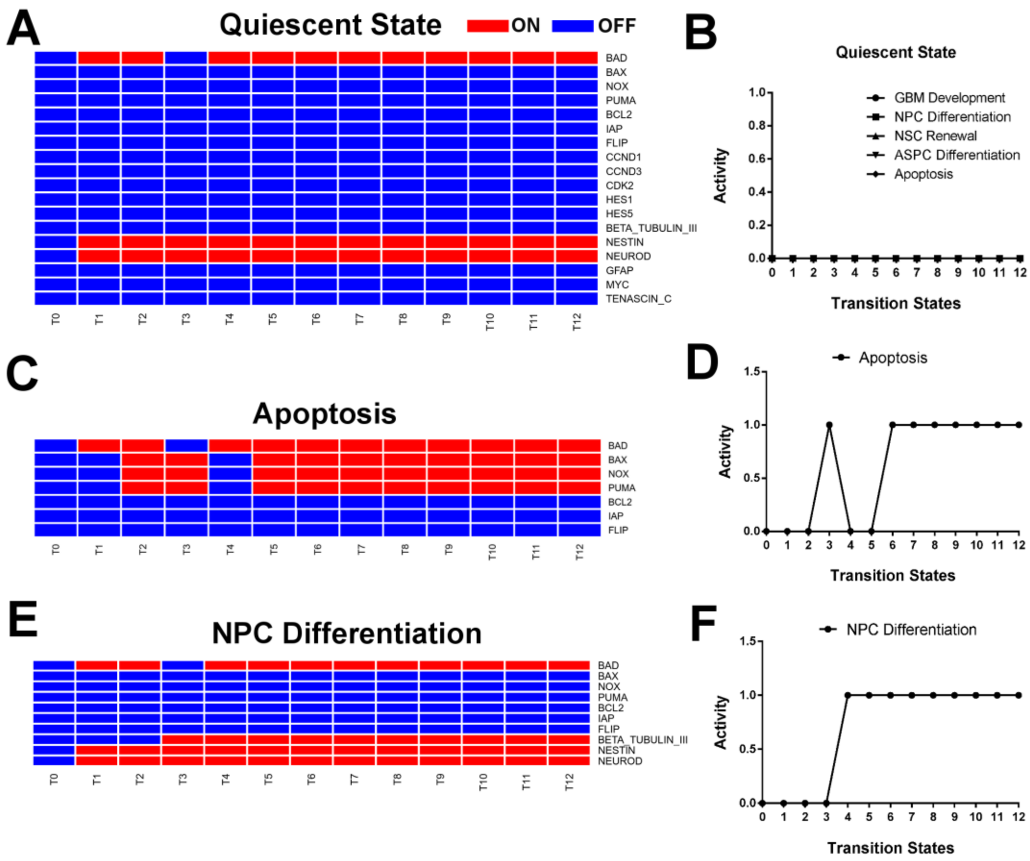


**Supplementary Fig. 2. Activity profiles of different markers proteins and the temporal dynamics of the corresponding fixed-point attractor states observed in aNSC simulation.** (A) & (B) Quiescent state; (C) & (D) Apoptosis; (E) & (F) NPC Differentiation.

In the activity profiles (Supplementary Fig. 2), only the important marker proteins corresponding to different cellular phenotypes considered in the aNSC model are depicted. It is identified that in the quiescent state, none of the cellular phenotypes are found in the active/ON state (Supplementary Fig. 2B). However, in the saturation time-points of quiescent state, the neuronal markers e.g. NESTIN and NEUROD are expressed, but another most important neuronal marker β-Tubulin-III is found in inactive/OFF state. Due to these characteristics, such attractor state or cellular phenotype can be considered as "quiescent" neural stem cells (or 'qNSCs'). These cells are kept in the neurogenic niche of SVZ with higher population (or normalized frequencies) and get reactivated when proper conditions arrive [47]. qNSCs work as constant source for the production of pluripotent, adult neural stem cells (aNSCs), which finally committed to generate the differentiated, matured brain cells such as neurons, astrocyte, oligodendrocyte etc. after receiving proper signals [20,47]. One of such differentiated state which is also observed in the simulation, is "NPC differentiation", in which all the neuronal markers including β-Tubulin-III are found to be expressed at a steady state active level (i.e. ON state) and the entire process reaches to

the homeostasis [48]. Constitutive expressions of NESTIN and β-Tubulin-III proteins are commonly found in the neural progenitor cells committed to form matured neurons in neurogenic niche [20].

On the other hand, one of the attractor states of aNSC model is detected to reach at apoptotic cell death process if the pro-apoptotic markers such as PUMA, NOX, BAX, and BAD are constitutively over-expressed in the qNSCs (Supplementary Fig. 2C, 2D). Steady state over-expressions of these pro-apoptotic proteins trigger the apoptotic pathways by regulating their downstream effectors and thus direct the qNSCs/aNSCs or the matured cells towards the programmed cell death [8]. Simulation result reveals that expressions of these pro-apoptotic marker proteins within NSCs in the absence of appropriate Notch pathway activation have the potential to kill the stem cells and eliminate them from the neurogenic niche of SVZ. However, the deficiencies in the population of stem cells induced by the natural cell death are replenished by reactivating the quiescent NSCs present in the niche by triggering and directing the intracellular reaction cascades such as Notch at the appropriate time [49].

Apart from these above mentioned fixed-point attractor states, there are other five cyclic attractor states found in the aNSC model simulation. The expression profiles of the relevant markers and the activities of these attractor states are also analyzed to understand the involvement of Notch pathway in the regulation of their dynamics within NSCs (Supplementary Fig. 3). Periodic expression patterns of all the stem cell renewal/proliferation markers (i.e. HES1, HES5, CCND1, CCND3, and CDK2) throughout the state transition dynamics are observed within all of these cyclic attractor states. Periodic expressions of the cell proliferation markers CCND1, CCND3 and CDK (i.e. cyclic expressions of Cyclins and CDK complex) are the main regulator of cell cycle progression [50]. The variations of the expressions of other marker proteins are also observed in different cellular states featuring different phenotypic characteristics. For example, it is observed that along with these stem cell marker proteins, the markers responsible for neuron progenitor cells (e.g. NESTIN, NEUROD, and β-Tubulin-III) and pro-apoptosis factors (e.g. PUMA, NOX, BAX etc.) are also expressed in few attractor states. In such cases, the individual phenotypes NSC renewal, NPC differentiation and apoptosis processes oscillate systematically throughout the transition time of Boolean update. Expressions of such multiple markers (e.g. stem cell markers with the marker proteins of neuronal cells) lead the phenotypic characters of the Notch activated cells towards a complex cellular state (e.g. "NSC Renewal/ NPC Differentiation/ Apoptosis") in which the different marker proteins are found to be co-expressed or sequentially expressed (Supplementary Fig. 3A). Interestingly, while analyzing the cyclic steady states of these phenotypes in this cellular state, it is observed that the oscillation (or phase) of the phenotype NSC Renewal is leading followed by the NPC Differentiation and Apoptosis (Supplementary Fig. 3B). This observation clearly demonstrates the transitions of the cycles of neural stem cell renewal to the differentiation of neuron and its apoptosis in the neurogenic niche. In another cellular state, the phenotypic markers for NPC differentiation are not found to be active, but the markers for NSC renewal and Apoptosis are found in active state. Hence, this complex state ("NSC Renewal/ Apoptosis") in the simulation demonstrates the cyclic expression of NSC self-renewal process of NSCs followed by its apoptosis (Supplementary Fig. 2C, 2D).

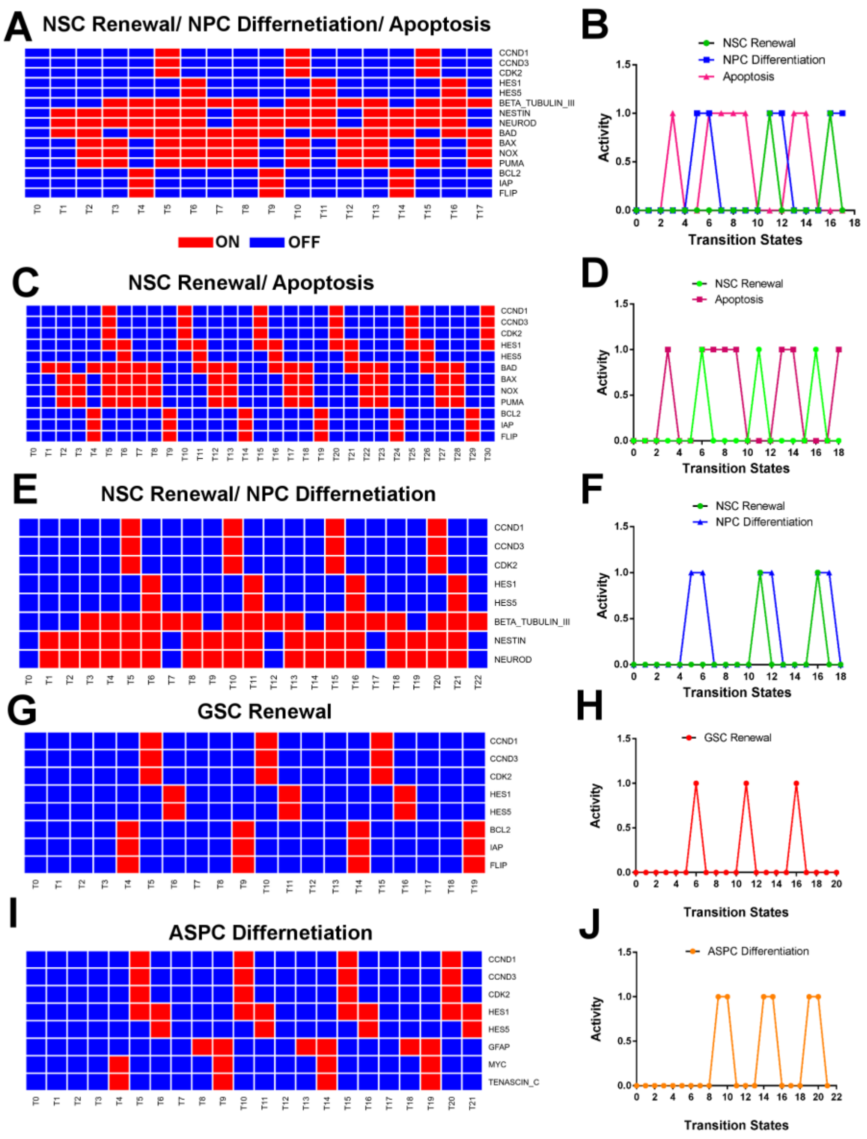


**Supplementary Fig. 3. Activity profiles of different markers proteins and the temporal dynamics of the corresponding cyclic attractor states observed in aNSC simulation.** (A) & (B) NSC Renewal/NPC Differentiation/Apoptosis; (C) & (D) NSC Renewal/Apoptosis; (E) & (F) NSC Renewal/NPC Differentiation; (G) & (H) GSC Renewal; (I) & (J) ASPC Differentiation.

On the other hand, it is observed that cyclic expressions of cell proliferation and stem cell markers with the expressions of anti-apoptotic factors (e.g. BCL2, IAP, FLIP etc.) can lead the cellular phenotype towards uncontrolled proliferation without apoptotic break, which is denoted here as "GSC Renewal" state (Supplementary Fig. 3G, 3H). Experimental observations and the simulation outcomes of this study, both are confirming the phenomena of the developmental dynamics of GSCs from aNSCs within the neurogenic niche of SVZ. Also, it is identified that deregulation in Notch pathway and the over-expression of anti-apoptotic factors along with P53 mutation are one of the major causes behind the generation of tumorigenic stem cells (GSCs) in the brain tissue. Similarly, the cyclic oscillations of astrocyte marker GFAP along with the stem

cell proliferation markers are found active in the "ASPC Differentiation" state (Supplementary Fig. 3I, 3J).

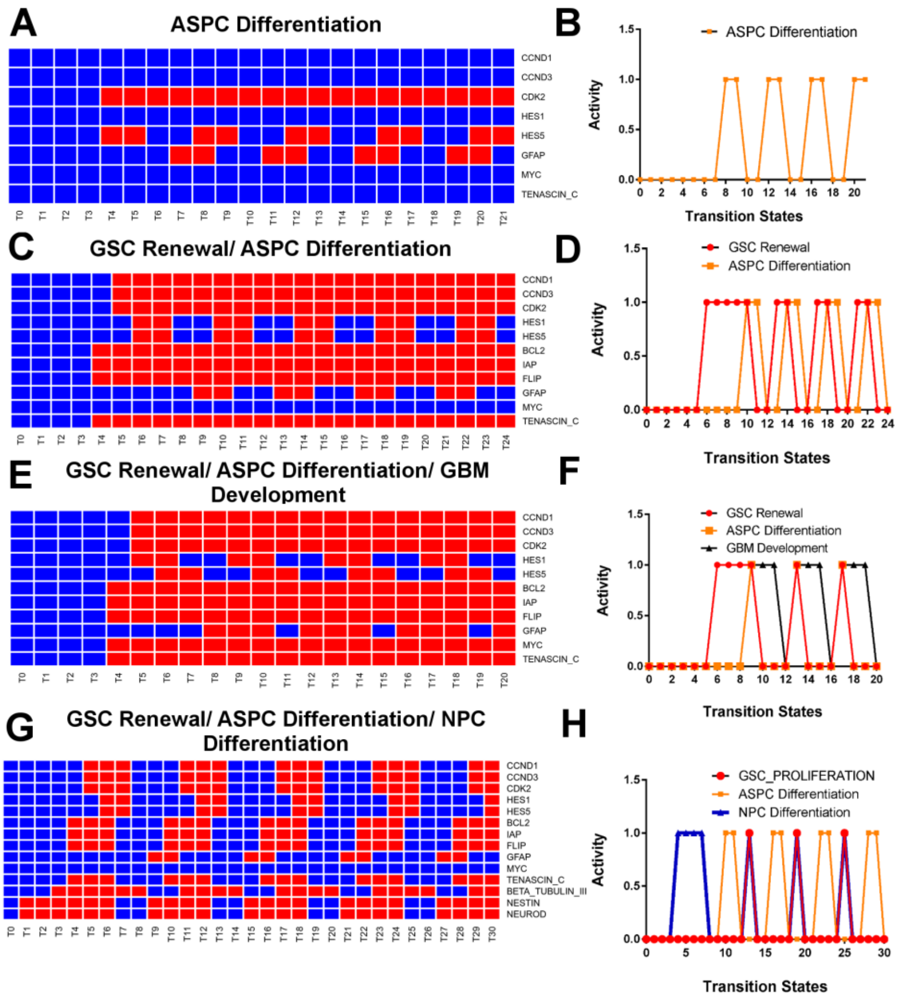


**Supplementary Fig. 4. Activity profiles of different marker proteins and the temporal dynamics of the corresponding cyclic attractor states observed in general glioma model simulation.** (A) & (B) GSC Renewal/ASPC Differentiation; (C) & (D) GSC Renewal/ASPC Differentiation/ GBM Development; (E) & (F) GSC Renewal/ASPC Differentiation/NPC Differentiation.

Similar to the aNSC model, the simulation results of "GSC model" (in which P53 is constitutively knocked-out) also predict the outcomes of different cellular states/phenotypes, which are closely same as the aNSC model simulation. The observed cellular sates (fixed-point and cyclic) of GSC model *viz.* Quiescent state, NPC Differentiation, GSC Renewal, ASPC Differentiation are all having similar expression patterns (data not shown) of their marker proteins as compare to the results observed in aNSC model simulation (Supplementary Figs 2 and 3).

On the other hand, while simulating the general glioma model (in which P53 is inactive and JAK2, STAT3, and RBPJ are constitutively over-expressed), another three new cyclic attractors

(or cellular states viz. i) GSC Renewal/ASPC Differentiation, ii) GSC Renewal/ASPC Differentiation/GBM Development, and iii) GSC Renewal/ASPC Differentiation/NPC Differentiation) are found to be emerged in the attractor space with varying normalized frequency distributions (Supplementary Fig. 4). Fixed-point attractor states *viz.* Quiescent and NPC differentiation and the cyclic attractor states *viz.* ASPC differentiation are also observed in this model simulation as observed in aNSC and GSC models. The cellular state ASPC Differentiation (with $P53^{-/-}$, $JAK2^{+/+}$, $STAT3^{+/+}$, $RBPJ^{+/+}$ mutations) observed in this model simulation is an oscillating attractor state with the expression of GFAP protein, uncontrolled proliferation or division of mutated astrocyte cells and no apoptotic break. Hence, the phenotypic characteristic of this cellular state is tumorigenic in nature and thus it is denoted as "Low grade glioma (LGG-I)" state (Supplementary Fig. 4A, 4B).

Moreover, the protein activity profile observed in another cellular state: "GSC Renewal/ASPC Differentiation" reveals that the expression of the astrocyte differentiation marker GFAP is oscillating along with the stem cell markers (tumor) and the anti-apoptotic markers are constitutively expressed, which also make this cellular state tumorigenic in nature (Supplementary Fig. 4C). It is observed that both the phenotypes GSC renewal and ASPC differentiation processes are oscillating with fixed time periods throughout the transition time points around their respective steady state levels. Both the phenotypes have same periodicities (i.e. 4 units of transition states) and out of these two cellular states, GSC renewal process is moving ahead from the ASPC differentiation process. This observation clearly indicates the real biological scenario, in which self-renewal/proliferations of GSCs are initiated in the brain tissue followed by its differentiation and maturation into ASPCs (Supplementary Fig. 4D). It also proves the role of Notch pathway to maintain the inertia of GSCs at self-renewal state compare to differentiation and maturation into glial cells and also demonstrates the slow rate of differentiation of mutated and proliferating astrocytes/glial cells from GSCs in the tumor niche. It should be noted that the ASPCs generated though this process are purely tumorigenic in nature with multiple oncogenic mutations and uncontrolled rate of proliferation without apoptosis. However, the genotypic signature of ASPCs found in this cellular state have down-regulated C-MYC (a marker for high-grade GBM), which in turn makes this cellular state less tumorigenic (i.e. low grade) in nature as compare to grade-IV glioblastoma tumor cells. As this cellular state contains both tumorigenic stem cells (GSCs) and mutated astrocytes (ASPCs), hence this state is defined here as the "LGG-II" state.

Apart from these two major cellular states, the state transition dynamics of the model also leads towards the two other complex attractor states, which are phenotypically mapped with the "GSC Renewal/ASPC Differentiation/GBM Development" and "GSC Renewal/ASPC Differentiation/NPC Differentiation" cellular states. In the first one, it has shown the cyclic expression patterns of all the GSCs self-proliferation, ASPC maturation or differentiation and simultaneously the constitutive over expressions of important high-grade glioblastoma marker proteins C-MYC and TENASCIN-C (Supplementary Fig. 4E). Although both the processes, involved in GSC renewal and ASPC differentiation, are showing oscillatory dynamics around the steady state time points, but the observed periodicities of both the cellular states (i.e. 3 units of transition states) are lesser than the previously mentioned LGG/GSC cellular state. More importantly, in this cellular state both the phenotypes are oscillating synchronously and dividing rapidly in the tumor niche, which will eventually accelerate the entire population of the mutated astrocytes to grow exponentially without any apoptosis (Supplementary Fig. 4F). Interestingly, it

is observed that if the malignant GBM markers C-MYC and TENASCIN-C, both are expressed in the rapidly proliferating ASPCs, then the cumulative dynamics of the entire cellular state would transform more aggressively into the self-proliferating stage. Hence, based on these phenotypic and genotypic characteristics, this particular cellular state observed in this simulation is further denoted as "High-Grade GBM" state. The characteristic dynamics of GBM developmental process is also periodic in nature, but the periodicity and the amplitude are comparably higher than the GSC renewal and ASPC differentiation processes.

### *3.2 Network motifs of Notch pathway in the regulation of different cellular states*

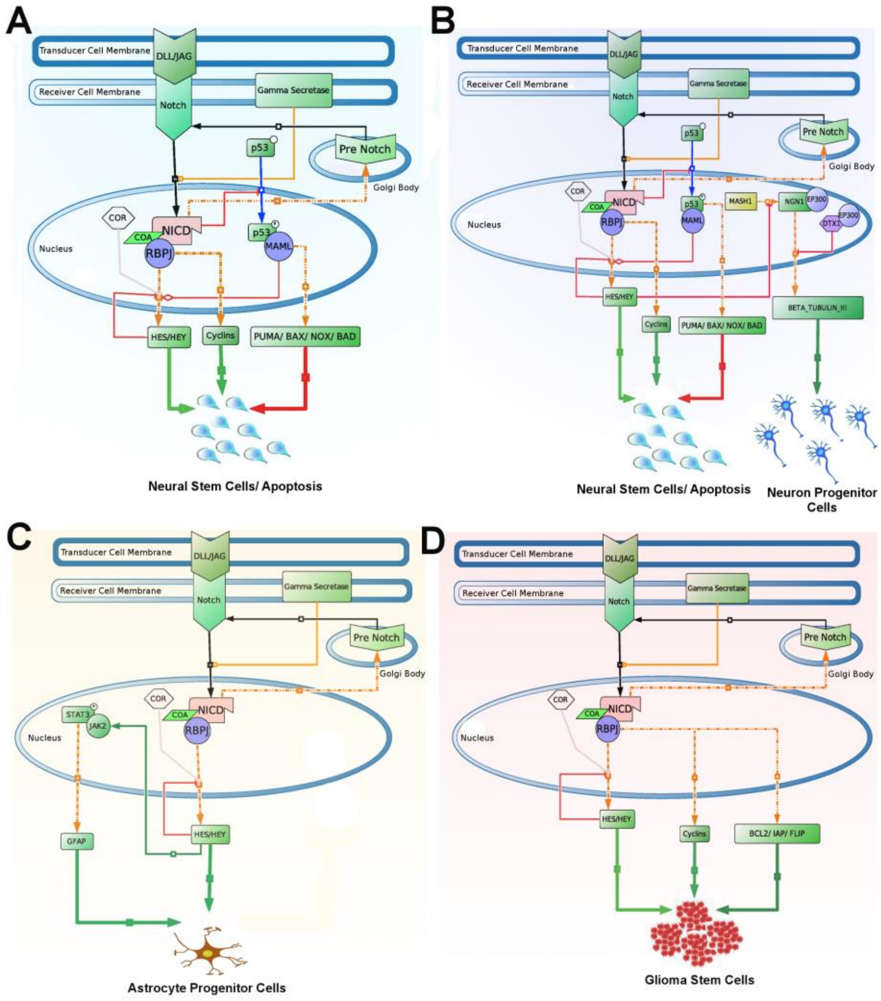


**Supplementary Fig. 5. Active network modules in Notch pathway associated with various cellular states.** (A) NSC Renewal/ Apoptosis; (B) NSC Renewal/NPC Differentiation/Apoptosis; (C) ASPC Differentiation; and (D) GSC Renewal. COA and COR represent co-activator and co-repressor complex, which are the important gene regulatory components for regulating the expression of Notch target genes such as *HES1/5*, *HEY*, *CYCLIN-D1* etc.

It is observed that, in the normal situation, when the core Notch pathway is active and the P53 protein is not mutated in the aNSCs, the target proteins (*HES1*, *HES5* etc.) produced at the end of this pathway are normally expressed and follow cyclic temporal expression pattern (results not

shown) [51]. It is experimentally proven and also shown in the identified network motif that the transcription complex (NICD:CSL:MAML) of Notch pathway produces the precursor of NOTCH receptor protein [1], which is then turns to post-processing (most often glycosylated) and transferred to the cell membrane, where it further binds to the Notch pathway activation ligands Delta/Jagged (Supplementary Fig. 5A). Thus, this reaction pathway makes a positive feedback loop in the reaction motif and triggers oscillatory dynamics of the Notch target genes *HES1, HES5, CYCLIN-D1, CYCLIN-D3* etc. The entire reaction motif works in such a way that it enforces the transcription regulatory network of notch pathway to produce the cell cycle regulatory proteins CYCLIN-D1, CYCLIN-D3 etc. in periodic pattern. In the previous experiments and current simulation studies, oscillations in the dynamic expressions of HES1, HES5 genes/proteins are also observed [52]. However, the expressions of HES1/HES5 proteins negatively influence the transcriptions of their own expressions and thus create a negative feedback loop in the motif [53]. The periodic expressions of CYCLINS and CDK proteins, a well known indicator of cell proliferation process, are also observed due to the presence of these feedback loops in the simulations [42]. It is also observed from the simulations that the controlled regulation and fine - tuning of this positive and negative feedback loops are the main regulators behind the maintenance of stem cell renewal and proliferation process. Further, perturbation studies performed by eliminating or targeting any of this feedback loops would shift the cellular dynamics towards different cellular states (neurogenesis or gliogenesis).

In another reaction motif, it is observed that when the expressed HES1/HES5 proteins, which are also known as bHLH transcription repressor protein, interact with the transcription regulatory network of *MASH1* gene, then the entire reaction motif is redirected to suppress the expressions of neuronal cell markers e.g. NGN1, β-TUBULIN-III (Supplementary Fig. 5B). In this case, HES1/5 proteins act as a transcription repressor of *MASH1* gene, which is the activator of the production of NGN1 and β-TUBULIN-III proteins. It is also observed that during the periodic intervals, at which the HES1/HES5 protein expressions are at the lower level, the expressions of neuronal marker proteins are found at the higher level and differentiation of neuron progenitor cells are occurred (i.e. neurogenesis). On the other hand, it is observed that when the expression dynamics of HES1/5 protein are at the up-regulated state, and JAK2/STAT3 proteins are also simultaneously expressed, then the expression of the marker protein GFAP, responsible for astrocytes differentiation (ASPC) is at ON state. In this case, the entire reaction motif of Notch signaling network is redirected towards gliogenesis process (Supplementary Fig. 5C).

These entire feedback reaction mechanisms governing neurogenesis and gliogenesis processes can sometimes go awry, if the tumor suppressor protein P53 is absent in the neural stem cells. In that case, anti-apoptotic proteins such as BCL2, IAP, and FLIP will be expressed at higher quantities and the stem cells become immortal. However, due to the active regulations of both the positive and negative feedback loops, Notch signaling network will produce HES1/5, CYCLINS at regular interval of time (Supplementary Fig. 5D). This will eventually lead the rapid proliferations of the stem cells without any apoptosis and the cells will be converted into Glioblastoma stem cells (GSCs).

### *3.3 State transition graph of General Glioma model simulation*

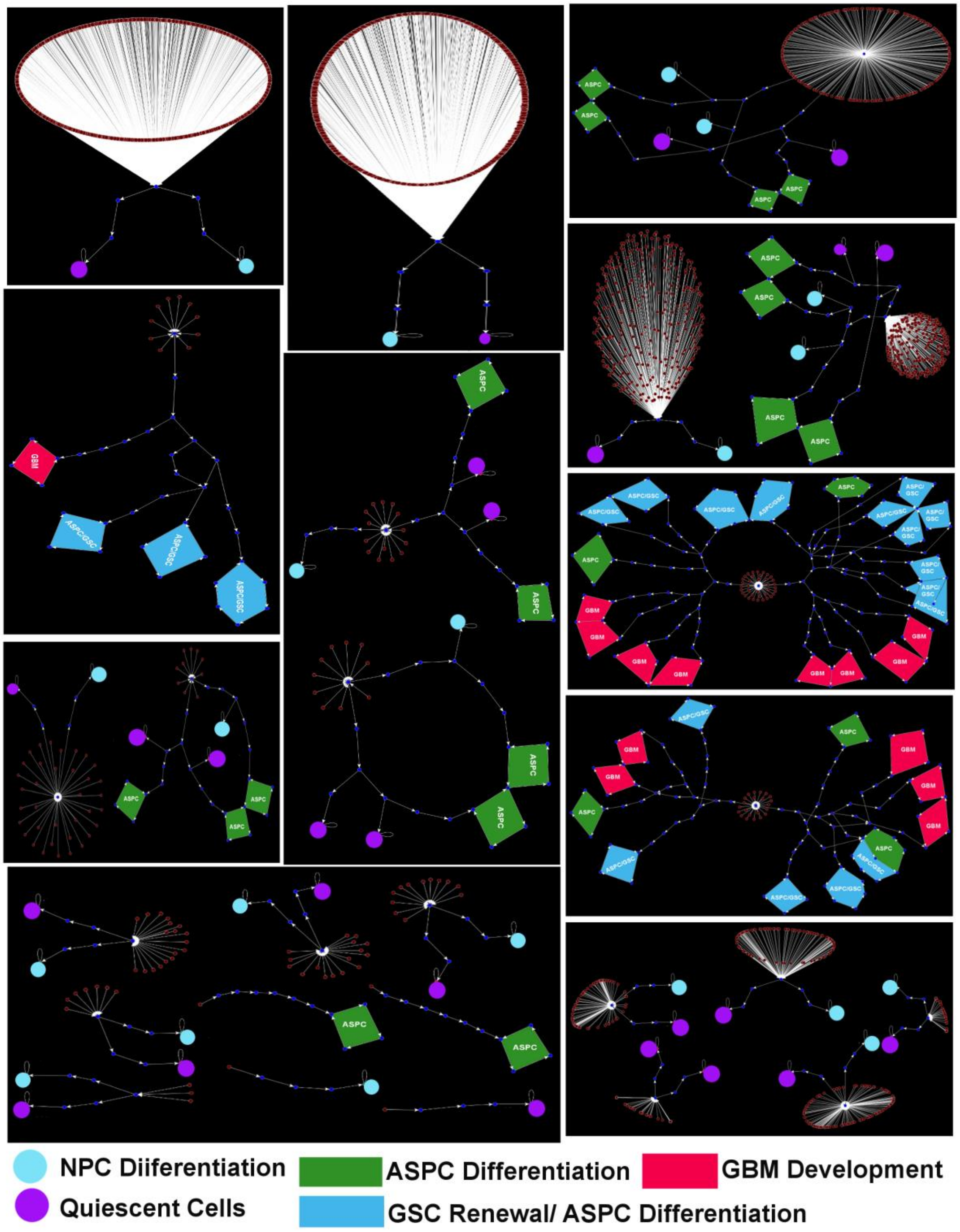


**Supplementary Fig. 6. State transition graph (STG) generated from general glioma model simulation data.** NPC Differentiation and Quiescent cells are the fixed-point attractors and the others represent the cyclic attractor states corresponding to different sub-types of GBM tumor cells.

### *3.4 Protein activity pattern observed in different attractor states corresponding to Grade-IV cells*

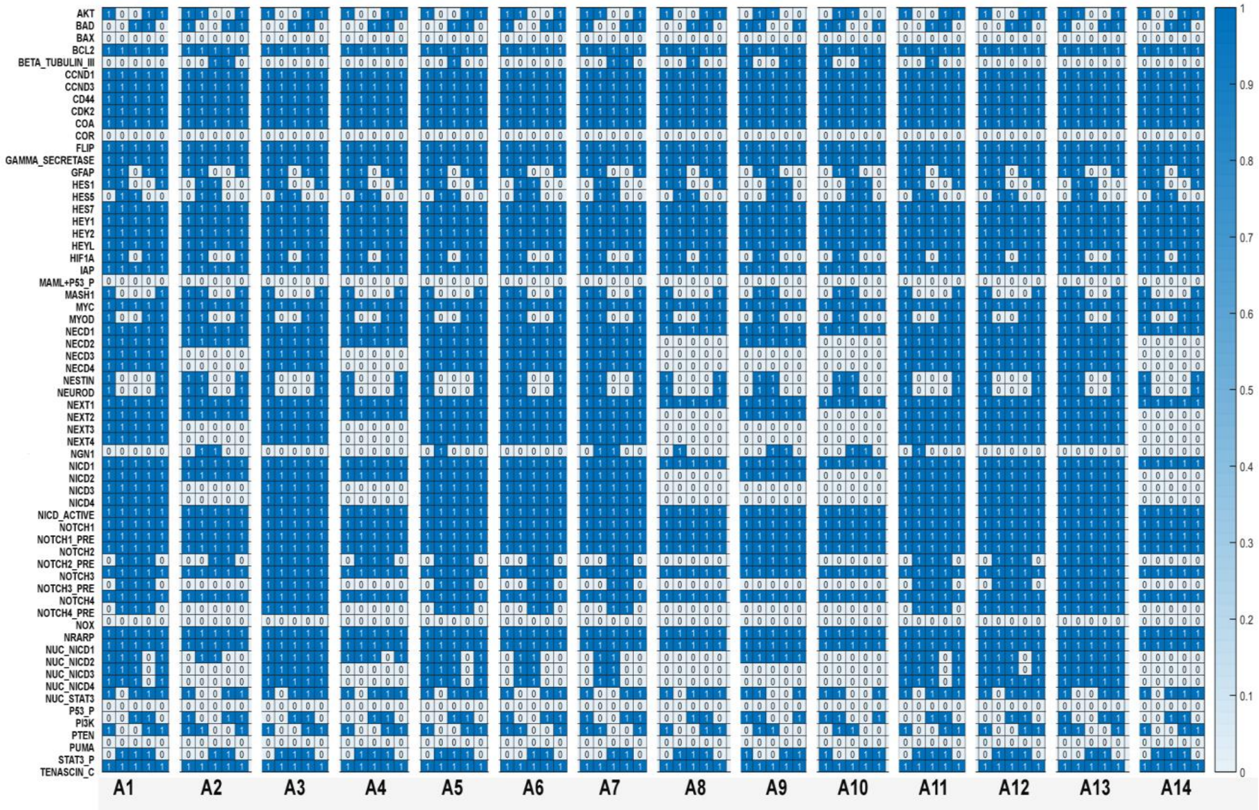


**Supplementary Fig. 7. Activity patterns of the pathway molecules observed in all the periodic attractor states corresponding to Grade-IV tumor cells in the general glioma model simulation.** The temporal expression/activity profiles (ON/Up-regulation OR OFF/Down-regulation states) of the pathway molecules in each of the distinct attractor states (total 14) clearly depict the intra-tumor heterogeneities, which exist within the heterogeneous groups of Grade-IV tumor cells found in GBM tumor ecosystem. Such heterogeneities arise within the heterogeneous population of tumor cells within a same grade due to the proteomic/genomic variations present in the Notch pathway components and its interactions with the microenvironment of the tumor niche. For example, the temporal dynamics of the activities of important proteins (e.g. HES1, HES5, HIF1A, MASH1, NESTIN, NEUROD, NGN1, Precursors of NOTC2/3/4, PI3K, PTEN, Phosphorylated form of STAT3 etc.) are found significantly varying (not synchronized or absent) within these periodic attractor states. The time-points of the activations of these proteins within the attractor states greatly influence other downstream proteins/molecules and finally the temporal dynamics of the expression of Grade-IV tumor cells.

### *3.5 Distributions of the phenotype predictor scores observed for all type of cellular states*

The shapes of the distributions of the phenotype predictor scores observed for all the distinct cellular states found in aNSC, GSC, General Glioma, and Grade-IV GBM models have fat tailed, Cauchy-like distribution (Supplementary Fig. 8). This type of distribution is observed for the ratio distribution of two multivariate normally distributed variables. Here, by definition (***Eq. 10***), the

phenotype predictor score is the ratio of the distributions of the total number of occurrences of the cellular state to the associated total cost.

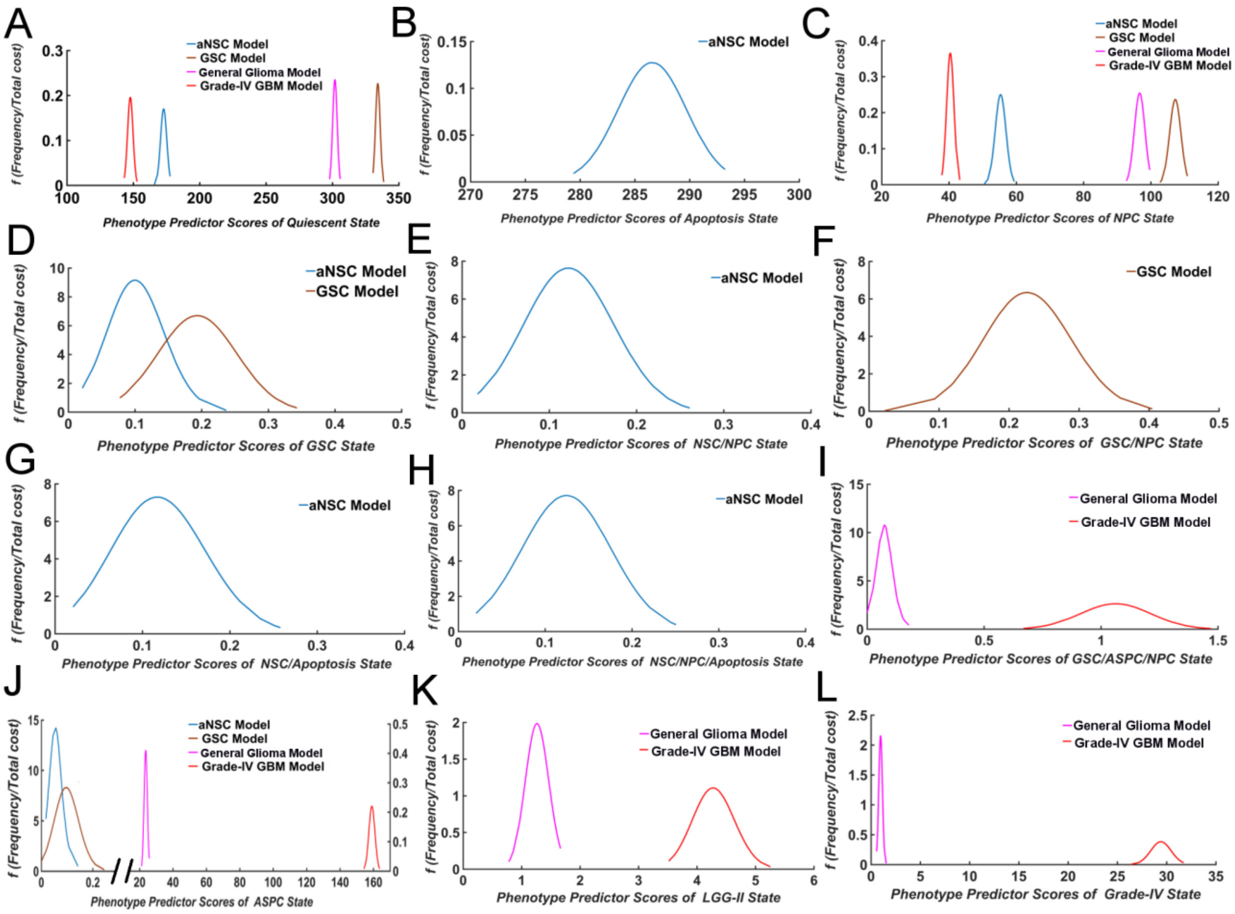


**Supplementary Fig. 8. Distributions of the Phenotype Predictor Scores observed for different cellular states in aNSC, GSC, General Glioma and Grade-IV GBM models.** Distributions are plotted for all distinctly observed cellular states (total 12) *viz.* (A) Quiescent state; (B) Apoptosis; (C) NPC Differentiation; (D) GSC Renewal; (E) NSC Renewal/NPC Differentiation; (F) GSC Renewal/NPC Differentiation; (G) NSC Renewal/Apoptosis; (H) NSC Renewal/ NPC Differentiation/Apoptosis; (I) GSC Renewal/ASPC Differentiation/ NPC Differentiation; (J) ASPC Differentiation (LGG-I); (K) GSC Renewal/ASPC Differentiation (LGG-II); (L) GSC Renewal/ASPC Differentiation/GBM Development (Grade-IV) state.

A detailed description about the properties and the expected shape of such distribution is deduced and elaborately discussed in the literatures [25,54]. Although the theoretical mean or the expected value and the variance are zero for such distribution, but numerically the mean values of the phenotype predictor scores can be calculated from the original data by calculating the ratio of the mean normalized frequency to mean value of the total costs of the cellular states (Supplementary Table 4). The confidence interval (CI) of the mean value of the ratio of two variables (frequency and cost) can be calculated by using Fieller's theorem at desired confidence level [55,56].

**Supplementary Table 4. Mean values with 95% CI of the phenotype predictor scores of all cellular states.**

| **Observed Cellular States or Phenotypes** | **Models** | | | |
|---|---|---|---|---|
| | **aNSC**<br>Mean<br>[95 % CI] | **GSC**<br>Mean<br>[95 % CI] | **General Glioma**<br>Mean<br>[95 % CI] | **Grade-IV GBM**<br>Mean<br>[95 % CI] |
| Quiescent | 172.813<br>[171.596 174.032] | 334.160<br>[333.246 335.074] | 301.827<br>[300.945 302.709] | 147.688<br>[146.630 148.747] |
| Apoptosis | 286.581<br>[284.957 288.206] | NA | NA | NA |
| NPC Differentiation | 55.326<br>[54.496 56.156] | 107.264<br>[106.388 108.140] | 96.677<br>[95.862 97.492] | 40.372<br>[39.804 40.940] |
| GSC Renewal | 0.100<br>[0.078 0.124] | 0.193<br>[0.163 0.224] | NA | NA |
| NSC Renewal/NPC Differentiation | 0.123<br>[0.095 0.150] | NA | NA | NA |
| GSC Renewal/NPC Differentiation | NA | 0.226<br>[0.194 0.259] | NA | NA |
| NSC Renewal/Apoptosis | 0.118<br>[0.090 0.147] | NA | NA | NA |
| NSC Renewal/NPC Differentiation/Apoptosis | 0.124<br>[0.098 0.151] | NA | NA | NA |
| GSC Renewal/ASPC Differentiation/ NPC Differentiation | NA | NA | 0.072<br>[0.053 0.0917] | 1.063<br>[0.984 1.141] |
| ASPC Differentiation (LGG-I) | 0.054<br>[0.040 0.069] | 0.098<br>[0.073 0.123] | 23.655<br>[23.150 24.160] | 159.059<br>[158.118 160.000] |
| GSC Renewal/ASPC Differentiation (LGG-II) | NA | NA | 1.265<br>[1.161 1.370] | 4.282<br>[4.096 4.470] |
| GSC Renewal/ASPC Differentiation/GBM Development (Grade-IV) | NA | NA | 0.970<br>[0.873 1.066] | 29.414<br>[28.875 29.955] |

# 4 Case Studies

## *4.1 Case study using TCGA-LGG & TCGA-GBM RNASeq samples data*

### 4.1.1.Selection of patient cohorts and preparation of RNASeq sample data sets

Two patient cohorts consist of low-grade (TCGA-LGG) and high-grade Glioblastoma (TCGA-GBM) from "The Cancer Genome Atlas (TCGA)" research networks [57,58] are chosen for

the case studies in this present work. HTSeq raw counts files (RNASeq experiment) of the Glioblastoma patients with TP53 mutation are selected to determine the mRNA expression profiles of these two patient cohorts. The following is the statistics of these two cohorts from which the RNASeq raw count data are downloaded on 27th April, 2017 (Supplementary Table 5).

**Supplementary Table 5. Statistics of the TCGA Glioblastoma patient cohorts.**

| **Cohorts** | **# of Cases (Patients)** | | | | **# of Samples (RNA-Seq Raw Counts Data)** | | | |
|---|---|---|---|---|---|---|---|---|
| | M | F | U | Total | # Normal Tumor Samples | # Primary Tumor Samples | # Recurrent Tumor Samples | Total |
| TCGA-LGG (General) | 282 | 228 | 1 | 511 | 0 | 513 | 16 | 529 |
| TCGA-LGG (TP53 Mutated) | 141 | 100 | 1 | 242 | 0 | 240 | 15 | 255 |
| TCGA-GBM (General) | 366 | 230 | 21 | 617 | 5 | 13 | 156 | 174 |
| TCGA-GBM (TP53 Mutated) | 32 | 21 | 0 | 53 | 0 | 57 | 0 | 57 |

**M = Male, F = Female, U = Undefined/Not mentioned**

The TCGA-Low grade glioma (TCGA-LGG) cohort contains tumor samples from Grade-II and Grade-III glioblastoma patients, whereas the TCGA-GBM cohort contains samples from Grade-IV tumor patients. The RNASeq raw counts data (available as TXT files) for each patient is available in the open source GDC Data portal of National Cancer Institute (https://portal.gdc.cancer.gov/). Further information about the workflows related to sample collection; mRNA sequencing and data processing, read alignments, and mRNA quantification etc. are available in GDC Data User's Guide (https://docs.gdc.cancer.gov/Data/PDF/Data_UG.pdf).

### 4.1.2.Differential expression analyses of mRNA molecules

The RNASeq raw counts data files corresponding to the patient cohort, which have TP53 mutation, are extracted from the TCGA-LGG (General) and TCGA-GBM (General) patient cohorts.

Differential expression analyses are performed by forming the contrasts between primary (TP53 mutated) (i) LGG (total samples = 240) and (ii) GBM (total samples = 57) tumor samples versus solid normal tumor (total samples = 5) samples using "edgeR" statistical package [59]. The mRNA molecules of the following proteins from the set of input proteins (mentioned in Supplementary Table 1) are found to be significantly expressing (up or down) in the differential expression analyses (Supplementary Table 6). The mRNA expression patterns observed in this analysis are considered as the transcriptomics profiles of the two different patient cohorts.

**Supplementary Table 6: Differentiation expression of the mRNA molecules in two different patient cohorts.**

| **TCGA-GBM (TP53 Mutation) Vs. Normal solid tumor** | | **TCGA-LGG (TP53 Mutation) Vs. Normal solid tumor** | |
|---|---|---|---|
| Up Regulation | Down Regulation | Up Regulation | Down Regulation |
| APH1,DLL3, FRINGE,GASE, HAT,HDAC,JAG1, MAGP1,MAGP2, NEDD4,POFUT1, SAP30 | CNTN1,DVL,DTX1, FBW7, JAG2, JIP1 | DLL1, DLL3, FRINGE, NEDD4, POFUT1 | FBW7, JAG2, NOV |

### *4.2 Logical simulations using TCGA-LGG and TCGA-GBM transcriptomics data:*

The transcriptomics profiles of the input protein molecules observed in both the TCGA-LGG and TCGA-GBM patient cohorts are taken as inputs for the further simulations of aNSC and general glioma model simulations.

#### 4.2.1 Analyses of TCGA-LGG patient cohort

In the TCGA-LGG patient cohort there are total 5 and 3 protein molecules found to be over and under expressed, respectively (Supplementary Table 4). Mutation or down regulation of P53 protein is also considered. Hence, out of total 53 input molecules of the master aNSC model, there are total 9 protein molecules kept frozen (i.e. constitutively expressed at ON or OFF state) and eliminated from the input list for further randomization in the new simulation. Hence, the total mutations $\mu$ introduced in the newly developed, TCGA-LGG transcriptomics data on master aNSC model is 9 and the rest 44 input proteins are randomized 10000 times in 10 separate batches. The mean normalized frequency values of each cellular state are calculated from these 10 independent simulation batches, which will be further used for checking the goodness-of-fit with the normalized frequency values observed for the cellular states in the master aNSC model. The objective of this study is to assess the effects of these 9 differentially expressed transcripts of the input proteins in the development of adult NSCs within the neurogenic niche of human brain and how much the normalized frequency distributions of different cellular states are varied with respect to the distributions of the cellular states observed in the master aNSC model (Fig. 1B). Chi-square goodness of fit test is used to compare the normalized frequency distributions of this new model (i.e. observed normalized frequencies) with the distributions (i.e. expected normalized frequencies) observed in the master aNSC model. The statistics is performed under the following null ($H_0$) and alternate ($H_1$) hypotheses:

*$H_0$ = The normalized frequency values observed in the two model simulations (i.e. expected and observed) are consistent with each other.*

*$H_1$ = The normalized frequency values observed in the two model simulations (i.e. expected and observed) are **not** consistent with each other.*

The comparative statistics of the normalized frequency distributions of each cellular state observed in these two simulations is represented in Fig. 5A, and elaborately discussed in the main manuscript.

Following this simulation, the TCGA-LGG transcriptomics data is considered as inputs in the previously developed, master model of general glioma development. In the general glioma model, there are already 4 mutations added (including P53) and 8 mutations are further added as per the TCGA-LGG transcriptomics expression profile (Supplementary Table 4). Hence, altogether there are total 12 mutations ($\mu$) introduced in the master general glioma model and a new derivative model is developed by keeping these 12 input proteins at constitutively up or down regulated states. These 12 input proteins are kept aside for further randomization and the rest 41 input proteins are randomized 10000 times in 10 separate batches. The mean normalized frequency values of each cellular state (observed normalized frequencies) are calculated from these 10 simulation batches and Chi-square goodness-of-fit test considering the null ($H_0$) and alternate ($H_1$) hypotheses is performed again to assess the effects of the newly introduced mutations on the normalized frequency values of each cellular state (i.e. expected normalized frequencies) found in the master, general glioma model simulation. The comparative statistics of the normalized frequency distributions of each cellular state observed in these two simulations is represented in Fig. 5B, and elaborately discussed in the main manuscript.

### 4.2.2 Analyses of TCGA-GBM patient cohort

Similar to the analyses of TCGA-LGG tumor samples cohort, the transcriptomics profile (Supplementary Table 4) observed for the TCGA-GBM sample cohort is also considered at first as inputs in the master aNSC and general glioma models. Total 12 and 6 proteins are found to be up and down regulated, respectively, in the differential expression analyses in the tumor sample cohort with identified TP53 mutation. Therefore, in total 19 mutations ($\mu = 12 + 6 +1$) of the input proteins are considered in the input in the master aNSC model and a new model using the TCGA-GBM transcriptomics data on master aNSC model is developed. Here, the extra 1 mutation is added for the P53 mutation. Similar to the previous analyses, these 19 input proteins are kept constitutively at ON or OFF state as per the differentiation expression results (Supplementary Table 4) and the rest 34 input proteins out of 53 are further randomized 10000 times in 10 separate simulation batches. Similar Chi-square goodness-of-fit test is performed here to assess the similarities of the normalized frequency values of each cellular state observed in master aNSC and new model simulations. Simulation results and the comparative statistics of the normalized frequency distributions between these two models are depicted in Fig. 5C as well as elaborately discussed in the main manuscript.

On the other hand, another set of simulation is performed to examine the effects of the differentially expressed transcripts of the TCGA-GBM tumor samples cohort (Supplementary Table 4) on the master general glioma model. Here, the master general glioma model contains 4 mutations (TP53, JAK2, STAT3, and RBPJ) and in the TCGA-GBM cohort another 18 proteins are found to be differentially expressed. Therefore, in total there are 22 mutations ($\mu = 18 + 4$) will be added in the new simulation. These 22 proteins are kept constitutively expressed (either up or down) based on the transcriptomics profile generated from differential expression analyses (Supplementary Table 4) and the rest 31 out of 53 input proteins are randomized 10000 times in 10 separate batches. Similar Chi-square goodness-of-fit test is also performed to study the

similarities of the normalized frequency values of each cellular state observed in master general glioma model and new model simulations. A comparative statistics of the cellular states observed in this new model as compare to the master general glioma model is represented in Fig. 5D and also discussed in the main manuscript.

# 5 Methodologies used for drug target screening

The simulation outcomes of "Grade-IV GBM model" simulation show significant increase in the number of Grade-IV tumor cells (i.e. the cellular state: "GSC Renewal/ASPC Differentiation/GBM Development") in the attractor space (Fig. 2C). The periodic state transition dynamic in the steady state level observed in this cellular state is mainly occurred due to the presence of cyclic expression patterns of its corresponding marker proteins (Supplementary Table 3). The expressions of these marker proteins are dependent on the intermediated molecules through which the flow of signaling cascades are transduced from the input to the target molecules during tumorigenesis. Hence the molecules, which show higher correlations in their expression pattern with the marker proteins, are the key regulators of the flow of tumorigenic signal inside the developing Grade-IV tumor cells. Therefore, it can be hypothesized that a subset of intracellular intermediate molecules, strongly tuned and correlated with the activity pattern of Grade-IV cellular state via the marker proteins are the most significant molecules for the sustainment of this cellular state. Indeed, it is expected that perturbing the expressions (i.e. logical states) of such molecules (individually or in combination) will alter the activity pattern of the Grade-IV cellular state and those proteins will be considered as potential drug targets. However, identification such small subset of molecules out of the large set of modeled pathway molecules is a challenging task, which could be only possible to resolve through an efficient computational algorithm capable of analyzing multiple time series data simultaneously.

The intermediate molecules which are mutually interconnected and highly correlated with the temporal activity pattern observed for Grade-IV cellular state in the "Grade-IV GBM" model simulation study are required to be extracted [60]. The correlation and delay between a pair of time course data could be calculated by using Fast Fourier Transform (FFT) analysis. Hence, the delay and pair-wise correlation between the activity patterns of Grade-IV tumorigenic cellular state (i.e. Target signal) and the time-course logical expressions data of all the intermediated molecules (i.e. Query signal) are measured by the following method.

Let us consider that $C^{Grade-IV} = \{c_1, c_2, c_3, ....., c_T\}$ is the time series activity (ON or 1 and OFF or 0) profile of the Grade-IV cellular state observed in the STG of "Grade-IV GBM model" simulation at the discrete time points $t = 1, 2, 3, ....., T$ . This time-course data of Grade-IV cellular state is considered as the "Target" signal. Similarly, let us consider that the time-course logical expression (ON/1, OFF/0) profile of any arbitrary molecule $X_i = \{x_1, x_2, x_3, ....., x_T\}$ , which is considered as "Query" signal. Both the temporal signals (S) are decomposed into cyclic patterns (i.e. frequency domain) with each frequency $n = 1, 2, 3, ......, T-1$ by following FFT analyses as shown in ***Eq. 13 & Eq. 14*** [60].

$$C_n = \frac{1}{T}\sum_{n=1}^{T-1} c_t e^{-i2\pi n\frac{t}{T}} \quad (13)$$

$$X_n = \frac{1}{T}\sum_{n=1}^{T-1} x_t e^{-i2\pi n\frac{t}{T}} \quad (14)$$

Amplitude of the cycle with frequency $n = 0$ is neglected. The amplitudes and phase angles of the cycles with higher frequencies (n > 0) are calculated for both the signals. The frequency of the cycle (*n*) for which the amplitude is found at maximum magnitude is at first identified. After that phase angles of the cycles from both the target and query signals $(\phi_C^n, \phi_X^n)$ are calculated at that frequency (*n*) and the difference or delay $\Delta^n{}_{CX} = \phi_C^n - \phi_X^n$ between the two signals is measured. The delay between two signals $\delta_{CX}$ is further calculated in the range of $0$ *to* $\frac{T}{n}$ by using the following ***Eq. 15*** [60].

$$\delta_{CX} = \frac{\Delta_{CX}^n}{\left(\frac{360n}{T}\right)} \quad (15)$$

Lagged Pearson correlation coefficient is also calculated for measuring the strength and association between the two signals or trajectories [60]. In this work the delay and correlation between Grade-IV cellular state and for each pathway molecules are measured pair-wise. There are total six probable outcomes, which are found while comparing all such pairs of trajectories (i.e. Target vs. Query) using this approach. These entire mathematical calculations are done in "DynOmics" package developed in the statistical package "R" [60].

***i)*** The signals are positively correlated (correlation > 0) with no delay (Delay = 0). In this case both target and query signals are superimposed on each other and the phase (or direction) of the signals is same (i.e. positively correlated). Hence, it is considered that the molecular expression pattern (i.e. query signal) is positively influencing the activity pattern or dynamics of Grade-IV tumor state and there is no delay between the time-course profiles. Therefore, the molecule is a strong positive inducer or activator of Grade-IV tumor cells, which means when the molecule is at ON (i.e. up -regulated/ active) state, the activity of Grade-IV cellular state is also High (or ON).

***ii)*** The signals are positively correlated (correlation > 0) with negative delay (Delay < 0). In this case the phase (or direction) of both the signals is same (i.e. positively correlated), but the initiation of the target signal at initial time point is delayed with respect to the query signal. Hence, it is considered that the molecular expression pattern (i.e. query signal) is positively influencing the activity pattern or dynamics of Grade-IV tumor state, but there is a lag of the Grade-IV time-course activity profile with the respect to that positively influencing molecule. Therefore, the molecule is a positive inducer or activator of Grade-IV tumor cells, which means activation of these molecules will lead to the higher expression of Grade-IV tumor state.

***iii)*** The signals are negatively correlated (correlation < 0) with positive delay (Delay > 0). In this case the phases of the signals are opposite (i.e. negatively correlated) and the initiation of the target signal is ahead of the query signal at initial time point. Hence, it is considered that the

molecular expression pattern (i.e. query signal) is negatively influencing the activity pattern or dynamics of Grade-IV tumor state, but the Grade-IV time-course activity profile is running ahead with the respect to that negatively influencing molecule. Therefore, the molecule is a negative inducer or activator of Grade-IV tumor cells, which means which means when the molecule is at ON (i.e. up -regulated/ active) state, the activity of Grade-IV cellular state is Low (or OFF).

**Supplementary Table 7: Delay difference and significant correlation observed between Grade-IV trajectory and pathway molecules.**

| Original Signal | Query Signal | Delay $\leq 3$ | Correlation >= 0.6 | P-Value |
|---|---|---|---|---|
| Grade-IV | GFAP | 1 | 1 | 0 |
| Grade-IV | HIF1A* | 1 | 1 | 0 |
| Grade-IV | NUC_STAT3* | 2 | 1 | 0 |
| Grade-IV | MASH1* | 3 | -1 | 0 |
| Grade-IV | NESTIN | 3 | -1 | 0 |
| Grade-IV | NEUROD | 3 | -1 | 0 |
| Grade-IV | PTEN* | 3 | -1 | 0 |
| Grade-IV | STAT3_P* | 3 | 1 | 0 |
| Grade-IV | BAD | 0 | -0.7698004 | 0.005588 |
| Grade-IV | AKT* | 1 | 0.7637626 | 0.010131 |
| Grade-IV | NGN1* | 2 | -0.7559289 | 0.018452 |
| Grade-IV | PI3K* | 2 | 0.7559289 | 0.018452 |
| Grade-IV | HES1 | 0 | 0.6236096 | 0.040347 |
| Grade-IV | HES5 | 0 | 0.6236096 | 0.040347 |

***Proteins selected for drug target screening as they do not belong to the class of marker proteins responsible for defining different cellular states.**

***iv)*** The signals are negatively correlated (correlation < 0) with no delay (Delay = 0). In this case the phases of the signals are opposite (i.e. negatively correlated), but there is no delay at the initiation of the signals at initial time point. Hence, it is considered that the molecular expression pattern (i.e. query signal) is negatively influencing the activity pattern or dynamics of Grade-IV tumor state, but there is no lag between the trajectories of these two signals. Therefore, the molecule is a strong negative inducer or inhibitor of Grade-IV tumor cells which means activations of these molecules, will inhibit the expression or activation of Grade-IV tumorigenic state.

***v)*** The signals are negatively correlated (correlation < 0) with negatively delay (Delay < 0). In this case the phases of the signals are opposite (i.e. negatively correlated), but the initiation of the target signal at initial time point is delayed with respect to the query signal. Hence, it is considered that the molecular expression pattern (i.e. query signal) is negatively influencing the activity pattern or dynamics of Grade-IV tumor state, but there is a lag of the Grade-IV time-course activity profile with the respect to that negatively influencing molecule. Therefore, the

molecule is a negative inducer or inhibitor of Grade-IV tumor cells which means activations of these molecules, will inhibit the expression or activation of Grade-IV tumorigenic state.

***vi)*** The signals are positively correlated (correlation > 0) with positive delay (Delay > 0). In this case the phase (or direction) of both the signals is same (i.e. positively correlated), and the initiation of the target signal is ahead of the query signal at initial time point. Hence, it is considered that the molecular expression pattern (i.e. query signal) is positively influencing the activity pattern or dynamics of Grade-IV tumor state, but the Grade-IV time-course activity profile is running ahead with respect to that negatively influencing molecule. Therefore, the molecule is a positive inducer or activator of Grade-IV tumor cells which means activations of these molecules, will lead to the higher expression of Grade-IV tumor state.

The pathway molecules, which show significant positive correlation (correlation value ≥ 0.6) and Delay ≤ 3 with the activity trajectory of Grade-IV cellular state, are enlisted in Supplementary Table 7.

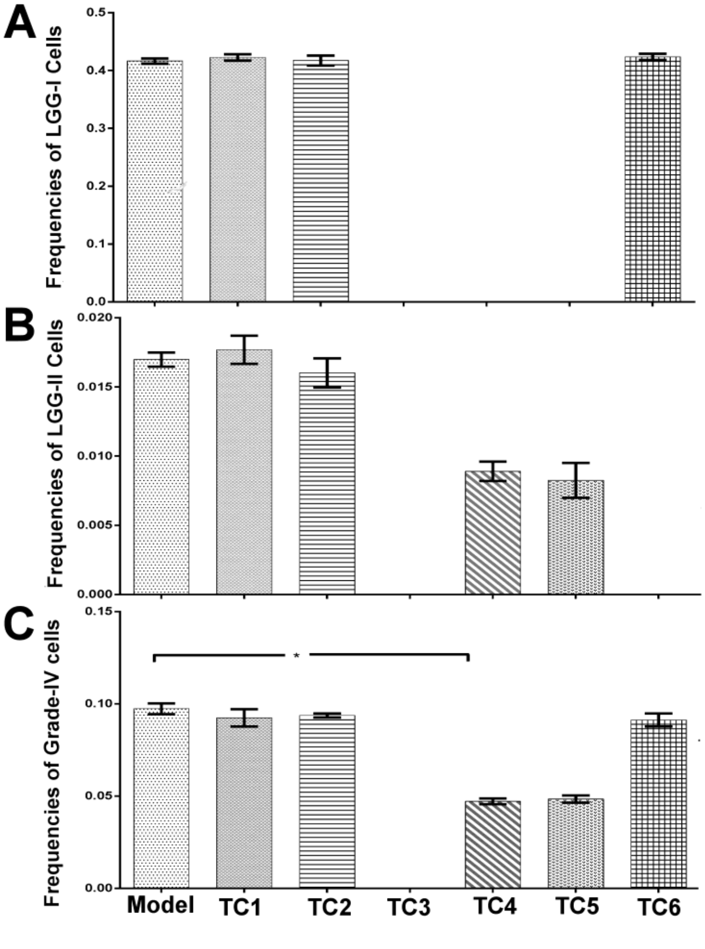


**Supplementary Fig. 9. Simulation outcomes of drug targets screening analyses.** Normalized frequencies observed for (A) LGG-I (B) LGG-II and (C) Grade-IV tumor cells in High-grade GBM model and different target screening scenarios are shown here. TC1: HIF1A Inhibition; TC2: PI3K & AKT Inhibition; TC3: STAT3 Inhibition; TC4: MASH1 & NGN1 Activation; TC5: PI3K Inhibition & MASH1 & NGN1 Activation; TC6: PI3K & AKT & HIF1A Inhibition. (* P-Value ≤0.05).

**Perturbation study:** The molecules enlisted in Supplementary Table 7 showing positive correlation with the activity profile of Grade-IV cellular state are suppressed (i.e. targeted) by freezing the expression states as down-regulated (or OFF) state in the "Grade-IV GBM" model. On the other hand, the molecules, which are negatively correlated, are kept up-regulated (i.e. ON) in the perturbation study. The outcomes of targeting these identified proteins (individually or combinations) in terms of normalized frequency distributions are shown in Supplementary Fig. 9. In Supplementary Table 8, the molecules which have absolute correlation value ≥ 0.75 with P-value < 0.05, Delay ≤ 3 and shown profound effect of reducing the activity of LGG-I, LGG-II, and Grade-IV cellular states while perturbing their expressions in the "Grade-IV GBM" model are shown rank wise. Here, the activity profiles of LGG-I and LGG-II cellular states are also assessed to analyze the suppressing effects of targeting the selected protein on all sub-types of GBM tumor.

**Supplementary Table 8. Calculated ranks of the effective drug targets for suppressing Grade-IV tumor cells.**

| Target Cellular State | Query Proteins | Delay ≤3 | \|Correlation\| ≥ 0.75 | P-Value | Rank |
|---|---|---|---|---|---|
| GSC/ASPC/GBM | NUC_STAT3* | 2 | 1 | ~ 0 | 1st |
| GSC/ASPC/GBM | NGN1* | 2 | -0.7559289 | 0.018452 | 2nd |
| GSC/ASPC/GBM | STAT3_P* | 3 | 1 | ~ 0 | 1st |
| GSC/ASPC/GBM | MASH1* | 3 | -1 | ~ 0 | 2nd |

There are four groups of drug targets, which are mentioned in Supplementary Table 7 *viz*. i) PI3K/AKT, ii) STAT3/Nuc_STAT3, iii) MASH1, and iv) HIF1A, are considered for further drug target screening and ranking simulation analyses. The simulation results show that the perturbations of STAT3/Nuc_STAT3 or MASH1 proteins individually are highly effective than perturbing the other molecules from the remaining two groups *viz*. (i) PI3K/AKT and (iv) HIF1A. It can be also concluded that the protein molecules showing maximum delay ≤ 3 and correlation ≥ 0.6 with the target signal (i.e. expression dynamics of Grade-IV) can be allowed to screen the suitable proteins as drug targets (Supplementary Table 7).

### *5.1 Flow-chart of the decision making protocols used in cancer risk prediction, tumor grade detection and drug target screening*

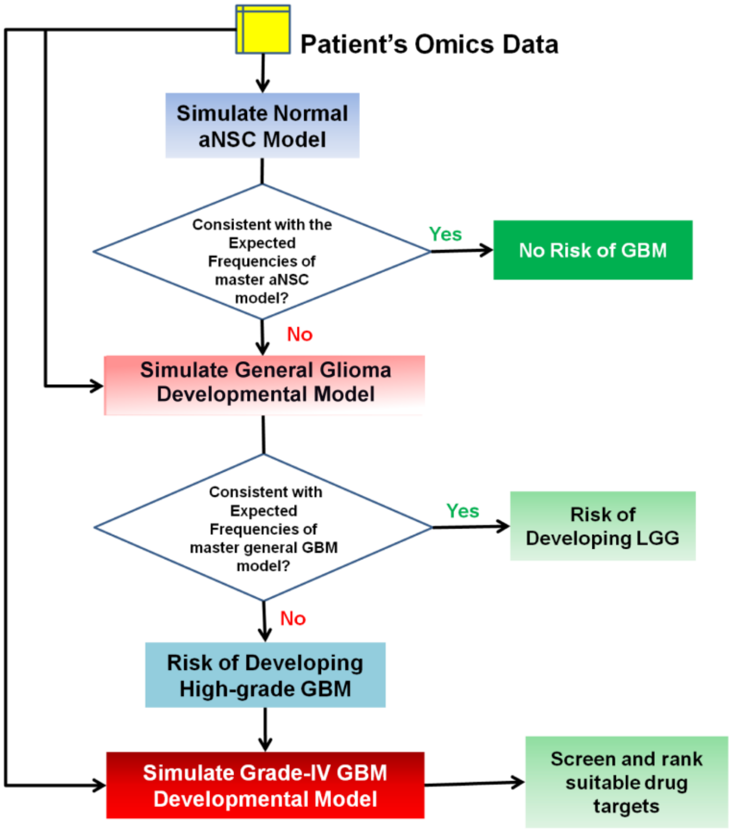


**Supplementary Fig. 10. Stepwise proposed protocol for the decision making steps used in the personalized GBM therapeutics studies.**

This proposed flow-chart describes the step-wise protocols which can be followed to predict the risk of future occurrence of GBM tumor of an individual (Supplementary Fig. 10). Two models *viz.* master aNSC and general glioma model would be used for this purpose, in which the omics (e.g. transcriptomics, proteomics, epigenomics etc.) profile of the individual will be used as inputs. At first, the transcriptomics profile will be taken as inputs in the master aNSC model to run a new simulation. The normalized frequencies of each of the cellular state (i.e. observed values) generated from the new simulation will be compared (Chi-square goodness-of-fit test) with the normalized frequencies of the cellular states (i.e. expected values) observed in the master aNSC model.. If the observed normalized frequencies are found consistent with respect to the expected normalized frequencies at the desired significance level, then the inspected individual will be categorized as healthy who have no risk of developing GBM tumor in the near future. On the other hand, if there is an inconsistency found in the observed and expected normalized frequencies, then the individual's transcriptomics profile would be further tested to detect the risk of developing Low or High grade GBM tumor in the future. In this case, the transcriptomics profile will be considered as inputs in the master general glioma model and a new simulation will be run again. If the observed and expected normalized frequencies are well fitted with each other,

then it can be concluded that the individual has a risk of developing Low-grade glioblastoma (LGG) in future. To quantify the chances of occurrences of the tumor cells of that individual, the phenotype predictor scores will be calculated for each tumorigenic state (i.e. LGG-I, LGG-II and Grade-IV), which will be further helpful to assess the severity of the tumor progression. Otherwise, if it is observed that the expected and observed normalized frequencies are not consistent with each other and the phenotype predictor score of all the tumorigenic states (especially Grade-IV) are increased many folds as compared to the master general glioma model, then it can be concluded that the individual has the risk of developing high grade GBM tumor. Quantification and further assessments of the chances of developing high-grade GBM tumor can be calculated by measuring the corresponding values of the phenotype predictor scores. Followed by these analyses, for high-grade GBM tumor patients, another simulation will be performed by using the omics profile as input in the master Grade-IV GBM model to screen and rank suitable drug targets for personalized, target based GBM tumor therapy. Hence, it can be seen that this entire novel computational pipe-line can be easily deployed for the personalized, glioblastoma related pathological studies (such as risk prediction, tumor grade detection, biomarker identification and ranking of most potential drug targets etc.) by using the omics data of GBM (or suspected) patients.

# 6 References:


1 Chowdhury, S. & Sarkar, R. Drug Targets and Biomarker Identification from Computational Study of Human Notch Signaling Pathway. *Clin Exp Pharmacol* **3**, 2161-1459.1000137 (2013).

2 Chowdhury, S., Pradhan, R. N. & Sarkar, R. R. Structural and logical analysis of a comprehensive hedgehog signaling pathway to identify alternative drug targets for glioma, colon and pancreatic cancer. *PloS one* **8**, e69132, doi:10.1371/journal.pone.0069132 (2013).

3 Zhang, R. *et al.* Network model of survival signaling in large granular lymphocyte leukemia. *Proc Natl Acad Sci U S A* **105**, 16308-16313, doi:10.1073/pnas.0806447105 (2008).

4 Klamt, S., Saez-Rodriguez, J. & Gilles, E. D. Structural and functional analysis of cellular networks with CellNetAnalyzer. *BMC systems biology* **1**, 2 (2007).

5 Raeymaekers, L. Dynamics of Boolean networks controlled by biologically meaningful functions. *J. Theor. Biol.* **218**, 331-341 (2002).

6 Shih, A. H. & Holland, E. C. Notch signaling enhances nestin expression in gliomas. *Neoplasia* **8**, 1072-1082, doi:10.1593/neo.06526 (2006).

7 Imayoshi, I. & Kageyama, R. The role of Notch signaling in adult neurogenesis. *Mol. Neurobiol.* **44**, 7-12, doi:10.1007/s12035-011-8186-0 (2011).

8 Anilkumar, U. & Prehn, J. H. Anti-apoptotic BCL-2 family proteins in acute neural injury. *Front Cell Neurosci* **8**, 281, doi:10.3389/fncel.2014.00281 (2014).

9 Liu, G. *et al.* Analysis of gene expression and chemoresistance of CD133+ cancer stem cells in glioblastoma. *Mol Cancer* **5**, 67, doi:10.1186/1476-4598-5-67 (2006).

10 Cenciarelli, C. *et al.* The interference of Notch1 target Hes1 affects cell growth, differentiation and invasiveness of glioblastoma stem cells through modulation of multiple oncogenic targets. *Oncotarget* **8**, 17873-17886, doi:10.18632/oncotarget.15013 (2017).

11 Balenci, L. & van der Kooy, D. Notch signaling induces retinal stem-like properties in perinatal neural retina progenitors and promotes symmetric divisions in adult retinal stem cells. *Stem Cells Dev* **23**, 230-244, doi:10.1089/scd.2013.0177 (2014).

12 Gomez-Nicola, D., Valle-Argos, B., Pallas-Bazarra, N. & Nieto-Sampedro, M. Interleukin-15 regulates proliferation and self-renewal of adult neural stem cells. *Mol Biol Cell* **22**, 1960-1970, doi:10.1091/mbc.E11-01-0053 (2011).

13 Alexander, J. E. *et al.* Characterization of posttranslational modifications in neuron-specific class III beta-tubulin by mass spectrometry. *Proc Natl Acad Sci U S A* **88**, 4685-4689 (1991).

14 Aparicio, E., Mathieu, P., Pereira Luppi, M., Almeira Gubiani, M. F. & Adamo, A. M. The Notch signaling pathway: its role in focal CNS demyelination and apotransferrin-induced remyelination. *J. Neurochem.* **127**, 819-836, doi:10.1111/jnc.12440 (2013).

15 Hong, S. & Song, M. R. STAT3 but not STAT1 is required for astrocyte differentiation. *PLoS One* **9**, e86851, doi:10.1371/journal.pone.0086851 (2014).

16 Bizen, N. *et al.* A growth-promoting signaling component cyclin D1 in neural stem cells has antiastrogliogenic function to execute self-renewal. *Stem Cells* **32**, 1602-1615, doi:10.1002/stem.1613 (2014).

17 Faria, M. H., Khayat, A. S., Burbano, R. R. & Rabenhorst, S. H. c -MYC amplification and expression in astrocytic tumors. *Acta Neuropathol* **116**, 87-95, doi:10.1007/s00401-008-0368-0 (2008).

18 Carnemolla, B. *et al.* Identification of a glioblastoma-associated tenascin-C isoform by a high affinity recombinant antibody. *Am J Pathol* **154**, 1345-1352, doi:10.1016/S0002-9440(10)65388-6 (1999).

19 Cau, E. & Blader, P. Notch activity in the nervous system: to switch or not switch? *Neural Dev* **4**, 36, doi:10.1186/1749-8104-4-36 (2009).

20 Wang, Y. Z., Plane, J. M., Jiang, P., Zhou, C. J. & Deng, W. Concise review: Quiescent and active states of endogenous adult neural stem cells: identification and characterization. *Stem Cells* **29**, 907-912, doi:10.1002/stem.644 (2011).

21 Shore, J. & Johnson, R. Axiomatic derivation of the principle of maximum entropy and the principle of minimum cross-entropy. *IEEE Transactions on information theory* **26**, 26-37 (1980).

22 Sottoriva, A. *et al.* Intratumor heterogeneity in human glioblastoma reflects cancer evolutionary dynamics. *Proc Natl Acad Sci U S A* **110**, 4009-4014, doi:10.1073/pnas.1219747110 (2013).

23 Guo, W., Yang, G., Wu, W., He, L. & Sun, M. A parallel attractor-finding algorithm based on Boolean satisfiability for genetic regulatory networks. *PLoS One* **9**, e94258, doi:10.1371/journal.pone.0094258 (2014).

24 Kauffman, S., Peterson, C., Samuelsson, B. & Troein, C. Random Boolean network models and the yeast transcriptional network. *Proc Natl Acad Sci U S A* **100**, 14796-14799, doi:10.1073/pnas.2036429100 (2003).

25 Cedilnik, A., Kosmelj, K. & Blejec, A. The distribution of the ratio of jointly normal variables. *Metodoloski zvezki* **1**, 99 (2004).

26 Baldi, P. & Benz, R. W. BLASTing small molecules—statistics and extreme statistics of chemical similarity scores. *Bioinformatics* **24**, i357-i365 (2008).

27 Jacobs, W. B., Kaplan, D. R. & Miller, F. D. The p53 family in nervous system development and disease. *J. Neurochem.* **97**, 1571-1584, doi:10.1111/j.1471-4159.2006.03980.x (2006).

28 Molchadsky, A., Rivlin, N., Brosh, R., Rotter, V. & Sarig, R. p53 is balancing development, differentiation and de-differentiation to assure cancer prevention. *Carcinogenesis* **31**, 1501-1508 (2010).

29 Grigor'eva, Y. S. & Glazova, M. V. The role of p53 in the proliferation and differentiation of neural stem cells in organotypic cultures of mouse hippocampus. *Neurosci. Behav. Physiol.* **45**, 627 (2015).

30 Tedeschi, A. & Di Giovanni, S. The non-apoptotic role of p53 in neuronal biology: enlightening the dark side of the moon. *EMBO Rep* **10**, 576-583, doi:10.1038/embor.2009.89 (2009).

31 Esdar, C., Milasta, S., Maelicke, A. & Herget, T. Differentiation-associated apoptosis of neural stem cells is effected by Bcl-2 overexpression: impact on cell lineage determination. *Eur. J. Cell Biol.* **80**, 539-553 (2001).

32 Molchadsky, A. & Rotter, V. p53 and its mutants on the slippery road from stemness to carcinogenesis. *Carcinogenesis* **38**, 347-358, doi:10.1093/carcin/bgw092 (2017).

33 Azim, K., Berninger, B. & Raineteau, O. Mosaic Subventricular Origins of Forebrain Oligodendrogenesis. *Front Neurosci* **10**, 107, doi:10.3389/fnins.2016.00107 (2016).

34 Hu, Y. Y. *et al.* Notch signaling contributes to the maintenance of both normal neural stem cells and patient-derived glioma stem cells. *BMC Cancer* **11**, 82, doi:10.1186/1471-2407-11-82 (2011).

35 Stockhausen, M. T., Kristoffersen, K. & Poulsen, H. S. The functional role of Notch signaling in human gliomas. *Neuro Oncol* **12**, 199-211, doi:10.1093/neuonc/nop022 (2010).

36 Wang, L. *et al.* Gamma-secretase represents a therapeutic target for the treatment of invasive glioma mediated by the p75 neurotrophin receptor. *PLoS Biol.* **6**, e289, doi:10.1371/journal.pbio.0060289 (2008).

37 Kamakura, S. *et al.* Hes binding to STAT3 mediates crosstalk between Notch and JAK-STAT signalling. *Nat. Cell Biol.* **6**, 547-554, doi:10.1038/ncb1138 (2004).

38 Armesilla-Diaz, A. *et al.* p53 regulates the self-renewal and differentiation of neural precursors. *Neuroscience* **158**, 1378-1389, doi:10.1016/j.neuroscience.2008.10.052 (2009).

39 Simeone, P. *et al.* A unique four-hub protein cluster associates to glioblastoma progression. *PLoS One* **9**, e103030, doi:10.1371/journal.pone.0103030 (2014).

40 Persson, O. *et al.* Microarray analysis of gliomas reveals chromosomal position-associated gene expression patterns and identifies potential immunotherapy targets. *J Neurooncol* **85**, 11-24, doi:10.1007/s11060-007-9383-6 (2007).

41 Ying, M. *et al.* Regulation of glioblastoma stem cells by retinoic acid: role for Notch pathway inhibition. *Oncogene* **30**, 3454-3467, doi:10.1038/onc.2011.58 (2011).

42 Buschges, R. *et al.* Amplification and expression of cyclin D genes (CCND1, CCND2 and CCND3) in human malignant gliomas. *Brain Pathol* **9**, 435-442; discussion 432-433 (1999).

43 Yeh, T. S., Lin, Y. M., Hsieh, R. H. & Tseng, M. J. Association of transcription factor YY1 with the high molecular weight Notch complex suppresses the transactivation activity of Notch. *J. Biol. Chem.* **278**, 41963-41969, doi:10.1074/jbc.M304353200 (2003).

44 Liao, W. R. *et al.* The CBF1-independent Notch1 signal pathway activates human c-myc expression partially via transcription factor YY1. *Carcinogenesis* **28**, 1867-1876, doi:10.1093/carcin/bgm092 (2007).

45 Mongiardi, M. P. *et al.* c-MYC inhibition impairs hypoxia response in glioblastoma multiforme. *Oncotarget* **7**, 33257 (2016).

46 Baritaki, S. *et al.* YY1 Over-expression in human brain gliomas and meningiomas correlates with TGF-beta1, IGF-1 and FGF-2 mRNA levels. *Cancer Invest.* **27**, 184-192, doi:10.1080/07357900802210760 (2009).

47 Codega, P. *et al.* Prospective identification and purification of quiescent adult neural stem cells from their in vivo niche. *Neuron* **82**, 545-559, doi:10.1016/j.neuron.2014.02.039 (2014).

48 Draberova, E. *et al.* Class III beta-tubulin is constitutively coexpressed with glial fibrillary acidic protein and nestin in midgestational human fetal astrocytes: implications for phenotypic identity. *J. Neuropathol. Exp. Neurol.* **67**, 341-354, doi:10.1097/NEN.0b013e31816a686d (2008).

49 Faigle, R. & Song, H. Signaling mechanisms regulating adult neural stem cells and neurogenesis. *Biochim. Biophys. Acta* **1830**, 2435-2448, doi:10.1016/j.bbagen.2012.09.002 (2013).

50 Joshi, I. *et al.* Notch signaling mediates G1/S cell-cycle progression in T cells via cyclin D3 and its dependent kinases. *Blood* **113**, 1689-1698, doi:10.1182/blood-2008-03-147967 (2009).

51 Pfeuty, B. A computational model for the coordination of neural progenitor self-renewal and differentiation through Hes1 dynamics. *Development* **142**, 477-485, doi:10.1242/dev.112649 (2015).

52 Kageyama, R., Ohtsuka, T., Shimojo, H. & Imayoshi, I. Dynamic Notch signaling in neural progenitor cells and a revised view of lateral inhibition. *Nat. Neurosci.* **11**, 1247-1251, doi:10.1038/nn.2208 (2008).

53 Barton, A. & Fendrik, A. J. Sustained vs. oscillating expressions of Ngn2, Dll1 and Hes1: a model of neural differentiation of embryonic telencephalon. *J. Theor. Biol.* **328**, 1-8, doi:10.1016/j.jtbi.2013.03.004 (2013).

54 Pham-Gia, T., Turkkan, N. & Marchand, E. Density of the ratio of two normal random variables and applications. *Communications in Statistics—Theory and Methods* **35**, 1569-1591 (2006).

55 Fieller, E. C. Some problems in interval estimation. *Journal of the Royal Statistical Society. Series B (Methodological)*, 175-185 (1954).

56 Willan, A. R. & O'Brien, B. J. Confidence intervals for cost-effectiveness ratios: an application of Fieller's theorem. *Health Econ* **5**, 297-305, doi:10.1002/(SICI)1099-1050(199607)5:4<297::AID-HEC216>3.0.CO;2-T (1996).

57 Cancer Genome Atlas Research, N. *et al.* The Cancer Genome Atlas Pan-Cancer analysis project. *Nat. Genet.* **45**, 1113-1120, doi:10.1038/ng.2764 (2013).

58 Cancer Genome Atlas Research, N. Comprehensive genomic characterization defines human glioblastoma genes and core pathways. *Nature* **455**, 1061-1068, doi:10.1038/nature07385 (2008).

59 Dai, Z. *et al.* edgeR: a versatile tool for the analysis of shRNA-seq and CRISPR-Cas9 genetic screens. *F1000Res* **3**, 95, doi:10.12688/f1000research.3928.2 (2014).

60 Straube, J., Huang, B. E. & Cao, K. L. DynOmics to identify delays and co-expression patterns across time course experiments. *Sci Rep* **7**, 40131, doi:10.1038/srep40131 (2017).
